# Tocotrienol metabolites redirect lipid mediator production in innate immune cells towards inflammation resolution

**DOI:** 10.1016/j.apsb.2026.06.056

**Published:** 2026-07-01

**Authors:** Stephan Permann, Elena Brunner, Khaled Alsabil, Markus Werner, Lorenz Waltl, Guillaume Viault, Danilo D’Avino, Bill Perkowski, Anita Siller, Laura Fritsch, Sara Perna, Harald Schennach, Denis Séraphin, Solveigh C. Koeberle, Paul M. Jordan, Fiorentina Roviezzo, Pascal Richomme, Antonietta Rossi, Jean-Jacques Helesbeux, Oliver Werz, Andreas Koeberle

**Affiliations:** a Michael Popp Institute and Center for Molecular Biosciences Innsbruck (CMBI), University of Innsbruck, Innsbruck 6020, Austria; b Department of Pharmaceutical/Medicinal Chemistry, Institute of Pharmacy, Friedrich Schiller University Jena, Jena 07743, Germany; c Univ Angers, SONAS, SFR QUASAV, Angers 49000, France; d Institute of Human Genetics, Medical University of Innsbruck, Innsbruck 6020, Austria; e Department of Pharmacy, School of Medicine and Surgery, University of Naples Federico II, Naples 80131, Italy; f Central Institute for Blood Transfusion and Immunology, Tirol Kliniken GmbH, Innsbruck 6020, Austria; g Institute of Pharmaceutical Sciences and Excellence Field BioHealth, NAWI Graz, University of Graz, Graz 8010, Austria

**Keywords:** Lipoxygenase, Inflammation, Resolution, Lipid mediator, Protectin, Vitamin E, Ferroptosis

## Abstract

Restoring homeostasis in persistent inflammation requires suppressing the inflammatory response and promoting resolution. Currently, no clinically used small molecules intentionally achieve both effects. Endogenous long-chain vitamin E metabolites (LCMs) address inflammation at multiple sites, including pro-inflammatory leukotriene production and cytokine release, thus limiting inflammation. Among LCMs, we here identified *ω*-carboxylates and *ω*-alcohols that suppress leukotriene biosynthesis while enhancing the biosynthesis of prostaglandin E_2_ and specialized pro-resolving mediators, specifically protectins or isomers, proposed to promote inflammation resolution. These LCMs induce a lipid mediator class switch in activated and non-activated human innate immune cells and in a mouse model of self-resolving peritonitis and inhibit ferroptosis. Changes in lipid mediator classes are tightly coordinated between local and systemic sites and follow different kinetics. Mechanistically, LCMs induce polyunsaturated fatty acid release and translocate 15-lipoxygenase-1, a key enzyme in protectin biosynthesis, to particulate locales while also engaging other mechanisms, partially involving cyclooxygenase-2, to increase protectin production. The LCMs also inhibit soluble epoxide hydrolase, reducing the degradation of anti-inflammatory epoxyeicosatrienoic acids, and increase levels of endocannabinoids and dihydroceramides with immunomodulatory and stress-protective functions. These findings provide insights into immunoregulation by tocotrienol metabolites and offer promising lead structures for redirecting lipid mediator profiles from inflammation toward resolution.

## Graphical abstract

From 214 chromanols, this work identified an endogenous tocotrienol metabolite that suppresses ferroptosis, limits inflammation and induces a lipid mediator class switch by inhibiting pro-inflammatory enzymes and enhancing pro-resolving mediator biosynthesis.

**Figure:**
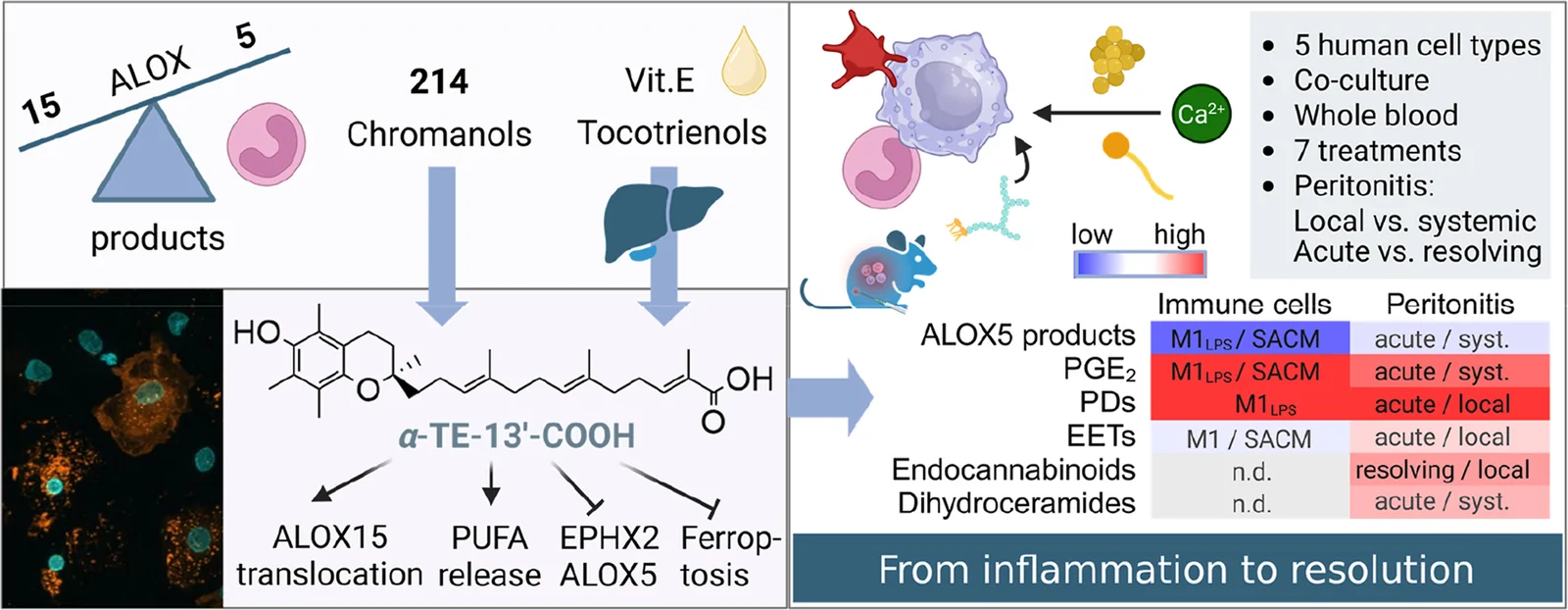

## 1 Introduction

Inflammation is a multiple-stage physiological process aimed at eliminating damaged cells and pathogens and restoring homeostasis, and is orchestrated by temporally and spatially tightly controlled mediators^1^^,^^2^. The initial phase of acute inflammation is driven by overall pro-inflammatory cytokines and lipid mediators, mainly arachidonic acid (AA)-derived prostaglandins (PG) and leukotrienes (LT), and is characterized by the recruitment of neutrophils, monocytes, macrophages, and lymphocytes^3^^,^^4^. The physiological roles of these diverse lipid mediators are complex and include both pro-inflammatory and anti-inflammatory activities, depending not only on their molecular structure, but also on their kinetics, local and systemic concentrations, and the receptor repertoire, signaling capacity, and metabolic state of neighboring cells^1^^,^^2^^,^^5^, ^6^, ^7^. For example, PGE_2_ is the major PG that promotes inflammation, fever and pain, but also has homeostatic and even anti-inflammatory properties in the later phase of inflammation^8^, ^9^, ^10^ and, at moderately elevated concentrations, initiates a lipid mediator class switch toward resolution^11^.

Resolution of inflammation actively terminates the pro-inflammatory reaction and activates a pro-resolving program in immune and non-immune cells^1^^,^^2^^,^^12^. Integral to the resolution of inflammation are distinct subpopulations of innate immune cells, such as M2-like macrophages, which express high levels of 15-lipoxygenase (ALOX15, 15-LOX)^13^ and, in concert with other cells, induce a lipid mediator class switch towards specialized pro-resolving mediators (SPM), such as lipoxins (LX), resolvins (Rv), protectins (PD), and maresins (MaR), which suppress pro-inflammatory cytokine release, interfere with neutrophil trafficking, stimulate efferocytosis, promote bacterial clearance, and support tissue regeneration^14^^,^^15^. Whether low abundant SPM, especially tri-hydroxylated polyunsaturated fatty acids (PUFA), reach effective concentrations *in vivo* under specific disease conditions is currently under debate^16^, ^17^, ^18^. Failure of resolution results in low-grade chronic inflammation, which can acquire a systemic component and is implicated in the development and progression of a variety of lifestyle diseases, such as diabetes, cardiovascular disease, chronic liver diseases, neurodegeneration, and cancer^19^, ^20^, ^21^, ^22^. While clinical approaches to treat inflammatory diseases are dominated by strategies that suppress the inflammatory response (and potentially also inflammation resolution), novel experimental approaches are emerging that actively promote inflammation resolution^2^^,^^14^^,^^23^, ^24^, ^25^, ^26^, ^27^, ^28^, ^29^, ^30^, ^31^, ^32^, among others, by upregulating SPM biosynthesis through small molecules^33^, ^34^, ^35^, ^36^, ^37^, ^38^, ^39^, ^40^, ^41^, ^42^, ^43^, ^44^. Further studies are needed to clarify whether such approaches fulfill the promise of greater efficacy against chronic inflammatory diseases and fewer side effects compared to non-steroidal anti-inflammatory drugs or glucocorticoids.

The biosynthesis and signal transduction of AA-derived lipid mediators, such as prostanoids and LT, have been studied for decades^4^^,^^7^. Internal and external stimuli trigger Ca^2+^ influx or phosphorylation cascades leading to the translocation of cytosolic phospholipase A_2*α*_ (PLA2G4A, cPLA_2*α*_) to cellular membranes^45^. PLA2G4A releases AA and other PUFA from membrane phospholipids, thereby providing these fatty acids to key enzymes in the biosynthesis of lipid mediators^46^: i) cyclooxygenase (PTGS, COX) isoenzymes that initiate prostanoid biosynthesis^47^, ii) 5-lipoxygenase (ALOX5, 5-LOX) that leads to LT formation but also contributes to the biosynthesis of specific SPM, such as LX and Rv^14^^,^^48^ iii) and cytochrome P_450_ monooxygenases that produce hydroxy- and epoxy-fatty acids, most notably epoxyeicosatrienoic acids (EET), which limit inflammation and suppress apoptosis^49^, ^50^, ^51^. Many other immunoregulatory lipid mediators are produced in parallel, including endocannabinoids and bioactive sphingolipids, to name a few^52^^,^^53^.

SPM biosynthesis during inflammation resolution is instead dominated by ALOX enzymes, with ALOX15 isoenzymes at the forefront. It requires sequential, partly transcellular peroxidation followed by subsequent reduction or rearrangement leading to di- and tri-hydroxylated PUFA with defined stereochemistry^14^. For example, PD1 is derived from the ALOX15-derived precursor 17*S*-HpDHA, which is first converted to the 16*S*,17*S*-epoxide, again by ALOX15, and then into the 10*S*,17*S*-dialcohol by an unknown hydrolase^54^. How SPM formation is regulated, and which isoenzymes contribute to the different synthesis steps in individual cell types is still diffuse. Recent evidence suggests that the Ca^2+^-induced translocation of ALOX15 to particulate locales within the cytoplasm might play an important role^34^^,^^54^^,^^55^.

To combat complex diseases such as inflammation, strategies that target multiple features and combine suppression of inflammation with improved resolution have come into focus^2^^,^^10^^,^^32^^,^^56^. This preferred pharmacological profile has sparked our interest in long-chain vitamin E metabolites (LCMs), which possess several of these features and contribute to the immunoregulatory and anti-inflammatory activity of vitamin E to an unknown extent^57^^,^^58^. LCMs derived from tocopherols and tocotrienols are classified into *α*-, *β*-, *γ*-, and *δ*-isoforms based on the methylation of the chromanol core, analogous to the original tocopherol and tocotrienol forms of vitamin E. In addition, they are *ω*-oxidized at the side chain, either to alcohols or carboxylates, and optionally truncated by *β*-oxidation during liver passage^58^. LCMs reach nanomolar plasma and tissue concentrations, depending on the availability of vitamin E and defined by interindividual differences in the ability to metabolize vitamin E^59^, ^60^, ^61^. In cell-based and animal studies (partially at supraphysiological concentrations), we and others have shown that LCM inhibit ALOX5^60^ and microsomal prostaglandin E_2_ synthase-1^62^—the terminal enzyme of PGE_2_ biosynthesis^63^, suppress TLR4 signaling associated with nuclear factor-*κ*B activation, pro-inflammatory cytokine release and PTGS2 and inducible nitric oxide synthase expression^64^, ^65^, ^66^, activate nuclear receptors, specifically the peroxisome proliferator-activated receptor *γ*^67^ and the pregnane X receptor^68^^,^^69^, and potentially elevate systemic SPM levels^60^ along with wound healing^70^, in addition to regulating lipid metabolism with implications for foam cell formation^71^^,^^72^ and cancer cell death^73^. In addition, *δ*-garcinoic acid (**4**), an *ω*-carboxylated tocotrienol, has recently been shown to enhance efferocytosis and inflammation resolution in mouse bone marrow-derived macrophages and in an experimental model of colitis^74^. This activity is dependent on Nrf2 and the induction of efferocytosis receptors, and is potentially linked to the upregulation of Rv and LX. While some LCMs combine several of these activities, none of them excel in all aspects. Structural optimization has led to potent second- and third-generation ALOX5 inhibitors that are orally active and metabolically stable, but have lost their ability to increase the production of SPM precursors^75^^,^^76^.

To systematically explore the potential of LCMs and their derivatives to combine anti-inflammatory and pro-resolving properties, we screened an in-house library of 214 chromanols for potent inhibition of ALOX5 while inducing ALOX15-dependent SPM precursor formation. We identified the *ω*-carboxylate *α*-TE-13′-COOH (**2**, *α*-garcinoic acid), a hepatic metabolite of *α*-tocotrienol, as a direct inhibitor of ALOX5 that preferentially upregulates PD formation in human innate immune cells, particularly in polarized macrophage subsets. In a self-resolving murine inflammation model, metabolite **2** limits immune cell infiltration associated with favorable changes in the local and systemic lipid mediator profile, such as highly elevated levels of PD and/or isomers as well as EET, endocannabinoids and specific bioactive sphingolipids. Mechanistically, metabolite **2** acts independently of innate immune cell activation by increasing the availability of free docosahexaenoic acid (DHA) while promoting the translocation of ALOX15 to subcellular membrane compartments. As expected for lipophilic chromanols, **2** exhibits potent anti-ferroptotic activity, even surpassing the parent compound *α*-tocopherol, which may contribute to mitigating necroinflammatory processes. Thus, compound **2** establishes an anti-inflammatory/pro-resolving lipid mediator profile both in activated and non-activated innate immune cells, with potential implications for the claimed health benefits of tocotrienols over tocopherols and the development of LCM-inspired drugs that resolve inflammation.

## 2 Materials and methods

### 2.1 Materials

*α*-T (≥96%) was purchased from Enzo Life Sciences (Loerrach, Germany), *β*-T (>99%), *γ*-T (≥96%), *δ*-T (≥90%) were obtained from Sigma–Aldrich (Deisenhofen, Germany), and *α*-TE (≥98%), *β*-TE (≥98%), *γ*-TE (≥98%), *δ*-TE (≥98%) were from Cayman Chemicals (Ann Arbor, MI, USA). The phytochemical study of a DCM extract obtained from the stem barks of *Garcinia amplexicaulis* led to the isolation and characterization of *γ*-TE-13′-COOH^62^, *δ*-TE-13′-COOH (**4**)^77^, *γ*-TE-12a′-CH_2_OH (**8**)^78^, *δ*-TE-13′-CH_2_OH^79^, *δ*-TE-12a′-CH_2_OH (**7**)^80^, *γ*-TE-12a′,13′-diCH_2_OH^78^, *δ*-TE-12a′,13′-diCH_2_OH^78^, and *δ*-TE-12a′-CH_2_OH-13′-CHO^79^.

The (semi)syntheses of *α*-T-13′-COOH (**1**), *α*-TE-13′-COOH (**2**), *β*-TE-13′-COOH (**3**), *δ*-TE-12a′-COOH (**5**), *β*-DE-13′-COOH (**6**), *δ*-T-13′-COOH, *α*-T-13′-OH, *δ*-T-13′-OH, *α*-TE-13′-CH_2_OH, *β*-TE-13′-CH_2_OH, *α*-TE-13′-CH_2_OH, *β*-TE-13′-CH_2_OH, *α*-TE-12a′,13′-diCH_2_OH, *δ*-TE-12a′,13′-diCH_2_OH, *β*-TE-12a′,13′-diCH_2_OH, *α*-T-11′-COOH, and *δ*-TE-11′-COOH are described in detail in the Supporting Information. LCMs and small molecule inhibitors were dissolved in DMSO, stored in the dark at −20 °C under argon, and freeze–thaw cycles were kept to a minimum. Fatty acids, lipid mediators, and standards were purchased from Cayman Chemicals, dissolved in methanol and aliquots were stored in the dark at −80 °C under argon. HPLC-grade solvents used for extraction, dilution, and ultra-performance liquid chromatography (UHPLC) were bought from VWR (Darmstadt, Germany) or Fisher Scientific (Waltham, MA, USA). Ultrapure water was obtained using a Sartorius Arium 611 UV water purification system (Göttingen, Germany). Cell culture flasks and 6-, 12-, and 96-well plates were purchased from Greiner (Kremsmünster, Austria), and coverslips were provided by Thermo Fisher Scientific (Waltham, MA, USA).

### 2.2 Isolation of human blood cells

Peripheral blood mononuclear cells and polymorphonuclear leukocytes (PMNL) were isolated from leukocyte reduction system chamber (LRSC) of platelet apheresis donors provided by the Central Institute for Blood Transfusion and Immunology of Tirol Kliniken GmbH (Austria) as previously described^81^. Briefly, residual blood was collected after written informed consent for residual sample use from healthy volunteers (male and female) who were physically examined by trained medical personnel and who meet the requirements of the Austrian Blood Donation Regulation (BGBI. II Nr. 217/2022). Immune cell concentrates were diluted in PBS pH 7.4 containing 12.5 mmol/L citrate and 14 mmol/L glucose and subjected to equilibrium density gradient centrifugation (400 × *g*, 20 min, room temperature) using Histopaque-1077 (Sigma–Aldrich, St. Louis, MO, USA). Peripheral blood mononuclear cells (PBMC) recovered from the interphase were washed and the remaining erythrocytes were removed by hypotonic lysis. PMNL were obtained from the pellet after density centrifugation, again after lysis of erythrocytes in water. Immune cells were counted using a Vi-Cell XR Cell Viability Analyzer (Beckmann Coulter, Brea, CA).

Alternatively, PMNL and platelets were isolated from leukocyte concentrates provided by the Institute for Transfusion Medicine at the University Hospital Jena (Germany) according to a standardized procedure^37^. Male and female blood donors (18–65 years) were informed about and provided written informed consent for the experimental studies, donated blood regularly (every 8–12 weeks), and were declared healthy by a clinician. In particular, they had no apparent infections, inflammatory disorders, or acute allergic reactions, and had not taken anti-inflammatory drugs or antibiotics for more than 10 days prior to blood collection. After dextran sedimentation and density gradient centrifugation on lymphocyte separation medium (Histopaque-1077, Sigma–Aldrich), PMNL were obtained from the pellet as described above, while platelets were collected from the upper phase and washed twice as described^75^.

Experiments involving blood cells were conducted in compliance with the German Transfusion Law (TFG, BGBl. I S. 2169) and Austrian Blood Donation Regulation (BGBI. II Nr. 217/2022) for blood donation as well as institutional guidelines, and were approved by the ethics committees of the University Hospital Jena (March 3, 2017; #5050–01/17) or the Medical University Innsbruck (June 19, 2020; #1041/2020).

### 2.3 Analysis of 15-hydroxyeicosatetraenoic acid (HETE) formation in PMNL

Freshly isolated PMNL (1 × 10^7^) were suspended in PBS pH 7.4 containing 1 mg/mL glucose. Cells were pre-incubated with vehicle (DMSO, 0.1%) or LCMs for 10 min at 37 °C and then treated with 20 μmol/L AA and 2.5 μmol/L A23187 for 10 min at 37 °C. The reaction was stopped by adding an equal volume of methanol. 15-HETE was extracted and analyzed by reversed phase HPLC as described^60^.

### 2.4 Structure-similarity network

Correlation networks based on structural similarity were created with Cytoscape 3.9.1 (Cytoscape Consortium)^75^ using the chemViz2 1.1.1 plugin (by John “Scooter” Morris, UCSF; Dazhi Jiao, Indiana University)^82^. The network is organized in a yFiles organic layout, with the nodes representing individual LCMs. The connecting edges indicate Tanimoto coefficients (>0.92), a measure of structural similarity, which were calculated by chemViz2 1.1.1 using the SMILES codes of the LCMs. In the network, side chain saturation is visualized by the shape of the nodes, the color differentiates between functional groups in the *ω*-position, and the node size indicates the production of 15-HETE (as percentage of vehicle control) in A23187-and AA-challenged PMNL.

### 2.5 Monocyte to macrophage differentiation and macrophage polarization

Freshly isolated PBMC were differentiated into macrophages with 20 ng/mL granulocyte macrophage-colony stimulating factor (GM-CSF; for further polarization to M1-like phenotypes) or macrophage-colony stimulating factor (M-CSF; for further polarization to M2-like and LPS^+^ phenotypes) (HiSS Diagnostics GmbH, Freiburg, Germany or Peprotech, Hamburg, Germany) for 6 days in RPMI 1640 medium (Sigma–Aldrich) containing 10% fetal calf serum (FCS, Sigma–Aldrich), 100 U/mL penicillin (Fisher Scientific) and 100 μg/mL streptomycin (Fisher Scientific) at 37 °C and 5% CO_2_^75^^,^^83^. Monocyte-derived macrophages were subsequently polarized for a further 48 h to either M1-like, M2-like or M2_LPS_ phenotypes by stimulation with i) 100 ng/mL lipopolysaccharide (LPS; *Escherichia coli* O127:B8, Sigma–Aldrich) and 20 ng/mL interferon-*γ* (Peprotech, Hamburg, Germany), ii) 20 ng/mL interleukin (IL)-4 (Peprotech), and iii) 100 ng/mL LPS and 20 ng/mL IL-4 (Peprotech), respectively. To study the effect of LCM on macrophage polarization, vehicle (DMSO, 0.1%) or LCMs at the indicated concentrations were added 15 min before LPS, interferon-*γ*, and IL-4.

### 2.6 Preparation of Staphylococcus aureus-conditioned medium (SACM)

*Staphylococcus aureus* LS1 was cultured in brain heart infusion medium (37 g/L, Lactan, Graz, Austria) for 18 h at 37 °C^84^. The supernatant (3400 × *g*, 10 min, room temperature) was sterile-filtrated through a Rotilabo Syringe Filter (PVDF, 0.22 μm, Roth, Karlsruhe, Germany) and stored at 4–7 °C for up to 10 days. The activity of SACM to induce lipid mediator production was verified for each batch in human macrophages (M1, M2, M2_LPS_) by visually inspecting LTB_4_ production (peak height).

Alternatively, *Staphylococcus aureus* 6850 wt (stock OD_660nm_ = 1) was grown on Columbia blood agar with sheep blood. After 24 h at 37 °C one colony was picked from and was added to 50 mL brain heart infusion medium (Sigma–Aldrich, Taufkirchen, Germany). Then it was cultured for 24 h at 37 °C under shaking. The OD_660_ of the medium was measured (Biochrom Ultrospec 30 Cell Density Meter, Harvard Bioscience, Holliston, MA, USA) and adjusted to OD_660nm_ of 0.05 in fresh 50 mL brain heart infusion medium. Again, it was subjected to 37 °C for 24 h under shaking. The medium was centrifuged (2600 × *g*, 3 min, room temperature) and the supernatant was sterile filtered through a Millipore Millex-GP Filter (0.22 mm; Merck, Darmstadt, Germany). The SACM was stored at 4 °C and was used under sterile conditions not exceeding use two weeks after preparation.

### 2.7 Stimulation of PBMC for lipid mediator profiling

PBMC (5 × 10^6^) were pre-incubated with vehicle (DMSO, 0.1%) or LCMs in RPMI 1640 medium containing 5% FCS, 100 U/mL penicillin and 100 μg/mL streptomycin (1.5 mL) and stimulated with LPS (*Escherichia coli* O127:B8, Sigma–Aldrich) for 24 h at 37 °C and 5% CO_2_. Alternatively, PBMC (5 × 10^6^) pre-incubated with vehicle (DMSO, 0.1%) or LCMs and were challenged with 2.5 μmol/L of the Ca^2+^-ionophore A23187 (Cayman Chemicals) in PBS pH 7.4 (1 mL) supplemented with 1 mmol/L CaCl_2_ at 37 °C. Lipid mediator production was stopped by adding 2 mL ice-cold methanol containing deuterium-labeled internal standards (Cayman Chemicals): 200 pg *d*_8_-5S-HETE, 200 pg *d*_4_-LTB_4_, 200 pg *d*_5_-lipoxin (LX)A_4_, 200 pg *d*_5_- RvD2, 200 pg *d*_4_-PGE_2_, and 2000 pg *d*_8_-AA. Proteins were precipitated at −20 °C for at least 16 h before samples were subjected to metabololipidomics sample preparation and UHPLC–MS/MS analysis.

### 2.8 Stimulation of human M1-like, M2-like and M2_LPS_ macrophages for lipid mediator profiling

Macrophages (2 × 10^6^) in PBS (pH 7.4 with 1 mmol/L CaCl_2_, 2 mL) were pre-incubated with vehicle (DMSO, 0.1%) or LCMs for 15 min at 37 °C and 5% CO_2_ and challenged with 0.1% SACM or left untreated for 180 min. To study lipid mediator production in the presence of exogenous fatty acids (Fig. 6C and D), macrophages were supplemented with vehicle (DMSO, 0.1%), 20 μmol/L DHA, or 20 μmol/L AA immediately prior to the addition of SACM. For the kinetic studies in Fig. 6F and H, Supporting Information Fig. S7, macrophages (2 × 10^6^) were pre-incubated with vehicle (DMSO, 0.1%) or LCMs in RPMI 1640 medium containing 10% FCS, 100 U/mL penicillin, and 100 μg/mL streptomycin for the indicated time. After changing the culture medium to PBS pH 7.4 (containing 1 mmol/L CaCl_2_, 1.5–2 mL), the cells were incubated without further treatment or additionally stimulated with SACM as described above. To investigate the role of PTGS2 in the induction of PD biosynthesis (Fig. 7B, Supporting Information Fig. S8), M2-like macrophages in RPMI 1640 medium containing 10% FCS, 100 U/mL penicillin, and 100 μg/mL streptomycin were treated with vehicle (PBS pH 7.4, 1 mmol/L CaCl_2_) or 500 μmol/L acetylsalicylic acid (Gatt-Koller GmbH, Absam, Austria). After 60 min at 37 °C in 5% CO_2_, vehicle (DMSO, 0.1%), LCMs, and/or celecoxib (3 μmol/L, Cayman Chemicals) were added and the incubation continued for another 15 min or 24 h. The culture medium was replaced with 1.5 mL PBS pH 7.4 containing 1 mmol/L CaCl_2_, vehicle (0.1% DMSO), acetylsalicylic acid (500 μmol/L), or celecoxib (3 μmol/L), either alone or in combination with LCMs, before cells were stimulated with SACM as outlined above.

**Figure 1:**
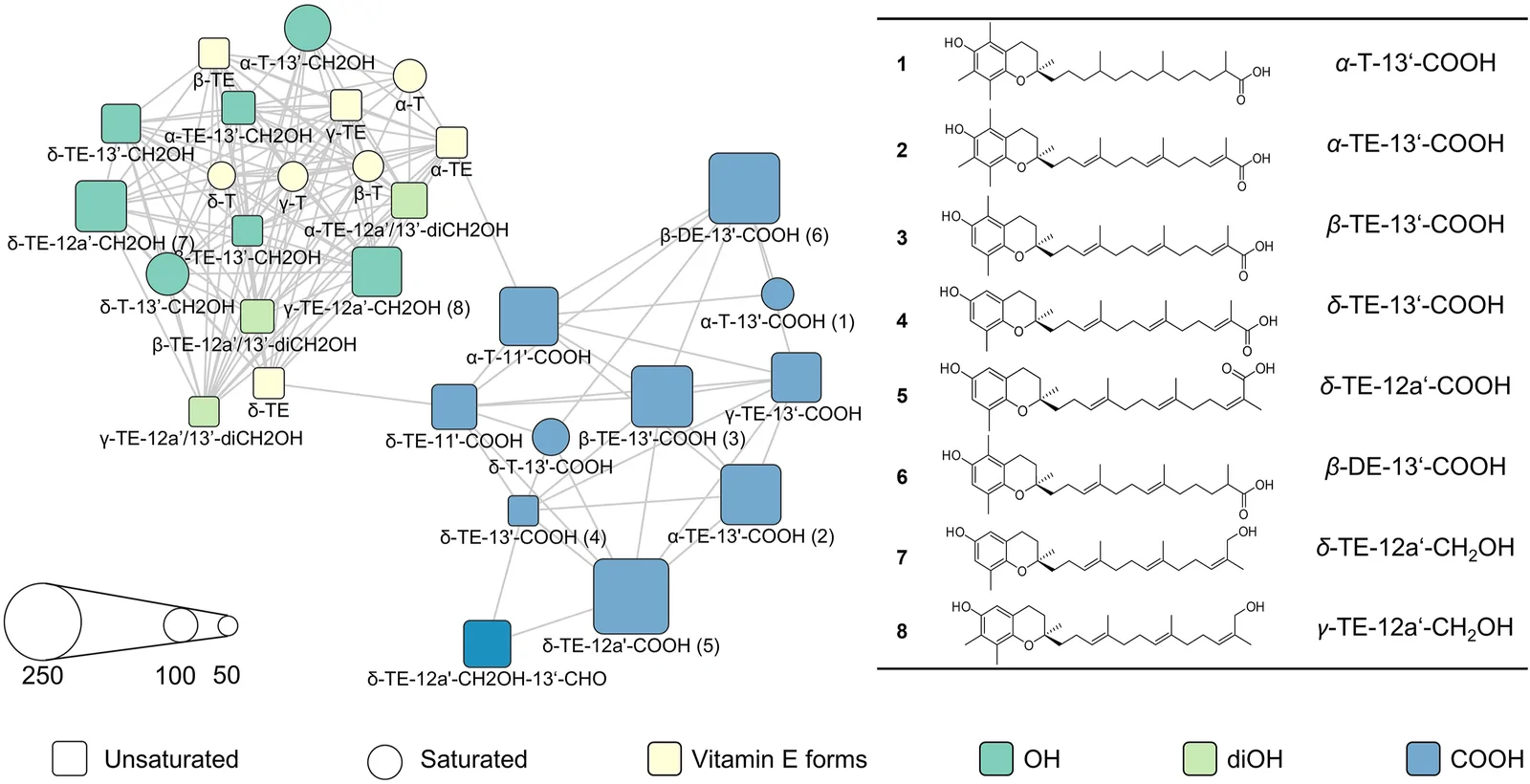
Similarity network of long-chain vitamin E metabolites (LCMs) displaying 15-hydroxyeicosatetraenoic acid (15-HETE) formation in polymorphonuclear leukocytes (PMNL). Structural similarity between LCMs is calculated using Tanimoto similarity score and visualized as network. Nodes representing each compound are connected with edges (Tanimoto coefficients >0.92) in a yFiles organic network. Saturation of side chain is visualized with shape of nodes, color represents distinctive functional groups and natural vitamin E forms and size displays the relative amount of 15-HETE formation (% of control, DMSO) in PMNL. Data on vitamin E forms and metabolites has partially been extracted from previous studies^60^^,^^75^, as detailed in Supporting Information Table S1.

**Figure 2:**
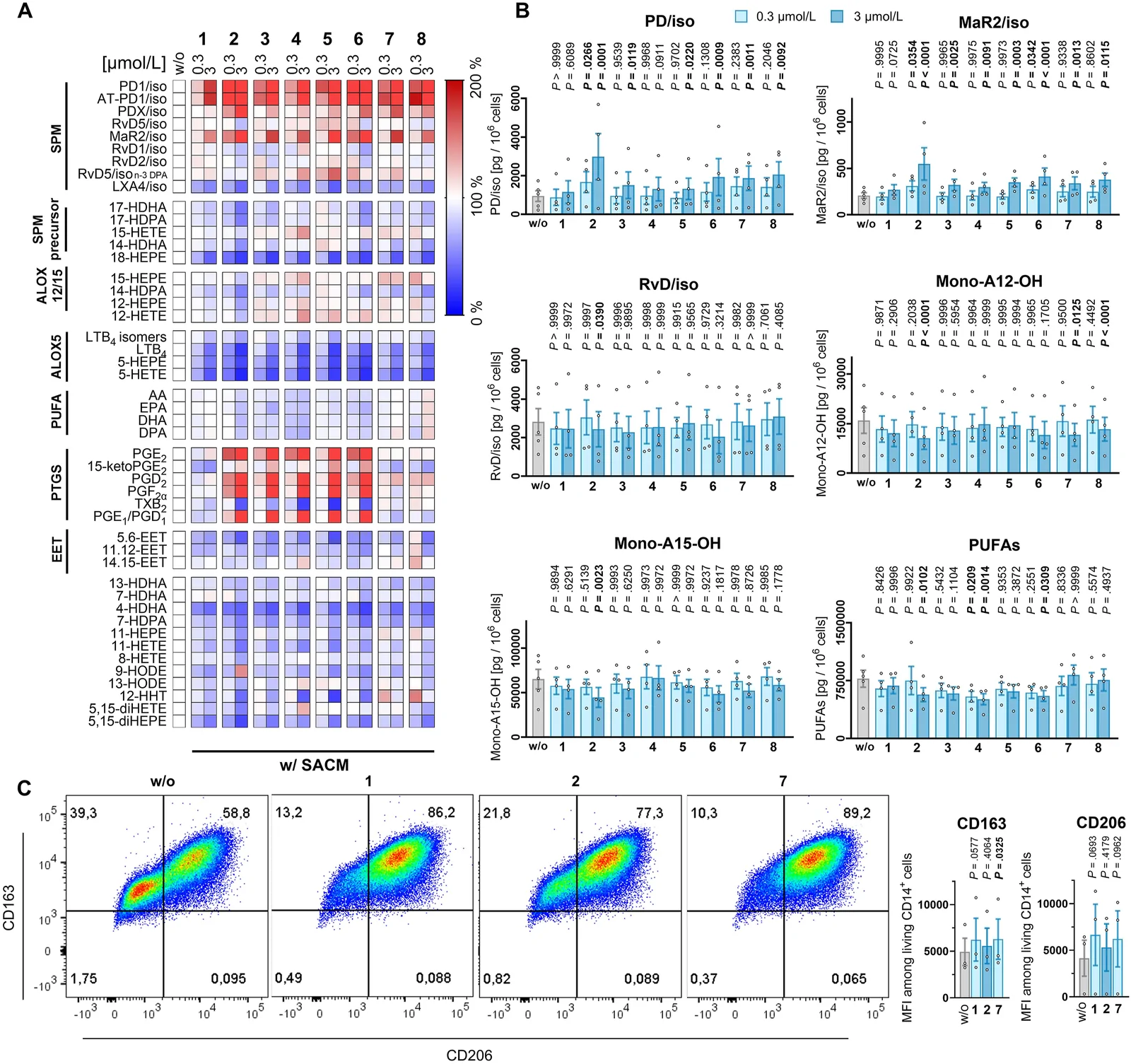
LCMs induce a lipid mediator class switch in activated human M2 macrophages without changing polarization. (A, B) Monocyte-derived M2-like macrophages (2 × 10^6^) pre-treated with vehicle (DMSO, 0.1%, ‘*w*/*o*’) or LCMs for 15 min were stimulated with *Staphylococcus aureus*-stimulated medium (SACM; 1%) for 180 min (w/SACM). (A) The heatmap shows changes in lipid mediator levels relative to the vehicle control. The labels ALOX15, ALOX12, ALOX5, and PTGS indicate products of these enzymes. (B) Levels of total protectin (PD)/iso, maresin (MaR)2/iso, resolving (Rv)D/iso, mono-A12-OH, mono-A15-OH, and polyunsaturated fatty acids (PUFA). (C) Monocyte-derived macrophages (2 × 10^6^) were pre-treated with vehicle (DMSO, 0.1%) or LCMs before the addition of IL-4 and incubated for 48 h. The cytograms show the distribution of CD163 and CD206 expression in the cell population, which is quantified in the adjacent bar charts; MFI, mean fluorescence intensity. Mean (A) or mean ± SEM and single data (B, C) from *n* = 4 (A, B) and *n* = 3 (C) independent experiments. *P* values *vs.* vehicle control; repeated measures one-way ANOVAs with Dunnett’s *post hoc* tests of log data.

**Figure 3:**
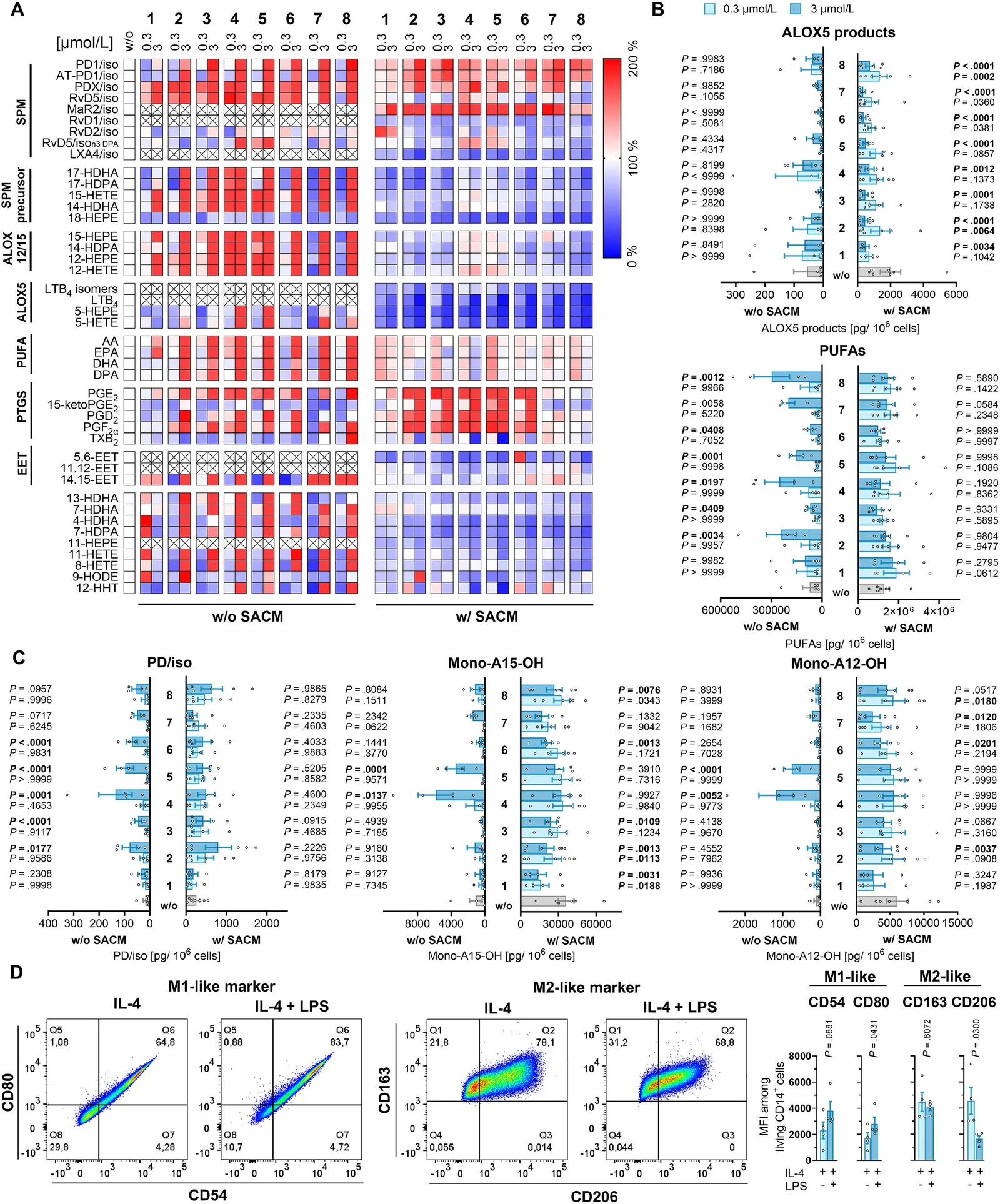
LCMs induce a lipid mediator class switch in activated human M2_LPS_ macrophages which represents an hybrid subtype of polarized macrophages. (A–C) Monocyte-derived M2_LPS_ macrophages (2 × 10^6^) pre-treated with vehicle (DMSO, 0.1%, ‘w/o’) or LCMs for 15 min were stimulated with SACM (1%) (w/SACM) or left untreated (w/o SACM) for 180 min. (A) The heatmap shows changes in lipid mediator levels relative to the vehicle control. The labels ALOX15, ALOX12, ALOX5, and PTGS indicate products of these enzymes. (B, C) Levels of total ALOX5 products, PUFA, PD/iso, mono-A12-OH and mono-A15-OH. (D) Monocyte-derived macrophages (2 × 10^6^) were pre-incubated with vehicle (DMSO, 0.1%) or LCMs for 15 min before being treated with IL-4 ± LPS for 48 h. The cytograms show the distribution of CD80 *vs.* CD54 and CD163 *vs.* CD206 expression in the cell population, which is quantified in the adjacent bar charts; MFI, mean fluorescence intensity. Mean (A) or mean ± SEM and single data (B–D) from *n* = 3–6 (A–C) and *n* = 3 (D) independent experiments. *P* values *vs.* vehicle control; repeated measures one-way ANOVAs with Dunnett’s *post hoc* tests of log data.

**Figure 4:**
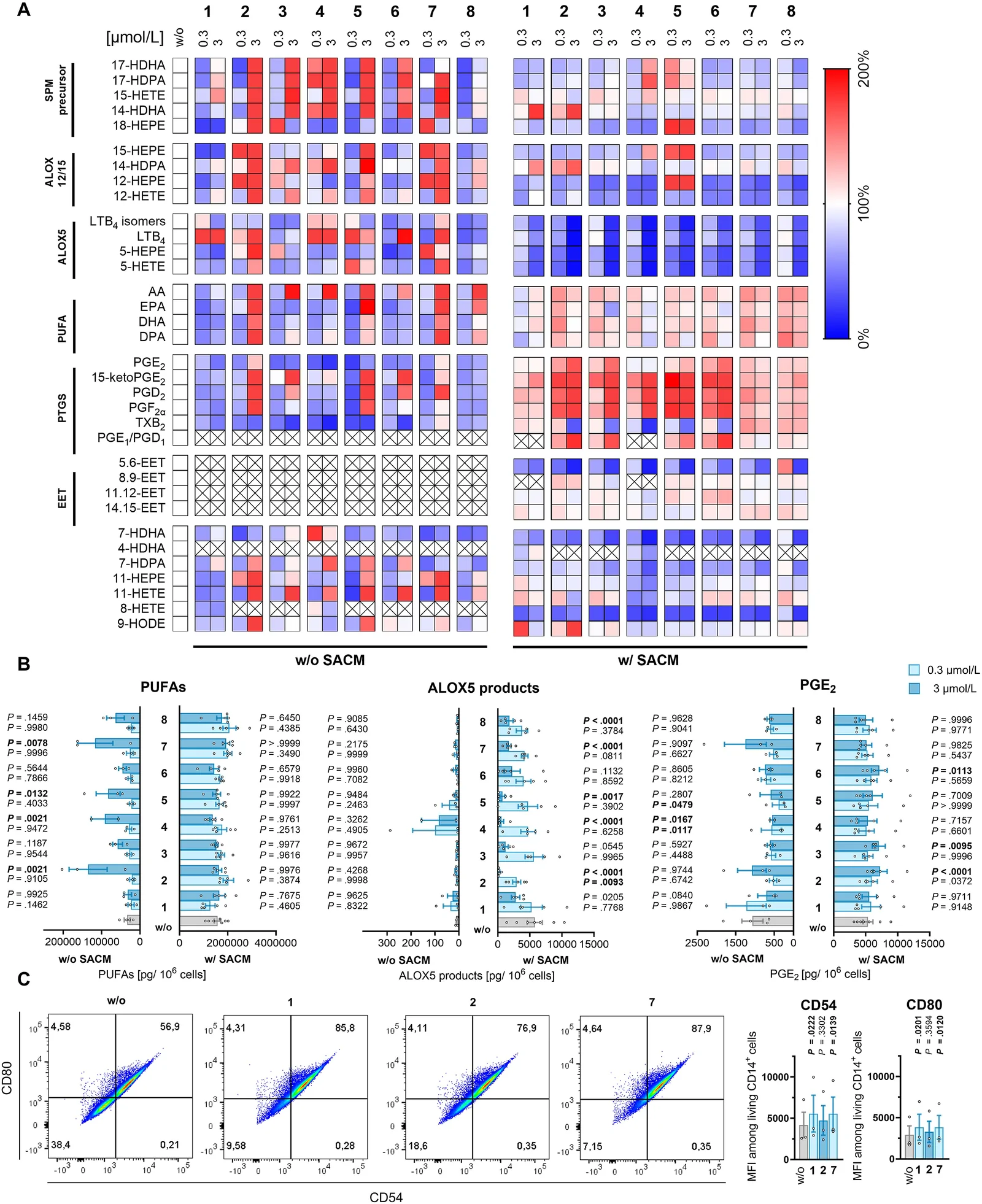
LCMs induce a lipid mediator class switch in activated human M1-like macrophages without changing polarization. (A, B) Monocyte-derived M1-like macrophages (2 × 10^6^) pre-treated with vehicle (DMSO, 0.1%, ‘w/o’) or LCMs for 15 min were stimulated with SACM (1%) (w/SACM) or left untreated (w/o SACM) for 180 min. (A) The heatmap shows changes in lipid mediator levels relative to the vehicle control. The labels ALOX15, ALOX12, ALOX5, and PTGS indicate products of these enzymes. (B) Levels of total PUFA, ALOX5 products and prostaglandin (PG)E_2_. (C) Monocyte-derived macrophages (2 × 10^6^) were pre-incubated with vehicle (DMSO, 0.1%) or LCMs for 15 min before being treated with LPS + interferon-*γ* for 48 h. The cytograms show the distribution of CD80 *vs.* CD54 expression in the cell population, which is quantified in the adjacent bar charts; MFI, mean fluorescence intensity. Mean (A) or mean ± SEM and single data (B, C) from *n* = 4 (A, B) and *n* = 3 (C) independent experiments. *P* values *vs.* vehicle control; repeated measures one-way ANOVAs with Dunnett’s *post hoc* tests of log data.

**Figure 5:**
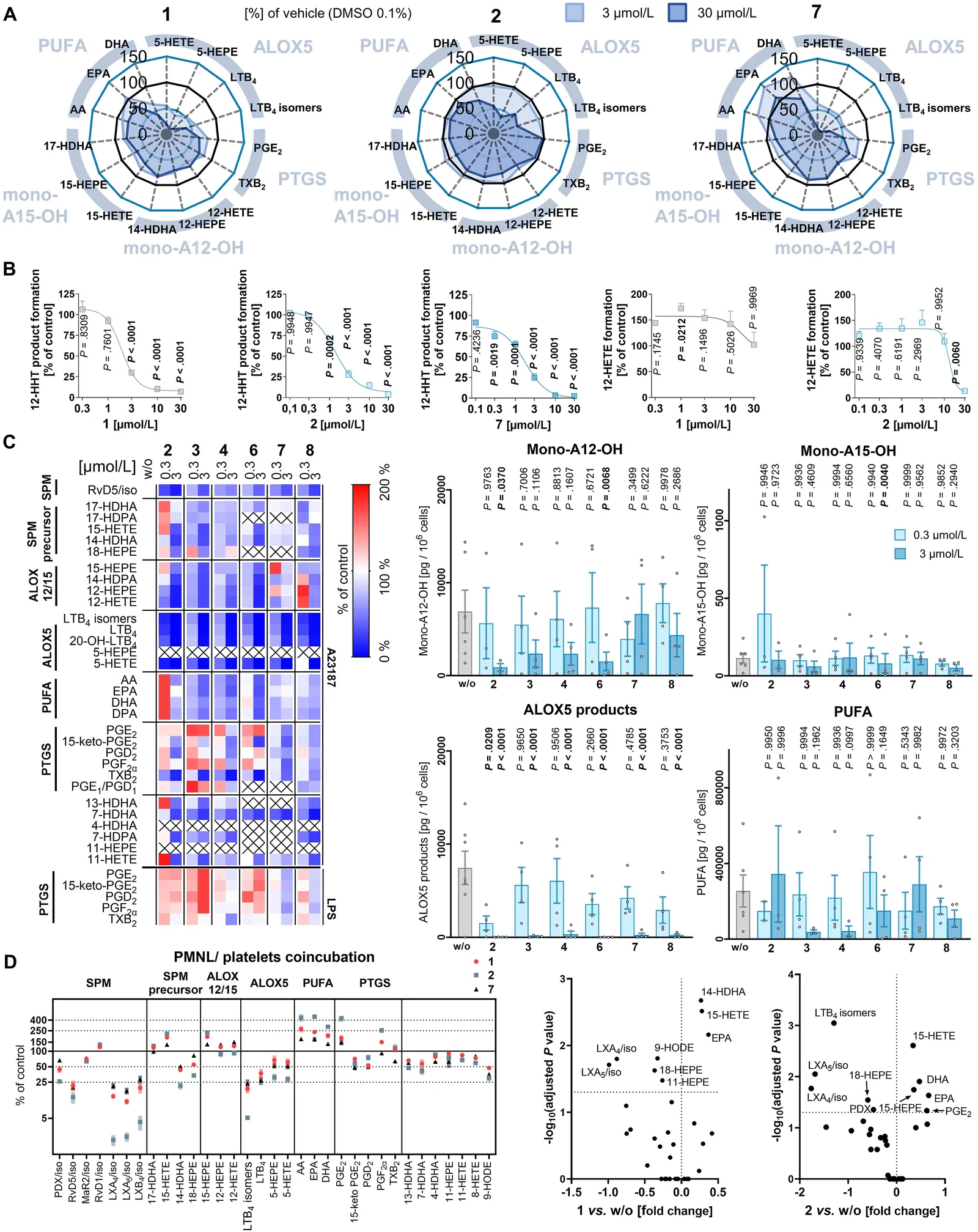
Effects of LCMs on the lipid mediator profile in human whole blood, platelets, peripheral blood mononuclear cells (PBMC) and PMNL co-incubated with platelets. (A) Human whole blood (1 mL) was pre-incubated with LCMs or vehicle (DMSO, 0.1%, ‘w/o’) for 15 min, followed by stimulation with SACM (10%) for 180 min. (B) Human platelets (1 × 10^8^) were pre-incubated with the LCMs or vehicle (DMSO, 0.1%) for 4.5 min and then treated with arachidonic acid (AA; 5 μmol/L). Amounts of 12-hydroxyheptadecatrienoic acid (12-HHT) or 12-hydroxyeicosatetraenoic acid (12-HETE). (C) Human PBMC (5 × 10^6^) were incubated with LCMs (at 0.3 or 3 μmol/L) or vehicle (DMSO, 0.1%, ‘w/o’) and either treated with A23187 (2.5 μmol/L) for 10 min or with lipopolysaccharide (LPS; 100 ng/mL) for 24 h. The heatmap shows changes in lipid mediator levels relative to the vehicle control. The labels ALOX15, ALOX12, ALOX5, and PTGS indicate products of these enzymes. The bar charts show the levels of total mono-A12-OH, mono-A15-OH, ALOX5 products and PUFA in A23187-treated PBMC. (D) Human PMNL (1 × 10^6^) and platelets (2.5 × 10^8^) were incubated together, treated with LCMs (3 μmol/L) or vehicle (DMSO, 0.1%) for 5 min and then stimulated with 1 mmol/L CaCl_2_ and SACM (1%) for 180 min. The dot plot shows percentage changes in lipid mediator levels for each LCM separated by color. The Volcano plots show the mean fold-changes relative to vehicle control and the negative log_10_(adjusted *P* value) calculated *vs.* vehicle control; two-tailed, multiple paired Student *t* tests with correction for multiple comparisons using the Bonferroni-Dunn method (alpha level: 0.05). Mean (A, C, D) or mean ± SEM (B, D) and single data (C) from *n* = 3–4 (A–D) independent experiments. *P* values *vs.* vehicle control; repeated measures one-way ANOVAs with Dunnett’s *post hoc* tests of log data (B, C).

**Figure 6:**
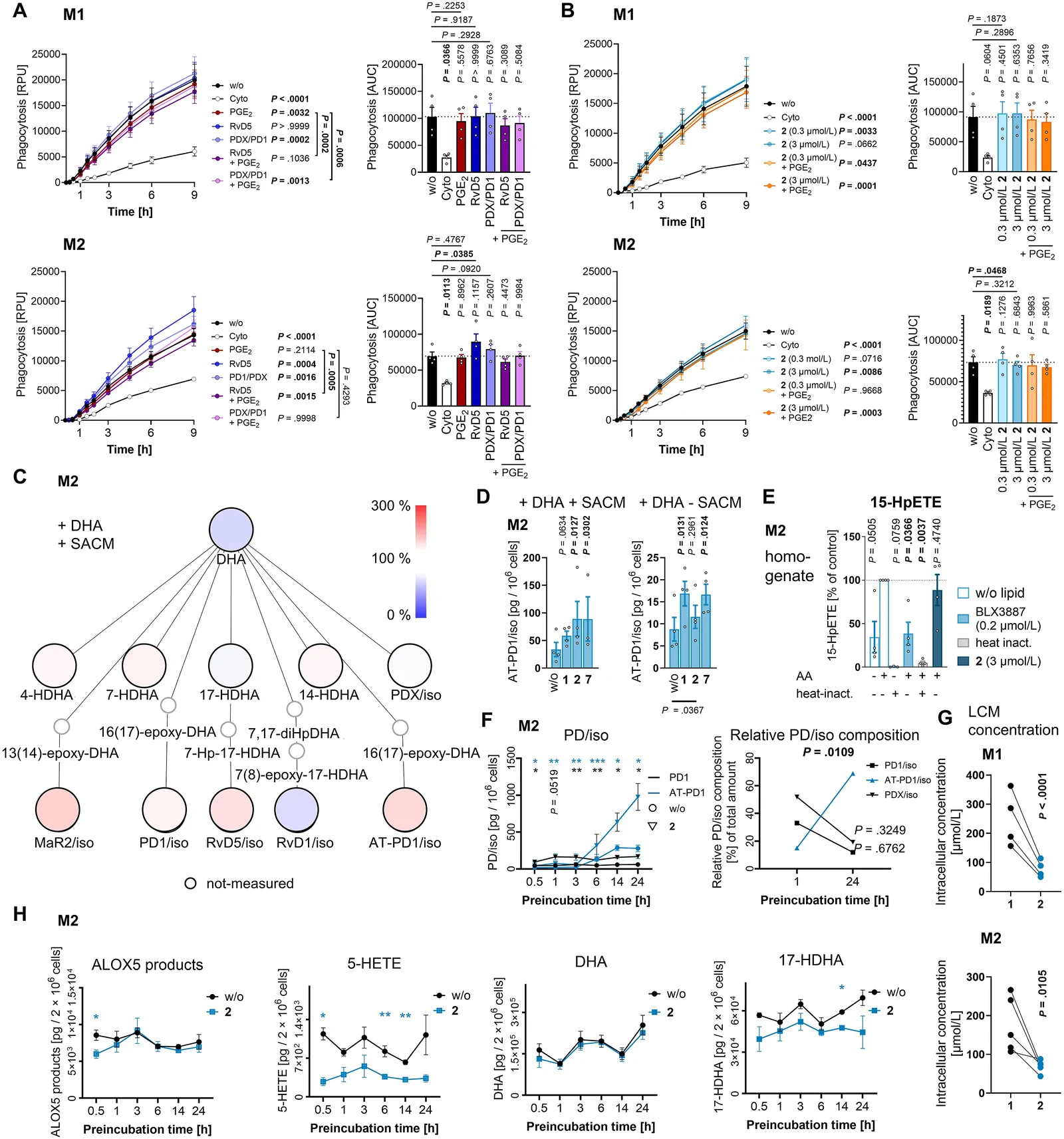
Functional, mechanistic and kinetic studies on the induction of PD formation addressing the impact on phagocytic activity, the role of DHA availability, ALOX15 activation, macrophage activation, and intracellular LCM accumulation. (A, B) Monocyte-derived macrophages (3 × 10^4^) were polarized into M1-like and M2-like subtypes and pre-incubated for 15 min (A) or 3 h (B) with medium alone (‘w/o’) or medium containing cytochalasin D (2 μmol/L), PGE_2_ (10 μmol/L), RvD5 (30 nmol/L), PD1 plus PDX (15 nmol/L, each), LCM **2** (0.3 or 3 μmol/L), or combinations of RvD5, PD1/PDX, or LCM **2** and PGE_2_. Phagocytosis of fluorescent *Staphylococcus aureus* bioparticles was monitored over 9 h; AUC, area under the curve. (C, D) M2-like macrophages (2 × 10^6^) were pre-incubated with vehicle (DMSO, 0.1%, ‘w/o’) or LCMs (at 3 μmol/L) for 15 min and then either supplemented with 20 μmol/L docosahexaenoic acid (DHA) before being stimulated with SACM (1%) for 180 min (‘+ SACM’; C, D) or left untreated after DHA supplementation for a total of 195 min (‘- SACM’; D). (C) Pathway diagram showing percentage changes in DHA-derived lipid mediator formation upon treatment with LCM **2** (3 μmol/L) relative to vehicle. (D) Levels of AT-PD1/iso. (E) Homogenates of 2 × 10^6^ M2-like macrophages were incubated with vehicle (DMSO, 0.1%), **2** (3 μmol/L), or BLX3887 (0.2 μmol/L) for 15 min and then treated with AA (20 μmol/L) for 180 min or left unchallenged. Heat-inactivated homogenates (‘heat-inact.’) (95 °C, 5 min) were used as control. Data for controls are identical to Waltl et al^160^. (F, H) M2-like macrophages (2 × 10^6^ in culture medium) were pre-incubated with LCM **2** (3 μmol/L) or vehicle (DMSO, 0.1%) for 0.5 to 24 h before being stimulated with SACM (1%) for 180 min. (D) Temporal formation of PD1/iso and aspirin-triggered (AT)-PD1/iso for **2**- *vs.* vehicle-treated cells (left panel), and changes in the relative composition of PD species between 1 and 24 h of treatment with **2** (right panel). (G) Intracellular concentrations of **1** and **2** upon exposure to 0.3 μmol/L of the compounds for 20 min, assuming a spherical cell shape (diameter M1: 12 μmol/L; diameter M2: 14 μmol/L). Independent experiments are connected by lines. (H) Time-dependent changes of ALOX5 products, 5-HETE, DHA, and 17-hydroxydocosahexaenoic acid (HDHA) for **2**- *vs.* vehicle-treated cells. Mean (C) or mean ± SEM (A, B, F–H) and single data (A, B, D, E) from *n* = 3–4 independent experiments. *P* values *vs.* vehicle control, as indicated by lines (A, B), DHA ± SACM-treated vehicle control (D), AA-treated vehicle control (E), 1 h of incubation (F, right panel), and **1**-treated cells (G) or ∗*P* < 0.05, ∗∗*P* < 0.01, ∗∗∗*P* < 0.001 *vs.* vehicle treated cells at corresponding time points (F, left panel, H); repeated measures two-way ANOVAs with Geisser Greenhouse correction and Tukey’s *post hoc* tests (A, B; line charts), repeated measures one-way ANOVAs with Geisser Greenhouse correction and Dunnett’s *post hoc* tests (A, B; bar charts), repeated measures one-way ANOVAs with Dunnett’s *post hoc* tests of log data (D) or two-tailed paired Student *t* test (indicated by lines in the bar charts of A and B; E–H).

**Figure 7:**
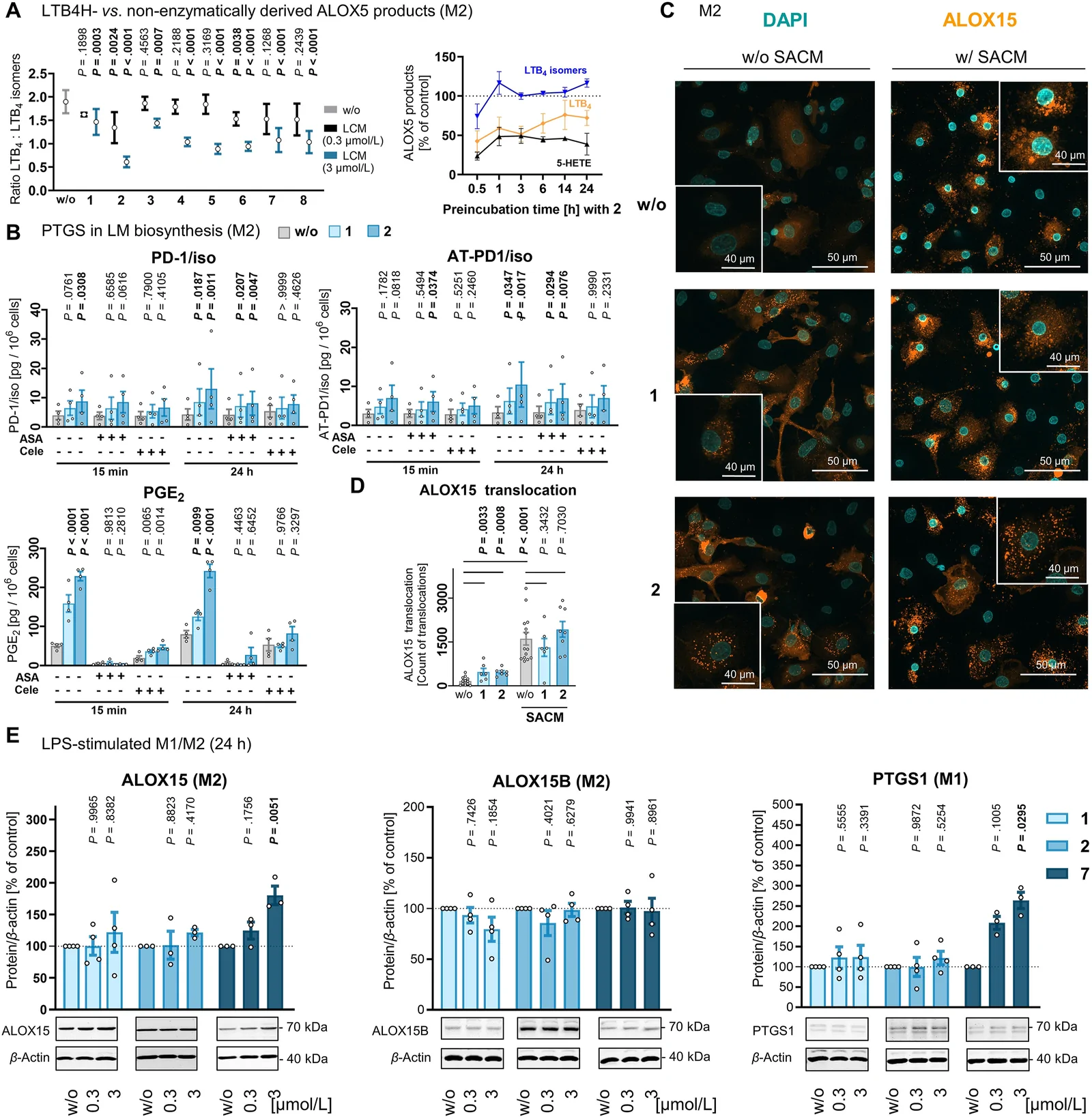
LCMs stimulate the subcellular redistribution of ALOX15. (A) Ratio of leukotriene (LT)B_4_ to LTB_4_ isomers in M2-like macrophages (2 × 10^6^) pre-incubated with vehicle (DMSO, 0.1%, ‘w/o’) or LCMs (at 0.3 or 3 μmol/L) for 15 min and stimulated with SACM (1%) for 180 min (left panel). Time-dependent changes in the level of selected ALOX5 products relative to vehicle (right panel) are shown. (B) Contribution of PTGS isoenzymes to the LCM-induced upregulation of PD/iso and PGE_2_. M2-like macrophages (2 × 10^6^) were pre-incubated with vehicle (DMSO, 0.1%), acetylsalicylic acid (ASA, 500 μmol/L), or celecoxib (‘Cele’, 3 μmol/L) for 15 min or 24 h, then exposed to vehicle (DMSO, 0.1%, ‘w/o’) or LCMs (3 μmol/L) for 15 min, and stimulated with SACM (1%) for 180 min. (C, D) Immunofluorescence staining of ALOX15 in M2-like macrophages pre-incubated with vehicle (DMSO, 0.1%, ‘w/o’) or LCMs (3 μmol/L) for 15 min and then either stimulated with SACM (0.5%, ‘w/SACM’) or left untreated (‘w/o SACM’) for 90 min. The vehicle control is identical to Waltl et al^160^. (C) Representative immunofluorescence images stained for ALOX15 (orange) and nuclei (DAPI, blue) with zoom window. (D) Number of ALOX15-stained particles automatically counted in 60–120 cells/group from three independent experiments. (E) Protein expression of ALOX15, ALOX15B, and PTGS1 in monocyte-derived macrophages (2 × 10^6^) pre-incubated with vehicle (DMSO, 0.1%, ‘w/o’) or LCMs for 15 min and polarized to M1-like macrophages with interferon-*γ* and LPS or to M2-like macrophages with interleukin (IL)-4. Mean ± SEM. (A) and single data (B, D, E) from *n* = 3–4 independent experiments. *P* values *vs.* vehicle-treated cells (A, E), vehicle-treated cells in the presence or the absence of a PTGS inhibitor (B), or as indicated by bars (D); repeated measures one-way ANOVAs with Dunnett’s *post hoc* tests of log data (A, B, E) or two-tailed paired Student *t* test of log data (D).

Lipid mediator production was stopped with 2.5 mL ice-cold methanol containing *d*_8_-5*S*-HETE, *d*_4_-LTB_4_, *d*_5_-LXA_4_, *d*_5_-RvD2, *d*_4_-PGE_2_ (200 pg each) and *d*_8_-AA (2000 pg) as internal standards. For studies supplementing fatty acids (Fig. 6C and D), acetylsalicylic acid or celecoxib (Fig. 7B, Supporting Information Fig. S8), *d*_11_-(±)8(9)-EET (200 pg, Cayman Chemicals), *d*_8_-2-arachidonylglycerol (*d*_8_-2-arachidonoyl glycerol (AG), 2000 pg, Cayman Chemicals), *d*_8_-arachidonoyl ethanolamide (*d*_8_-AEA, 2000 pg, Cayman Chemicals) were added to the number of internal standards. Samples were stored at least for 16 h to allow protein precipitation before being subjected to metabololipidomics analysis.

### 2.9 Treatment of macrophages for quantitative analysis of intracellular LCM concentrations

Macrophages (1 × 10^6^) in PBS pH 7.4 and 1 mmol/L CaCl_2_ were treated with vehicle (DMSO, 0.1%) or LCMs (0.3 μmol/L) for 20 min at 37 °C in 5% CO_2_. Samples were centrifuged (700 × *g*, 5 min, 4 °C) and cell pellets were washed twice with 500 μL PBS pH 7.4 containing 1 mmol/L CaCl_2_. Cells were pelleted (2000 × *g*, 5 min, 4 °C) and snap frozen in liquid nitrogen, and LCM were extracted and quantified by UHPLC–MS/MS as described in section 2.13, 2.15. d-Erythro-sphingosine-*d*_7_-1-phosphate was used as internal standard. Intracellular concentrations of LCMs were calculated assuming a spherical cell shape, uniform intracellular distribution, and a mean cell diameter of 12 μmol/L (M1) and 14 μmol/L (M2), determined using a Vi-Cell XR Cell Viability Analyzer (Beckman Coulter)^60^.

### 2.10 Stimulation of PMNL-platelet co-cultures to induce lipid mediator biosynthesis

Human PMNL (1 × 10^6^) and platelets (2.5 × 10^8^) were pre-incubated in PBS pH 7.4 (1 mL) with vehicle (DMSO, 0.1%) or LCMs for 5 min at room temperature. CaCl_2_ (1 mmol/L) was added and the cells were stimulated with 1% SACM for 180 min at 37 °C. Lipid mediator production was stopped by adding 2 mL of ice-cold methanol containing deuterated internal standards, *i*.*e*., *d*_8_-5*S*-HETE (640.9 pg), *d*_4_-LTB_4_ (672.9 pg), *d*_5_-LXA_4_ (704.9 pg), *d*_5_-RvD2 (753.0 pg), *d*_4_-PGE_2_ (704.9 pg), and *d*_8_-AA (30,447 pg).

### 2.11 Stimulation of human blood for lipid mediator production

The Institute of Transfusion Medicine (University Hospital Jena) collected human venous blood using *S*-Monovettes Lithium-Heparin (Sarstedt AG & Co. KG, Nümbrecht, Germany). Alternatively, residual blood was collected by the Central Institute for Blood Transfusion and Immunology (Tirol Kliniken GmbH, Austria) in Composelect 4F 63 mL CPD/100 mL SAG-M (PQ31555, Fresenius Kabi, Bad Homburg vor der Höhe, Germany) or Quadruple Top-Bottom LCRD2 450 ml CPD/SAGM (LQT610U, Macopharma, Tourcoing, France), using citrate-phosphate-dextrose as anticoagulant. Donors gave informed consent for the use of residual blood. Exclusion criteria for blood donors are specified in Section 2.2. for the preparation of leukocyte concentrates. Heparinized blood was primed with 100 ng/mL LPS for 20 h at 37 °C with shaking before aliquots (1 mL) were pre-incubated with vehicle (DMSO, 0.1%) or LCMs for 15 min. Lipid mediator production was initiated by the Ca^2+^-ionophore A23187 (30 μmol/L, Cayman Chemicals) and samples were incubated for another 10 min at 37 °C. Alternatively, citrated blood (2 mL) was pre-incubated with vehicle (DMSO, 0.1%) or LCMs for 15 min at room temperature and then challenged with 10% SACM for 180 min at 37 °C with shaking. Lipid mediator production was stopped by adding 4 mL of ice-cold methanol supplemented with the internal standards *d*_8_-5*S*-HETE, *d*_4_-LTB_4_, *d*_5_-LXA_4_, *d*_5_-RvD_2_, *d*_4_-PGE_2_ (200 pg each or 640.9 pg, 672.9 pg, 704.9 pg, 753 pg, and 200 pg, respectively) and *d*_8_-AA (2000 pg or 30.4 ng). For SACM-stimulated blood, 200 pg *d*_11_-(±)8(9)-EET, 2000 pg *d*_8_-2-AG, and 2000 pg *d*_8_-AEA were included as additional internal standards. Samples were stored at −20 °C for at least 16 h and then subjected to metabololipidomics sample preparation and analysis. Experiments with blood cells were approved by the ethics committees of the University Hospital Jena or the Medical University Innsbruck (1041/2020).

### 2.12 Zymosan-induced peritonitis in mice

Eight-week-old male CD-1 mice (33–39 g) were obtained from Charles River Laboratories (Calco, Italy). They were provided with standard rodent chow and water and allowed to acclimate for four days in a controlled environment with a 12-h light/12-h dark schedule at a constant temperature of 21 ± 2 °C. Mice were randomly assigned to different experimental groups, and all experiments were performed during the light phase. The experimental procedures adhered to the guidelines outlined in Italian (DL 26/2014) and European (Directive 2010/63/EU) regulations on the ethical use and protection of animals for scientific purposes, followed the ARRIVE guidelines for reporting *in vivo* experiments, and were approved by the Italian Ministry.

CD-1 mice received vehicle (2% DMSO in saline, 0.5 mL), compound **2** (10 mg/kg), or zileuton (10 mg/kg) intraperitoneally (i.p.) followed 30 min later by 0.5 mL zymosan (2 mg/mL in saline, Sigma–Aldrich). Mice were euthanized by CO_2_ inhalation using a gradual-fill method from a compressed gas source (approximately 70% chamber volume per minute), in accordance with the recommendations of the Guide for the Care and Use of Laboratory Animals (8th edition; https://doi.org/10.17226/12910) and the AVMA Guidelines for the Euthanasia of Animals (2020 Edition; Schaumburg, IL: American Veterinary Medical Association, 2020). Animals were continuously monitored, and loss of consciousness and death were confirmed prior to sample collection. Euthanasia was performed 4 and 18 h after zymosan injection to assess the acute and resolution phases of inflammation in plasma and peritoneal exudate. Peritoneal exudate cell counts were determined after trypan blue staining^75^^,^^85^. Plasma and peritoneal exudates (stored at −80 °C) were subjected to metabololipidomics sample preparation and analysis.

### 2.13 Extraction of fatty acids and lipid mediators for targeted metabololipidomics

Aqueous samples from cell-based systems were mixed with methanol and stored at −20 °C for at least 16 h to allow for protein precipitation. Samples were centrifuged (750 × *g*, 10 min, 4 °C), and the supernatant was acidified by adding 7–9 mL water (pH 3.5) or 6 mL PBS (pH 3.5; for LPS-primed and A23187-treated blood). Peritoneal exudates (500 μL) and plasma (100 μL) were combined with 2.5 mL methanol and supplemented with internal deuterated standards, *i*.*e*., *d*_8_-5*S*-HETE, *d*_4_-LTB_4_, *d*_5_-LXA_4_, *d*_5_-RvD_2_, *d*_4_-PGE_2_, *d*_11_-(±)8(9)-EET (200 pg each), *d*_8_-AA, *d*_8_-2-AG, and *d*_8_-AEA (2000 pg each). Proteins were precipitated at −20 °C for ≥16 h, centrifuged (750 × *g*, 10 min, 4 °C) and 8 mL acidified water (pH 3.5) was added. Samples were loaded onto conditioned (6 mL methanol) and equilibrated (2 mL water) solid phase extraction cartridges (Sep-Pak Vac 6 cc 500 mg/6 mL C-18; Waters, Milford, MA, USA), which were washed with water (6 mL) and *n*-hexane (6 mL, 4 °C). Fatty acids and lipid mediators were eluted with methyl formate (6 mL), and the eluates were evaporated to dryness using a TurboVap LV Automated Solvent Evaporation System (Biotage, Uppsala, Sweden). Residues were dissolved in methanol/water (1:1), centrifuged three times (once at 750 × *g*, 10 min, 4 °C or 1200 × *g*, 5 min, 4 °C and once or twice at 21,100 × *g*, 4 °C, 5 min), and the supernatants were analyzed by UHPLC–MS/MS.

### 2.14 Extraction of sphingolipids and LCMs for quantitative analysis by UHPLC–*MS/MS*

Sphingolipids and the LCMs **1** and **2** were extracted from cell pellets, plasma (50 μL), and peritoneal exudates (150 μL) as described^60^^,^^86^^,^^87^. Briefly, sample volumes were adjusted with PBS pH 7.4 to 150 μl aqueous solution before methanol, chloroform, and saline were added sequentially to the samples in a final ratio of 14:34:35:17. The organic layer was dried using an Eppendorf Concentrator Plus system (Eppendorf, Hamburg, Germany) and the residual lipid film was dissolved in methanol, centrifuged twice (21,100 × *g*, 5 min, 4 °C) and subjected to UHPLC–MS/MS analysis. d-Erythro-sphingosine-*d*_7_, *N*-heptadecanoyl-d-erythro-sphingosine, d-glucosyl-*β*-1,1′-*N*-heptadecanoyl-d-erythro-sphingosine, *N*-lauroyl-ceramide-1-phosphate, *N*-heptadecanoyl-d-erythro-sphingosylphosphorylcholine, and d-erythro-sphingosine-*d*_7_-1-phosphate were used as internal standards.

### 2.15 Quantitative analysis of lipid mediators by UHPLC–MS/MS

Lipid mediators and free PUFA were separated on an Acquity UPLC BEH C18 Column (130 Å, 1.7 μm, 2.1 mm × 100 mm, Waters) using an ExionLC AD UHPLC system (Sciex) and detected on a QTRAP 6500^+^ mass spectrometer (Sciex) equipped with an IonDrive Turbo V Ion Source and a TurboIonSpray probe for electrospray ionization^84^. Free fatty acids and oxylipins were analyzed in negative ion mode and endocannabinoids in positive ion mode using scheduled multiple reaction monitoring (MRM) and polarity switching. Chromatographic settings and source parameters were adjusted as previously described^84^. MRM transitions, retention times, and analyte-specific parameters are detailed in Table 1, Table 2.

**Table 1:** Quantitative analysis of lipid mediators by MRM in the negative ion mode.

Q1 [*m*/*z*]	Q3 [*m*/*z*]	RT^a^ [min]	Species	Window [s]	DP^b^ [V]	EP^c^ [V]	CE^d^ [eV]	CXP^e^ [V]
349.2	113.2	6.0	15-keto-PGE_2_	90	−80	−10	−16	−15
369.3	169.1	6.3	TXB_2_	130	−80	−10	−22	−15
355.3	193.2	6.6	d4-PGE_2_	60	−80	−10	−25	−16
351.2	271.0	6.6	PGE_2_	90	−120	−10	−20	−13
351.3	189.1	6.8	PGD_2_	90	−120	−10	−20	−13
380.3	141.2	6.9	d5-RvD2	60	−80	−10	−23	−14
353.2	317.4	6.9	PGE_1_/PGD_1_	120	−90	−10	−18	−15
375.2	175.1	7.0	RvD2	90	−80	−10	−21	−13
353.3	193.1	7.0	PGF_2*α*_	60	−80	−10	−34	−11
356.3	115.2	7.2	d5-LXA4	60	−80	−10	−19	−14
375.2	215.1	7.4	RvD1	90	−80	−10	−40	−13
351.2	235.1	7.4	LXA4	90	−80	−10	−20	−13
333.3	115.1	9.0	5.15-diHEPE	90	−80	−10	−22	−13
361.5	143.1	9.6	RvD5_n-3DPA_	60	−60	−10	−20	−10
339.3	197.2	9.7	d4-LTB_4_	60	−80	−10	−22	−13
359.2	153.1	9.8	AT-PD1	120	−80	−10	−21	−9
335.2	201.0	9.9	5.15-diHETE	90	−80	−10	−22	−13
359.2	221.0	10.0	MaR2	90	−80	−10	−20	−12
335.2	195.1	10.0	LTB_4_ Isomers	120	−80	−10	−22	−13
359.2	199.1	10.1	RvD5	60	−80	−10	−21	−13
359.2	153.1	10.4	PDX	120	−80	−10	−21	−9
337.2	207.1	10.4	14.15-DHET	60	−60	−5	−20	−10
335.2	195.1	10.5	LTB_4_	120	−80	−10	−22	−13
359.2	153.1	10.6	PD1	120	−80	−10	−21	−9
337.2	167.1	10.7	11.12-DHET	60	−30	−5	−30	−15
279	163.0	10.7	12-HHT	90	−30	−10	−30	−13
337.2	127.1	10.9	8.9-DHET	60	−60	−5	−30	−15
317.2	219.1	11.0	15-HEPE	60	−80	−10	−18	−12
317.2	179.1	11.2	12-HEPE	60	−80	−10	−19	−12
317.2	167.1	11.2	11-HEPE	60	−80	−10	−19	−12
317.2	215.1	11.4	17.18-EpETE	60	−40	−5	−15	−10
337.2	145.0	11.4	5.6-DHET	60	−70	−5	−20	−10
317.2	115.1	11.5	5-HEPE	60	−80	−10	−18	−12
295.2	171.0	11.5	9-HODE	90	−60	−10	−19	−13
295.2	195.0	11.5	13-HODE	90	−60	−10	−25	−13
317.2	259.1	11.6	18-HEPE	90	−80	−10	−16	−23
343.2	193.1	11.7	13-HDHA	90	−80	−10	−17	−14
319.2	219.1	11.8	15-HETE	60	−80	−10	−19	−12
343.2	245.1	11.9	17-HDHA	60	−80	−10	−17	−14
335.2	115.1	11.9	5*S*,6*R*-diHETE	80	−80	−10	−20	−13
317.2	155.0	11.9	8.9-EpETE	90	−10	−5	−20	−10
319.2	167.1	11.9	11-HETE	60	−80	−10	−21	−12
343.2	205.1	12.0	14-HDHA	60	−80	−10	−17	−14
319.2	179.1	12.0	12-HETE	60	−80	−10	−21	−12
345.2	247.1	12.2	17-HDPA	90	−80	−10	−17	−14
345.2	207.1	12.2	14-HDPA	90	−80	−10	−17	−14
319.2	219.2	12.2	14.15-EET	60	−60	−10	−20	−10
343.2	141.1	12.2	7-HDHA	60	−80	−10	−18	−15
327.3	116.1	12.3	*d*_8_-5*S*-HETE	60	−80	−10	−17	−10
343.2	241.1	12.3	19.20-EpDPA	70	−40	−10	−20	−13
319.2	115.1	12.4	5-HETE	60	−80	−10	−21	−12
343.2	233.2	12.4	16.17-EpDPA	70	−20	−5	−15	−20
317.2	207.1	12.4	14.15-EpETE	60	−30	−10	−20	−10
319.2	167.2	12.5	11.12-EET	60	−40	−10	−20	−10
317.2	167.1	12.5	11.12-EpETE	90	−40	−10	−15	−15
330.2	155.2	12.6	*d*_11_-8.9-EET	60	−30	−5	−20	−10
343.2	153.2	12.6	10.11-EpDPA	70	−40	−10	−20	−13
343.2	193.0	12.6	13.14-EpDPA	70	−40	−10	−20	−13
343.2	101.1	12.6	4-HDHA	60	−80	−10	−17	−15
319.2	155.0	12.6	8-HETE	90	−50	−10	−18	−13
319.2	167.0	12.7	8.9-EET	60	−40	−5	−20	−10
343.2	189.0	12.8	7.8-EpDPA	70	−50	−5	−20	−5
319.2	191.1	12.9	5.6-EET	60	−50	−5	−20	−15
345.2	143.1	13.3	7-HDPA	90	−80	−10	−18	−15
301.3	257.1	13.9	EPA	90	−100	−10	−16	−18
311.3	267.1	14.6	*d*_8_-AA	90	−100	−10	−16	−18
327.3	283.1	14.6	DHA	90	−100	−10	−16	−18
329.3	285.1	15.2	DPA	90	−100	−10	−16	−18
303.3	259.1	15.4	AA	90	−100	−10	−16	−18

AA, arachidonic acid; DHA, docosahexaenoic acid; DHET, dihydroxyeicosatrienoic acid; (di)HEPE; (di)hydroxyeicosapentaenoic acid; (di)HETE, (di)hydroxyeicosatetranoic acid; DPA, docosapentaenoic acid; EET, epoxyeicosatrienoic acid; EPA, eicosapentaenoic acid; EpDPA, epoxydocosapentanoic acid; EpETE, epoxyeicosatetraenoic acid; HDHA, hydroxydocosahexanoic acid; HDPA, hydroxydocosapentanoic acid; HHT, hydroxyheptadecatrienoic acid; HODE, hydroxyoctadecadienoic acid; LT, leukotriene; LX, lipoxin; MaR, maresin; PD, protectin; PG, prostaglandin; Rv, resolvin; TX, thromboxane.

a Retention time.

b Declustering potential.

c Entrance potential.

d Collision energy.

e Collision cell exit potential.

**Table 2:** Quantitative analysis of lipid mediators by MRM in the positive ion mode.

Q1 [*m/z*]	Q3 [*m/z*]	RT^a^ [min]	Species	Window [s]	DP^b^ [V]	EP^c^ [V]	CE^d^ [eV]	CXP^e^ [V]
387.2	294.2	13.57	*d*_8_-2-AG	90	50	10	20	20
356.3	63.2	13.39	*d*_8_-AEA	90	30	10	20	10
379.2	287.3	13.83	1-AG	90	30	10	20	15
379.1	287.4	13.62	2-AG	90	40	10	20	15
348.2	287.4	13.35	AEA	90	20	10	20	15
300.3	62.0	13.86	PEA	90	30	10	20	10
326.5	62.1	14.25	OEA	90	30	10	20	40

AEA, arachidonoylethanolamide; AG, arachidonoylglycerol; OEA, oleoylethanolamide; PEA, palmitoylethanolamide.

a Retention time.

b Declustering potential.

c Entrance potential.

d Collision energy.

e Collision cell exit potential.

Alternatively, lipid mediators and PUFA were analyzed using an Acquity ultraperformance liquid chromatography system (Waters) that was coupled to a QTRAP 5500 mass spectrometer (Sciex, Framingham, MA, USA) equipped with a Turbo V Ion Source and a TurboIonSpray probe for electrospray ionization using previously published settings (Fig. 5D, Supporting Information Fig. S1)^13^^,^^38^^,^^55^^,^^60^.

Mass spectra were acquired and processed using Analyst 1.7.1 or 1.6.3 (Sciex). Absolute concentrations of lipid mediators and free fatty acids were calculated from 11- or 15-point hyperbolic or linear standard curves and normalized to subclass-specific deuterated internal standards and either the cell number or volume. Standards used for external calibration: AA, *d*_11_-8,9-EET, *d*_8_-AEA, PGD_2_, PGE_2_, PGF_2*α*_, PGA_2_, PGJ_2_, 15-desoxy-delta-12,14-PGJ_2_, 15-keto-PGE_2_, thromboxane (TX)B_2_, LTB_4_, 20-OH-LTB_4_, aspirin-triggered (AT)-LXA_4_, 5,15-dihydroxyeicosatetraenoic acid (diHETE), 5*S*,6*R*-diHETE, 5-HETE, 11-HETE, 12-HETE, 15-HETE, eicosapentaenoic acid (EPA), 5-hydroxyeicosapentaenoic acid (HEPE), 11-HEPE, 12-HEPE, 15-HEPE, 18-HEPE, DHA, RvD3, 17-hydroxydocosahexaenoic acid (HDHA), 14-HDHA, 10-HDHA, 7-HDHA, 4-HDHA, MaR1, MaR2, RvD5, docosapentaenoic acid (DPA), PGE_1_, 5,6-EET, 8,9-EET, 11,12-EET, 14,15-EET, 5,6-dihydroxyeicosatrienoic acid (DHET), 8,9-DHET, 11,12-DHET, 14,15-DHET, RvD1, RvD2, RvD4, RvE4, RvD5_n3 DPA_, AT-PD1, PD1, PDX, 7,8-epoxydocosapentaenoic acid (EpDPA), 10,11-EpDPA, 13,14-EpDPA, 16,17-EpDPA, 19,20-EpDPA, 8,9-epoxyeicosatetraenoic acid (EpETE), 11,12-EpETE, 14,15-EpETE, 17,18-EpETE, *d*_8_-2-AG, 1-AG, AEA, oleoyl ethanolamide (OEA), palmitoyl ethanolamide (PEA), 13-hydroxyoctadecadienoic acid (HODE), 9-HODE (Cayman Chemicals), and the internal standards *d*_4_-PGE_2_, *d*_5_-RvD_2_, *d*_8_-5-HETE, *d*_4_-LTB_4_, *d*_5_-LXA_4_, *d*_8_-AA. When AA and DHA signals were not covered by the dynamic range of the mass spectrometer, ^13^C-isotope peaks ([M-H+1]^–^) were analyzed.

### 2.16 Quantitative analysis of sphingolipids by UHPLC–MS/MS

Sphingosine (So), sphinganine (Sa), (dihydro)ceramide ([dh]Cer), (dihydro)sphingomyelin ([dh]SM), hexosylceramide (HexCer), and ceramide-1-phosphate (C1P) were separated at 45 °C and a flow rate of 0.75 mL/min on an Acquity UPLC BEH C8 Column (130 Å, 1.7 μm, 2.1 mm × 100 mm, Waters) using an ExionLC AD UHPLC system (Sciex). The gradient of mobile phase A (acetonitrile/water, 95/5 and 2 mmol/L ammonium acetate) and mobile phase B (acetonitrile/water, 10/90 and 2 mmol/L ammonium acetate) was ramped from 75% to 85% A within 5 min, increased to 100% A within another 2 min, and maintained isocratically for 13 min. The UHPLC system was coupled to a QTRAP 6500^+^ mass spectrometer (Sciex) equipped with an electrospray ionization source. Sphingolipids were analyzed by scheduled MRM in the positive ion mode based on transitions from [M+H]^+^ to [M + H-H_2_O]^+^ (So, Sa, dhCer), *m/z* = 184.1 ([dh]SM), and *m/z* = 264.4 (Cer, HexCer, C1P). Source and compound-specific parameters are listed in Table 3.

**Table 3:** Source and compound parameters for the quantitative analysis of sphingolipids.

Positive ion mode		So/Sa	[dh]Cer	HexCer	C1P	[dh]SM	S1P
Curtain gas	[psi]	40	40	40	40	40	40
Ion spray voltage	[V]	5000	5000	5000	5000	5000	4500
Heated capillary temperature	[°C]	500	500	500	500	500	550
Sheath gas	[psi]	40	40	40	40	40	60
Auxiliary gas	[psi]	40	40	40	40	40	30
Deculstering potential	[V]	30	30	40	30	40	40
Entrance potential	[V]	10	10	5	10	10	10
Collision energy	[eV]	20	40	50	40	40	20
Collision gas		Medium	Medium	Medium	Medium	Medium	Low
Collision cell exit potential	[V]	25	20	20	5	10	20

C1P, ceramide-1-phosphate; [dh]Cer, [dihydro]ceramide; HexCer, hexosylceramide; S1P, sphingosine-1-phosphate; Sa, sphinganine; [dh]SM, [dihydro]sphingomyelin; So, sphingosine.

The analysis of sphingosine-1-phosphate (S1P) was performed on an ExionLC AD UHPLC system coupled to a QTRAP 6500^+^ mass spectrometer (Sciex). S1P was isocratically eluted at a flow rate of 0.55 using water/acetonitrile/isopropanol (32/20/48) and 0.1% formic acid as the mobile phase on an Acquity UPLC CSH C18 column (130 Å, 1.7 μm, 2.1 mm × 50 mm, Waters) at 55 °C mL/min. Fragmentation of [S1P + H]^+^ (*m/z* 380.3) to [S1P + H-H_3_PO_4_-H_2_O]^+^ (*m/z* 264.2) was monitored by MRM in positive ion mode. Table 3 shows the mass spectrometric settings used.

Mass spectra were acquired and processed using Analyst 1.7.1 (Sciex) and Analyst 1.6.3 (Sciex). Absolute levels of sphingolipid species were determined by 11-point calibration and normalized to subclass-specific deuterated internal standards and the sample volume. Subclass-specific external standards used (Sigma–Aldrich): d-erythro-sphingosine-*d*_7_, *N*-heptadecanoyl-d-erythro-sphingosine, d-glucosyl-*β*-1,1′-*N*-heptadecanoyl-d-erythro-sphingosine, *N*-lauroyl-ceramide-1-phosphate, *N*-heptadecanoyl-d-erythro-sphingosylphosphorylcholine, and d-erythro-sphingosine-*d*_7_-1-phosphate.

### 2.17 Quantitative analysis of LCMs by UHPLC–MS/MS

The chromatographic separation of the LCMs **1** and **2** was performed at 45 °C on an Acquity UPLC CSH C18 column (130 Å, 1.7 μmol/L, 2.1 mm × 50 mm) using an ExionLC AD UHPLC system (Sciex). The system was operated at a flow rate of 0.8 mL/min using acetonitrile/water (10/90) with 0.07% formic acid as mobile phase A and 100% acetonitrile with 0.07% formic acid as mobile phase B. The gradient was ramped from 50% to 100% mobile phase B within 4.5 min, followed by isocratic elution for 1 min. Eluted LCMs were detected by MRM in the negative ion mode using a QTRAP 6500^+^ mass spectrometer (Sciex) equipped with an electrospray ionization source. Transitions from *m/z* 459.4 to *m/z* 163.0 (compound **1**, retention time: 3.51 min) and from *m/z* 453.3 to *m/z* 163.0 (compound **2**, retention time: 2.71 min) were monitored. The curtain gas was set to 40 psi, the collision gas to medium, the ion spray voltage to −4500 V, the heated capillary temperature to 450 °C, the sheath gas pressure to 60 psi, the auxiliary gas pressure to 30 psi, the declustering potential to −80 V (**1**) or −110 V (**2**), the entrance potential to −10 V, the collision energy to −46 eV (**1**) or −40 eV (**2**), and the collision cell exit potential to −10 V. Data were acquired and processed using Analyst 1.7.1 (Sciex) and Analyst 1.6.3 (Sciex). Absolute LCM concentrations were calculated using external 15-point calibration curves for **1** and **2**, respectively, and then normalized to the internal standard d-erythro-sphingosine-*d*_7_-1-phosphate (analyzed using the chromatographic and mass spectrometric settings described in section 2.16) and cell number.

### 2.18 Determination of cell viability and ferroptosis susceptibility

The effect of LCMs on cell viability was estimated by measuring the conversion of 3-[4,5-dimethylthiazol-2-yl]-2,5-diphenyltetrazolium bromide (MTT; Sigma–Aldrich) to purple formazan by cellular dehydrogenases as described^39^. Briefly, monocytes (1 × 10^5^ cells), M1-like macrophages (1.5 × 10^5^ cells), or M2-like macrophages (1.5 × 10^5^ cells) in 100 μL of RPMI 1640 medium containing 5% (for monocytes) or 10% FCS (for M1 and M2-like macrophages), 100 U/mL penicillin and 100 μg/mL streptomycin were treated with vehicle (DMSO, 0.1%) or LCMs for 48 h at 37 °C in 5% CO_2_. Alternatively, human HepaRG hepatocytes (1 × 10^4^/well) in William’s E medium (Sigma–Aldrich) supplemented with 10% FCS (Sigma–Aldrich, #0001662318), 2 mmol/L l-glutamine (Sigma–Aldrich), 5 μg/mL human insulin (Sigma–Aldrich) and 50 μmol/L hydrocortisone (Cayman Chemicals) were seeded in 96-well plates and incubated for 24 h at 37 °C and 5% CO_2_. The cells were then exposed to vehicle (DMSO, 0.5%), (1*S*,3*R*)-RSL3 (0.3 μmol/L, Cayman Chemicals), or a combination of RSL3 with *α*-tocopherol (Cayman Chemicals) or LCMs for 48 h. 3-(4,5-Dimethylthiazol-2-yl)-2,5-diphenyltetrazolium bromide (MTT; 20 μL of a 5 mg/mL solution in PBS pH 7.4) was added to each well, and the cells were incubated for an additional 3 h. The cells were lysed, and the purple formazan crystals were solubilized by adding 100 μL of 10% sodium dodecyl sulfate (SDS) in 20 mmol/L HCl. After shaking in the dark for 16 to 20 h, the absorbance was measured at 570 nm using a Multiskan Spectrum microplate reader (ThermoFisher Scientific; monocytes and M1-like and M2-like macrophages) or a Hidex Sense Beta Microplate Reader (Hidex Oy, Turku, Finland; HepaRG cells). The pan-kinase inhibitor staurosporine (10 μmol/L, Sigma–Aldrich) was used as control.

### 2.19 Phagocytotic activity

Monocyte-derived macrophages (3 × 10^4^ cells) were seeded in a 96-well plate in 90 μL of RPMI 1640 medium supplemented with 10% FCS and 2 mmol/L l-glutamine. After 2 h of incubation (5% CO_2_, 37 °C), 10 μL of medium containing either LPS and interferon-*γ* (final concentrations: 100 ng/mL LPS and 20 ng/mL interferon-*γ*) or IL-4 (final concentration: 20 ng/mL) was added to generate M1- or M2-like macrophages, respectively^88^. M1-polarized cells were used after 24 h, and M2-polarized cells after 48 h, to study phagocytosis. The medium was removed, and 75 μL of RPMI 1640 medium without phenol red (Sigma–Aldrich) was added, either alone (control) or supplemented with lipid mediators and/or LCM **2**. Cells were pre-incubated at 5% CO_2_ and 37 °C for 15 min when lipid mediators were investigated, or for 180 min when LCM 2 was the focus. Subsequently, 25 μL of PBS pH 7.4 containing human serum (Sigma–Aldrich, #P35367; final concentration: 5%) and pHrodo Green *S. aureus* BioParticles (Invitrogen, #P35367; final amount: 5 μg/well) were added. The bioparticles had been opsonized for 30 min (5% CO_2_, 37 °C) with 20% human serum prior to use. Phagocytosis was assessed using a temperature-controlled (37 °C) NOVOstar microplate reader (BMG Labtech GmbH) at the indicated time points over 9 h. Fluorescence measurements for pHrodo Green were taken at an excitation wavelength of 485 nm and an emission wavelength of 520 nm. As a positive control, a well containing opsonized *Staphylococcus aureus* bioparticles without cells was acidified to pH 5 using HCl. A blank control consisted of 75 μL of RPMI 1640 medium without phenol red, 25 μL of PBS (pH 7.4), 5 μg of pHrodo Green *S. aureus* BioParticles, and 5% human serum, and was used for background subtraction. Relative phagocytosis units (RPU) were calculated by subtracting the blank-corrected fluorescence intensities at *t* = 0 h from the blank-corrected fluorescence intensities at the time point of measurement.

### 2.20 Determination of cell-free ALOX15/ALOX15B activity

M2-like macrophages (3.3 × 10^7^–8.6 × 10^7^) were homogenized by sonication (3 × 15 s on ice, 125 W, power set to 35%) in 16.5–43 mL PBS pH 7.4 supplemented with 1 mmol/L EDTA. Aliquots of 1 mL were pre-incubated with vehicle (DMSO, 0.1%) or compound **2** for 15 min on ice, and ALOX15 product formation was initiated by adding 2 mmol/L CaCl_2_ and 20 μmol/L AA. After 15 min at 37 °C, the reaction was stopped by the addition of 1 mL ice-cold methanol containing *d*_4_-PGE_2_, *d*_5_-RvD_2_, *d*_8_-5-HETE, *d*_4_-LTB_4_, *d*_5_-LXA_4_-*d*_5_, *d*_8_-AA, *d*_11_-8,9-EET, and *d*_8_-AEA (Cayman Chemicals) as internal standards. 15-HpETE and 15-HETE were extracted and analyzed by UHPLC–MS/MS as described in Sections 2.13, 2.15.

### 2.21 Determination of PTGS1/2 activity

PTGS1 and PTGS2 activities were determined as described^89^. In brief, purified bovine PTGS1 (Cayman Chemical, 50 units) or human recombinant PTGS2 (Cayman Chemicals, 20 units) was added to 100 mmol/L Tris pH 8 containing 5 mmol/L glutathione, 5 μmol/L hemoglobin, and 100 μmol/L EDTA. After preincubation with vehicle (DMSO, 0.1%) or LCMs for 5 min at room temperature, the mixtures were prewarmed at 37 °C for 1 min and the formation of PTGS derived 12(*S*)-hydroxy-5-*cis*-8,10-*trans*-heptadecatrienoic acid (12-HHT) was initiated by the addition of AA (PTGS1: 5 μmol/L; PTGS2: 2 μmol/L). The reaction was stopped after 5 min with ice-cold methanol containing PGB_1_ (200 ng, Cayman Chemicals) as internal standard. The analytes were extracted by solid phase extraction using Sep-Pak C18 35 cc Vac Cartridges (Waters), separated by reversed phase HPLC on a Nova-Pak C18 Radial-Pak column (4 μm, 5 mm × 100 mm, Waters), and detected at 235 nm (12-HHT) and 280 nm (PGB_1_). The PTGS1/2 inhibitor indomethacin (10 μmol/L, Sigma–Aldrich) and the selective PTGS2 inhibitor celecoxib (3 μmol/L, Cayman Chemicals) were used as controls.

### 2.22 Determination of PTGS1 and 12-lipoxygenase (ALOX12) activity in human platelets

PTGS1 and ALOX12 product formation was determined in freshly isolated human platelets as previously reported^90^. Briefly, platelets (1 × 10^8^) were pre-incubated with vehicle (DMSO, 0.1%) or LCMs in PBS pH 7.4 and 1 mg/mL glucose for 4.5 min at room temperature. CaCl_2_ (1 mmol/L) was added and the samples were incubated for another 30 s at room temperature and then pre-warmed at 37 °C for 1 min. 12-HHT (PTGS1 product) and 12-HETE (ALOX12 product) formation was initiated by the addition of 5 μmol/L AA and terminated after 5 min by adding ice-cold methanol. PGB_1_ (200 ng, Cayman Chemicals) was used as internal standard. 12-HHT and 12-HETE were extracted by solid phase extraction using Sep-Pak C18 35 cc Vac Cartridges (Waters), separated by reversed phase HPLC on a Nova-Pak C18 Radial-Pak column (4 μm, 5 mm × 100 mm, Waters), and detected at 235 nm (12-HHT and 12-HETE) and 280 nm (PGB_1_).

### 2.23 Determination of soluble expoxide hydrolase (EPHX2, *sEH) activity*

EPHX2 was expressed in Sf9 insect cells and purified by benzylthio-sepharose affinity chromatography, and its activity was measured as described^91^. In brief, isolated EPHX2 (60 ng) was diluted in 25 mmol/L Tris HCl (pH 7) containing 0.1 mg/mL BSA and pre-incubated with vehicle (DMSO, 0.1%) or LCMs for 10 min at room temperature. The EPHX2 substrate 3-phenyl-cyano(6-methoxy-2-naphthalenyl)methyl ester-2-oxiraneacetic acid (PHOME, 20 μmol/L, Cayman Chemicals) was added and converted by EPHX2 to the fluorescent product 6-methoxy-naphthaldehyde. After 60 min in the dark, the reaction was stopped by adding 200 mmol/L ZnSO_4_, and the fluorescence (Ex/Em = 330 nm/465 nm) was measured using a NOVOstar microplate reader (BMG Labtechnologies, Offenburg, Germany). The selective EPHX2 inhibitor AUDA (1 μmol/L) was used as reference.

### 2.24 ALOX15 translocation in M2-like macrophages analyzed by fluorescence microscopy

The subcellular localization of ALOX15 was visualized in SACM-stimulated M2-like macrophages according to a previously published protocol^34^^,^^35^. Briefly, M2-like macrophages (8 × 10^5^) suspended in RPMI 1640 medium, 10% FCS, 2 mmol/L l-glutamine, 100 U/mL penicillin, and 100 μg/mL streptomycin were seeded onto glass coverslips (Thermo Fisher Scientific) coated with polylysine (Sigma–Aldrich) for 30 min. After incubation for 30 min at 37 °C in 5% CO_2_, cells were treated with vehicle (DMSO, 0.1%) or LCMs (3 μmol/L) for 15 min followed by stimulation with 0.5% SACM for 90 min. Alternatively, cells were incubated with vehicle or LCMs for 105 min without further stimulation. Cells were fixed with 4% paraformaldehyde (Sigma–Aldrich) in PBS pH 7.4 for 20 min at room temperature, washed three times with PBS pH 7.4, 1 mmol/L CaCl_2_, and 1 mmol/L MgCl_2_, and permeabilized with acetone for 3 min on ice followed by treatment with aqueous 0.1% Triton X-100 for 10 min at room temperature. After three washes with PBS pH 7.4, 1 mmol/L CaCl_2_, and 1 mmol/L MgCl_2_, samples were blocked with 10% normal goat serum (ThermoFisher Scientific) in PBS pH 7.4 with 0.1% sodium azide for 30 min at room temperature and incubated with primary mouse monoclonal anti-ALOX15 antibody (1:100, #ab119774, Abcam, Cambridge, UK) overnight at 4 °C. The coverslips were washed thrice with PBS pH 7.4, 1 mmol/L CaCl_2_, and 1 mmol/L MgCl_2_ and stained with Alexa Fluor 555-conjugated goat anti-mouse IgG (1:500, #A21422, Invitrogen, Carlsbad, CA, USA) for 30 min at room temperature. After being washed three times with PBS pH 7.4, 1 mmol/L CaCl_2_, and 1 mmol/L MgCl_2_, coverslips were mounted onto glass slides using ProLong Gold Antifade Mountant with 4′,6-diamidino-2-phenylindol (DAPI, ThermoFisher Scientific). Immunofluorescence was analyzed using an AxioObserver Z1 microscope (Carl Zeiss, Jena, Germany) operated by the Zen 2.6 software (Carl Zeiss) and equipped with a Plan-Apochromat 40 ×/1.4 Oil DIC M27 objective (Carl Zeiss) and an Axiocam 702 (Carl Zeiss) for image acquisition. The exposure time was kept constant for all images and Z-stacking was applied (9–11 slices, *Z* = 0.9–1.1 μmol/L). Further analysis of the czi files and data export were carried out using ImageJ 1.53t (Wayne Rasband and contributors, National Institutes of Health, Bethesda, MD, USA) and ZEN 3.7 (Carl Zeiss, Jena, Germany). For merged images, the DAPI channel was pseudocolored light petroleum blue and the Alexa 555 channel was psuedocolored red. To quantify the ALOX15 translocation to particulate locales, the threshold for background separation was set the same for all images (ALOX15 translocation: 4000–65535; DAPI: 200-65535) and particles were counted with circularity 0–1 and size (micron^2^) set either to 0–infinity (ALOX15) or 10–infinity (DAPI/cell count).

### 2.25 Flow cytometric analysis of monocyte-derived macrophages

Macrophage polarization into M1-like and M2-like phenotypes was monitored by flow cytometry as previously described^13^. In brief, monocyte-derived macrophages (2 × 10^6^) were treated with vehicle (DMSO, 0.1%) or LCMs in RPMI 1640 medium containing 10% fetal calf serum (FCS, Sigma–Aldrich), 100 U/mL penicillin, and 100 μg/mL streptomycin at 37 °C and 5% CO_2_ for 15 min and then polarized to M1-like or M2-like phenotypes for 48 h as described above. The culture medium was replaced with PBS pH 7.4, 5 mmol/L EDTA, 0.1% sodium azide, and 0.5% bovine serum albumin to detach the cells, which were recovered by centrifugation (400 × *g*, 5 min, 4 °C) after 20 min. Cells with compromised membranes were stained with Zombie Aqua Fixable Viability Kit (Biolegend, San Diego, CA, USA) for 5 min at 4 °C according to the manufacturer’s instructions. Non-specific antibody binding was blocked with 1% mouse serum (Thermo Fisher Scientific) in PBS pH 7.4 for 10 min at 4 °C, followed by staining of macrophage surface markers with fluorochrome-labeled antibodies for 20 min at 4 °C. Antibodies used: APC-H7 anti-human CD80 (clone L307.4, undiluted, #561134, BD Biosciences, San Jose, CA, USA), PE-Cy7 anti-human CD54 (clone HA58, undiluted, #BLD-353116, Biolegend), PE anti-human CD163 (clone GHI/61, undiluted, #556018, BD Biosciences), and APC anti-human CD206 (clone 19.2, undiluted, #550889, BD Biosciences). Stained cells were measured using an LSRFortessa cell analyzer (BD Biosciences), and data were analyzed using FlowJo X software (BD Biosciences). The gating strategy is outlined in Supporting Information Fig. S14.

### 2.26 Sample preparation, SDS-Page and Western blotting

Protein expression of enzymes involved in lipid mediator biosynthesis was analyzed in M1 and M2-like macrophages exposed to vehicle (DMSO, 0.1%) or LCMs for 48 h and in monocytes treated with 100 ng/mL LPS and either vehicle (DMSO, 0.1%) or LCMs for 24 h by Western blotting as described^13^. Briefly, pellets of 2 × 10^6^ macrophages or 3 × 10^6^ monocytes were lysed in 1% (*v*/*v*) NP-40 (AppliChem, Darmstadt, Germany), 1 mmol/L sodium orthovanadate (AppliChem), 10 mmol/L sodium fluoride (AppliChem), 5 mmol/L sodium pyrophosphate (Sigma–Aldrich), 25 mmol/L *β*-glycerophosphate (Sigma–Aldrich), 5 mmol/L EDTA (AppliChem), 25 μmol/L leupeptin (Sigma–Aldrich), 3 μmol/L soybean trypsin inhibitor (Sigma–Aldrich), and 1 mmol/L phenylmethanesulfonyl fluoride (Sigma–Aldrich). After 5 min on ice, cells were scraped, lysates centrifuged (21,000×*g*, 5 min, 4 °C), and the protein concentration of the supernatant was determined using a DC Protein Assay kit (Bio-Rad Laboratories GmbH, Munich, Germany). Aliquots were adjusted to a concentration of 1 mg/mL total protein, mixed with 1 × Laemmli buffer (125 mmol/L Tris-HCl pH 6.5, 25% (*w*/*v*) sucrose, 5% (*w*/*v*) SDS, 0.25% (*w*/*v*) bromophenol blue, and 10% (*v*/*v*) *β*-mercaptoethanol, and heated at 95 °C for 5 min. Samples (corresponding to 10 μg of total protein) were resolved on 10% polyacrylamide gels, and proteins were transferred onto Amersham Protran NC 0.45 nitrocellulose membranes (GE Healthcare). The membranes were blocked with 5% (*w*/*v*) BSA for 1 h at room temperature, washed, and then incubated overnight at 4 °C with the following primary antibodies: rabbit polyclonal anti-cPLA_2*α*_ (1:200, #2832, Cell Signaling, Danvers, MA, USA); mouse monoclonal anti-ALOX5 (1:1000, #610694, BD Biosciences); mouse monoclonal anti-ALOX15 (1:200, ab119774, Abcam, Cambridge, UK); rabbit polyclonal anti-ALOX15B (1:200, ab23691, Abcam); rabbit monoclonal anti-PTGS1 (1:1000, #4841, Cell Signaling); rabbit monoclonal anti-PTGS2 (1:500, #12282, Cell Signaling); mouse monoclonal anti-*β*-actin (1:1000, #3700, Cell Signaling). Immunoreactive bands were stained with IRDye 800CW goat anti-rabbit IgG (1:15,000, #926-32211, LI-COR Biosciences, Lincoln, Nebraska) and IRDye 680LT goat anti-mouse IgG (1:40,000, #926-68020, LI-COR Biosciences) for 60 min at room temperature and visualized with an Odyssey infrared imager (LI-COR Biosciences, Lincoln, NE, USA). Data from densitometric analysis were background corrected and normalized to *β*-actin. Uncropped Western blots are shown in Supporting Information Figs. S15–S20.

### 2.27 Quantitation of cytokines in cell supernatants and peritoneal exudates by ELISA

Monocyte-derived macrophages (2 × 10^6^) were pre-incubated with vehicle (DMSO, 0.1%) or LCMs in RPMI 1640 medium containing 10% FCS, 100 U/mL penicillin, and 100 μg/mL streptomycin for 15 min at 37 °C in 5% CO_2_ and then polarized to M1 (20 ng/mL interferon-*γ*, 100 ng/mL LPS) or M2-like macrophages (20 ng/mL IL-4) for 48 h, as indicated above. Alternatively, monocytes (3 × 10^6^) were treated with LCMs or vehicle (DMSO, 0.1%) in RPMI 1640 medium containing 5% FCS, 100 U/mL penicillin, and 100 μg/mL streptomycin at 37 °C and 5% CO_2_ for 15 min, followed by stimulation with 100 ng/mL LPS for 24 h. The culture medium was collected and centrifuged (2000 × *g*, 4 °C, 10 min), and tumor necrosis factor-*α* (TNF*α*), IL-6, IL-1*β*, monocyte chemoattractant protein (MCP-1), and IL-10 were quantified in the supernatants using in-house-made DuoSet ELISA kits (DY210, DY206, DY201, DY279, DY217B; R&D Systems/Bio-Techne, Minneapolis, MN, USA). Levels of IL-1*β*, TNF*α*, IL-10 in peritoneal exudates were determined using DuoSet ELISA kits (DY401, DY417, DY410; R&D Systems/Bio-Techne) according to the manufacturer’s instructions.

### 2.28 Data and statistical analysis

Data were analyzed using Excel (Microsoft 365, Microsoft, Redmond, WA, USA), and statistical calculations were performed using GraphPad Prism 9 (GraphPad Software, San Diego, CA, USA). Bar, line, and symbol charts, volcano plots, and heatmaps were created with GraphPad Prism 9. Pathway diagrams were prepared using Cytoscape 3.9. (Cytoscape Consortium)^92^ and radar plots were generated with Mircosoft Excel 365 (Microsoft). Chromatograms were processed and visualized with Mircosoft Excel 365 (Microsoft). IC_50_ values were determined by non-linear curve fitting (sigmoidal dose–response function with variable slope) using GraphPad Prism 9.

Data are presented as the mean and standard error of the mean (SEM) of *n* observations, where *n* is the number of independent experiments or the number of animals in each group. For Fig. 2A and B, 3A–C, and 4A and B, samples were blinded by assigning a continuous code. Where specified, data were log-transformed for statistical analysis. For multiple comparisons, one-way or two-way analysis of variance (ANOVA) with Dunnett’s or Tukey’s *post hoc* test was used for ordinary and repeated measures. Two-tailed paired and unpaired *t*-tests were used to compare two groups, and a two-sided *α* correction was applied for multiple comparisons. Volcano plots show the mean difference in fold-changes and the negative log_10_(adjusted *P* value). Adjusted *P* values were calculated by two-tailed, multiple paired Student *t*-tests with correction for multiple comparisons using the Bonferroni-Dunn method (alpha level: 0.05). Outliers were optionally and within figure panels consistently detected using Grubb’s tests. The threshold for statistical significance was set at: ∗*P* < 0.05, ∗∗*P* < 0.01, ∗∗∗*P* < 0.001.

### 2.29 Data availability

The mass spectrometric lipidomics data generated in this study have been deposited in the Metabolomics Workbench database (an international repository for metabolomics data and metadata, metabolite standards, protocols, tutorials and training, and analysis tools^93^) under the accession code PR002125 (ST003442, ST003443, ST003444, ST003445, ST003446, ST003447, ST003448, ST003449, ST003450, and ST003451) [https://doi.org/10.21228/M8XZ5T].

## 3 Results

### 3.1 Screening for LCMs that stimulate fatty acid 15-(per)oxidation

Given the important role of ALOX15 in SPM biosynthesis, we placed natural vitamin E forms and their long-chain metabolites (LCMs) in a structural similarity network and visualized their ability to enhance the formation of carbon-15-oxidized products (*i*.*e*., 15-HETE) in A23187-activated PMNL by the size of the nodes (Fig. 1). To this end, we combined previously published data on 8 natural vitamin E forms and 12 LCMs^60^ with results from 12 LCMs either isolated from New Caledonian *Garcinia amplexicaulis*^78^, ^79^, ^80^ or semi-synthesized^62^^,^^75^^,^^80^^,^^94^. We found that carboxylated metabolites (depicted by blue nodes) induce an overall stronger increase in 15-HETE formation than alcohols and the parent vitamin E forms (COOH: 178 ± 19%; OH: 143 ± 11%; diOH: 107 ± 6%; non-functionalized: 98 ± 2%). In addition to the nature of the terminal functional group, the length and unsaturation of the side chain as well as the methylation of the chromanol ring have a substantial influence on 15-HETE formation. As a consequence, tocotrienolic acid **2** (*α*-TE-13′-COOH, *α*-garcinoic acid) is considerably more active in enhancing 15-HETE formation (2.1-fold increase) than the side chain-saturated *α*-T-13′-COOH (**1**) (Supporting Information Table S1), which is the most abundant systemic LCM on a standard diet^60^. Based on their ability to induce 15-HETE formation, we selected compounds **2** to **8** (Fig. 1) for further studies and compound **1** as a reference.

### 3.2 Specific LCMs induce PD/iso formation in innate immune cells

ALOX15 drives SPM biosynthesis by converting EPA, DPA, and DHA to fatty acid hydroperoxides, which are reduced to monohydroxylated SPM precursors^1^. We speculated that LCMs **2**–**8** are capable of inducing SPM biosynthesis and investigated their effect on the production of ALOX15-derived Rv and PD, ALOX15-and/or ALOX12-derived MaR, and SPM precursors (15-HETE, 14-HDHA, 17-HDHA, 17-hydroxydocosapentaenoic acid [17-HDPA], 18-HEPE) in activated innate immune cells. Lipid mediators were identified based on the mass of the analytes, specific fragments (MS/MS), and the retention times compared with identical standards in each batch for every analyte. Note that recent reports on the diversity of di- and trihydroxylated oxylipins suggest that some isoforms cannot be readily separated on non-chiral phases^95^. Therefore, we refer to these analytes as SPM and/or isomers (SPM/iso), although we excluded all obvious isomers that differ in MS/MS fragmentation or retention time from the analysis.

Our initial focus was placed on monocyte-derived human M2-like macrophages, which highly express ALOX15 and produce substantial amounts of SPM when exposed to SACM^14^ (Supporting Information Table S2). In fact, compounds **2**–**8** enhanced PD/iso and MaR2/iso biosynthesis (**2** > **3**–**8**), with **2** and **6** being most potent and reaching significance already at a concentration of 0.3 μmol/L (Fig. 2A and B, Supporting Information Table S3). Comparable results were obtained by trend for the hybrid macrophage subtype M2_LPS_ (Fig. 3A–C, Supporting Information Table S4), which combines M2-like features (CD163; less CD54) with enhanced M1 marker expression (CD54, CD80) (Fig. 3D). Levels of Rv/iso were instead hardly affected or even decreased.

Structure–activity relationship studies indicate i) that the tocotrienol *ω*-carboxylate **2** is more potent and effective in increasing PD/iso and MaR2/iso levels than its counterparts with a saturated side-chain (**1**) and ii) that partial saturation at the *Δ*11′ position is tolerated (**6** *vs.* **3**) (Figure 2, Figure 3A–C). iii) *α*-Substitution of the chromanol ring (**2**) is preferred over *β*- (**3**) and *δ*-substitution (**4**), and iv) the *ω*-alcohol **7** seems to be comparably active to the corresponding carboxylate **5**. v) *Cis*–*trans* isomerism at the terminal double bond (**4** *vs.* **5**) only has little effect on PD/iso and MaR2/iso production.

To assess the overall product formation by ALOX12 and ALOX15, we summarized their monohydroxylated products (formed *in situ* by the reduction of initial peroxides) and subdivided them into two groups based on regiospecificity. The mono-A15-OH group (*i*.*e*., 15-HETE, 15-HEPE, 17-HDPA, and 17-HDHA) comprises species that are preferentially produced by ALOX15, but not ALOX12. The mono-A12-OH group (*i*.*e*., 12-HETE, 12-HEPE, 14-HDPA, and 14-HDHA) includes species that can be produced by ALOX12, regardless of whether they are formed by ALOX12 or ALOX15 in the respective experimental setting. The levels of mono-A15-OH and mono-A12-OH, including SPM precursors, were not substantially increased or even decreased by LCMs (Figure 2, Figure 3A–C), suggesting either rapid metabolism of the monohydroxylated fatty acids or inhibition of ALOX12/15. This finding is not restricted to M2-like and M2_LPS_ macrophages but also applies to SACM-stimulated M1-like macrophages (Fig. 4A and B, Supporting Information Table S5) and human whole blood (Fig. 5A, Supporting Information Table S6), AA-treated platelets (Fig. 5B), and LPS-primed and A23187-activated human whole blood (Supporting Information Fig. S1) and PBMC (Fig. 5C, Supporting Information Table S7), which comprise monocytes, lymphocytes, natural killer cells, and dendritic cells, as well as platelets as contaminants^96^. Interestingly, different results were obtained for PMNL-platelet co-incubations. LCMs **2** and **7** (investigated here as examples) suppressed transcellular PD/iso biosynthesis, although they enhanced PUFA release and increased levels of mono-A15-OH (Fig. 5D). Compound **1**, used as reference, showed a comparable profile in PMNL-platelet co-incubations but was in other settings considerably less active in stimulating the biosynthesis of PD/iso, MaR2/iso, and/or SPM precursors.

We then asked whether the LCMs **2**–**8**, in addition to potentiating PD/iso and MaR2/iso production, could trigger the biosynthesis of these SPM in resting cells *per se*. Indeed, SPM/iso formation was upregulated (up to 8-fold for PD/iso) by the vast majority of LCMs at 3 μmol/L in non-stimulated cells, as exemplarily shown for M2_LPS_ macrophages (Fig. 3A–C). In contrast to activated immune cells (Figure 2, Figure 3A–C), this effect appears to be based on an enhanced release of PUFA and is associated with a broad upregulation of lipid mediators, including SPM precursors and Rv/iso (Fig. 3A–C). This release of PUFA (especially AA) was also evident in non-activated M1-like macrophages (Fig. 4A and B). Together, specific tocotrienol-derived *ω*-carboxylates (in particular **2**) and *ω*-alcohols (*e*.*g*., **7**) strongly and preferentially induce PD/iso and MaR2/iso biosynthesis in innate immune cells under inflammatory stress and maintain low basal SPM/iso biosynthesis by stimulating PUFA release in non-challenged cells.

### 3.3 SPM/iso production is associated with enhanced PGE_2_ biosynthesis and ALOX5 inhibition

LCMs **1**–**8** are direct inhibitors of ALOX5 that potently suppress the production of pro-inflammatory LT and other ALOX5 products in PMNL^60^. We confirm here that these LCMs inhibit ALOX5 product formation in SACM-activated M1-like (Fig. 4A and B), M2-like (Fig. 2A), and M2_LPS_ macrophages (Fig. 3A and B), PBMC (Fig. 5C), neutrophil-platelet co-cultures (Fig. 5D), and human whole blood (Fig. 5A). Other prominent changes in the lipid mediator profile are concentrated on PTGS products. On the one hand, the levels of major PG, including PGE_2_, PGD_2_, and PGF_2*α*_, increase moderately, in particular for *ω*-carboxylates with triene/diene structure (**2**–**6**) (Figure 2, Figure 3, Figure 4, Figure 5D). On the other hand, the production of TxB_2_ (the stable metabolite of TxA_2_) is suppressed. Since the biosynthesis of PTGS1-derived PGH_2_ is functionally coupled to its conversion to TxA_2_^10^, we speculated that the LCMs might inhibit PTGS1, as previously proposed for **1** and **4**^60^. Indeed, **2** shares the PTGS1-inhibitory activity of **1** in platelets at low micromolar concentrations (Fig. 5B), although neither LCM effectively inhibits isolated PTGS1 in a cell-free assay (Supporting Information Fig. S2). Human whole blood and again PMNL-platelet co-cultures are an exception: **2** and **7** either have little effect on prostanoid levels (Fig. 5A and D), overall reduce AA release and lipid mediator production (Fig. 5A), or preferentially upregulate PGE_2_ and less PGF_2*α*_ formation while having only minor effects on other prostanoids (Fig. 5D).

SPM, particularly Rv, are known to promote phagocytosis, while PGE_2_ inhibits this process in human M1- and M2-like monocyte-derived macrophages^5^^,^^13^^,^^97^, ^98^, ^99^ and murine M1 macrophages derived from bone marrow^100^^,^^101^. To assess whether LCM **2**-induced changes in the lipid mediator profile enhance phagocytic activity—a key process in inflammation resolution^2^—we treated human M1-and M2-like macrophages with PD1, PDX, PGE_2_, LCM **2**, or their combinations and monitored the uptake of fluorescent *Staphylococcus aureus* bioparticles over 9 h. The actin cytoskeleton-targeting agent cytochalasin D served as a control and effectively inhibited phagocytosis, as expected (Fig. 6A). PGE_2_ preferentially reduced phagocytic activity in M1-like macrophages, while RvD5 specifically enhanced it in M2-like macrophages. The combination of PD1 and PDX was effective in both phenotypes (Fig. 6A, line chart). However, the effects on the area under the curve, used as a measure of phagocytic efficacy, were weak, with RvD5 showing the most pronounced increase (1.3-fold, Fig. 6A, bar chart). Consistent with these findings, LCM **2**, which preferentially upregulates PD/iso and PGE_2_ (Figure 1, Figure 2, Figure 3A), significantly enhanced phagocytic activity, albeit with a minor effect size (Fig. 6B). In summary, our data suggest that LCM **2**-induced changes in the lipid mediator profile can modulate cellular processes, such as phagocytosis, that are linked to inflammation resolution. However, the physiological relevance of this effect remains uncertain, as the phagocytic activity of monocyte-derived macrophages appears to be poorly responsive to PGE_2_ and PD under our experimental conditions, making this model suboptimal for addressing this question.

### 3.4 ALOX15-dependent and -independent mechanisms of PD/iso formation

SPM biosynthesis is regulated by i) the supply of PUFA by PLA_2_ isoenzymes^45^^,^^46^, ii) changes in the expression, enzymatic activity, and subcellular distribution of ALOX15 isoenzymes^14^^,^^102^, and iii) putative effects on downstream enzymes, including hydrolases^54^^,^^103^. Non-canonical SPM biosynthesis instead depends on iv) acetylated PTGS2, which has ALOX15-like activity and synthesizes LX, Rv, and PD species with inverted stereochemistry as compared to ALOX15 products. Whether ligand-induced changes in ALOX regioselectivity, as recently described for ALOX5^34^^,^^104^, further contribute to SPM biosynthesis, is poorly understood. To elucidate the molecular mechanism by which LCMs stimulate PD/iso biosynthesis, we systematically addressed the above mentioned (putative) mechanisms.

By monitoring the mitochondrial dehydrogenase activity of monocytes and M1-and M2-like macrophages, we show that **1**, **2**, and **7** have no cytotoxic effects (Supporting Information Fig. S3) that might contribute to SPM/iso biosynthesis in Figure 2, Figure 3 *via* phospholipid degradation. This finding is consistent with two other observations that together exclude a major role for PUFA in the induction of PD/iso biosynthesis by LCMs in activated innate immune cells. First, **2**–**8** did not markedly enhance PUFA release (Figure 2, Figure 3, Figure 4, Figure 5D). Second, **2** and **7** substantially enhanced PD/iso biosynthesis, even when the need for intracellular PUFA mobilization is bypassed by the supply of exogenous DHA (Fig. 6C and D). The situation may be different for non-activated innate immune cells, as suggested by the analysis of free PUFA (Fig. 3A and B). In support of this hypothesis, DHA supplementation of unstimulated M2-like macrophages diminished the ability of **2** (but interestingly not of **7**) to further induce PD/iso biosynthesis, whereas **1** surprisingly became able to upregulate AT-PD1/iso (Fig. 6D). Together, stimulated PUFA release is not essential for the induction of PD biosynthesis by LCMs in activated innate immune cells, but defines how effectively LCMs raise the low basal PD/iso generation in the absence of pro-inflammatory stimuli. Note that we excluded PD1 from the analysis of DHA-treated samples because the fatty acid is autoxidized under cell-free assay conditions to PD1 or an isobaric oxylipin that co-elutes with PD1 and shares the transition in MS/MS analysis used for PD1 quantitation (Supporting Information Fig. S4 and Table S8), consistent with recent concerns about the isomeric diversity of PD-like oxylipins^95^.

To distinguish between short- and long-term effects on PD/iso biosynthesis, we performed kinetic studies in SACM-stimulated M2-like macrophages. Compound **2** increased PD1/iso and AT-PD1/iso levels already within 30 min and strongly and specifically stimulated AT-PD1/iso production from 6 h onwards (Fig. 6F). In contrast, the inhibition of ALOX5 product formation, which we investigated as control, was only evident at early time points and perished later (Fig. 6H). Based on these findings and following our initial experimental design, we first focused on potential mechanisms that may contribute to the short-term induction of PD/iso biosynthesis. On the one hand, compound **2** decreased the availability of monohydroxylated PUFA characteristic for ALOX12 and/or ALOX15 product formation in activated macrophage populations (Figure 2, Figure 3, Figure 4A) and monocytes (Fig. 5C). This finding excludes that **2** shifts the regioselectivity of ALOX5 from 5- to 12-dioxygenation, as previously demonstrated for 3-acetyl-11-keto-*β*-boswellic acid (AKBA)^104^. On the other hand, we asked whether **2** directly activates ALOX15, as previously reported for AKBA^34^, which was not the case, as shown in a cell-free activity assay based on the conversion of AA to 15-H(p)ETE in homogenates of M2-like macrophages (Fig. 6E, Supporting Information Fig. S5). To investigate whether the LCMs are intracellularly enriched and thus activate additional low-affinity targets, we measured the intracellular concentrations of **1** and **2** by UHPLC-MS/MS. Compound **2** was less enriched than **1** (Fig. 6G), ruling out this hypothesis.

The peroxidases and epoxide hydrolases that convert the ALOX15 product 17-HpDHA to PD are largely enigmatic. Among the few proposed enzymes is LTA_4_ hydrolase (LTA4H), which hydrolyzes LTA_4_ to LTB_4_^1^^,^^31^^,^^105^. If LCMs stimulate PD/iso biosynthesis by activating LTA4H, we would expect the associated suppression of ALOX5 product formation to be less severe for LTB_4_ than for non-enzymatically produced LTB_4_ isomers and other ALOX5 products (*i*.*e*., 5-HETE and 5-HEPE). However, the ratio of LTB_4_ to its isomers is decreased, especially in activated M2 and M2_LPS_ phenotypes (Fig. 7A Supporting Information Fig. S6), which rather precludes that LTA4H activation promotes SPM/iso biosynthesis. This preference for decreasing LTB_4_ biosynthesis is diminished after 1 h but is still evident up to 24 h after treatment with **2** (Fig. 7A, Supporting Information Fig. S7).

Next, we investigated whether **2** hijacks PTGS2 for AT-PD/iso biosynthesis in M2-like macrophages. A similar mechanism has been reported for acetylsalicylic acid, for example, which causes PTGS2 to undergo conformational changes through the acetylation of Ser516^106^^,^^107^. In such a case, we would expect that the saturation of PTGS2 acetylation by excess acetylsalicylic acid would attenuate the increase in PD/iso and AT-PD/iso production caused by **2**. Compound **2** markedly increased PD1/iso and AT-PD1/iso formation despite the addition of acetylsalicylic acid after 15 min and 24 h (Fig. 7B), which rather excludes that acetylation-like changes alter the PTGS2 substrate specificity towards PD/iso biosynthesis. Nevertheless, PTGS2 appears to be a relevant player promoting both PD1/iso and AT-PD1/iso biosynthesis because its selective inhibition by celecoxib moderately attenuates PD/iso formation (but not 17-H(p)DHA production and DHA release), both on the short (15 min; *P* = 0.37 for PD1, LCM **2**, *t*-test of log data) and long term (24 h; *P* = 0.17 for PD1, LCM **2**; *t*-test of log data), resulting in a significant upregulation of PD/iso biosynthesis only in absence of celecoxib (Fig. 7B, Supporting Information Fig. S8). Note that off-targets of celecoxib have been reported, including ALOX5^108^, which, however, is not substantially inhibited by celecoxib in M2-like macrophages under our experimental conditions (Supporting Information Fig. S8), in agreement with a previous report^38^. Conclusively, the increase in PD/iso biosynthesis by LCMs seems to be partially mediated by a poorly understood PTGS2-dependent mechanism, possibly involving PGE_2_, whose levels are increased by treatment with **2** (Figure 2, Figure 3, Figure 4A and B) and strongly decreased by PTGS2 inhibition (Fig. 7B). This finding may gain further relevance in light of recent reports on common anti-inflammatory and pro-resolving signaling routes for PD and PGE_2_^100^.

The subcellular localization of ALOX15 has been proposed to be a critical factor defining the cellular capacity for ALOX15 product and SPM biosynthesis^13^^,^^34^. Specifically, ALOX15 (which, in contrast to ALOX15B, is highly expressed in M2-like macrophages^88^) has been shown to translocate to particulate compartments within the cytoplasm. Compound **2** induces such a redistribution of ALOX15 in non-stimulated M2-like macrophages, thereby increasing the cellular proportion of cytoplasmic ALOX15-rich locales, but without reaching the efficiency of SACM (Fig. 7C and D). The effect of **2** on ALOX15 translocation is overcompensated in M2-like macrophages challenged with SACM (Fig. 7C and D), suggesting that this mechanism contributes to PD biosynthesis in non-activated but not activated innate immune cells.

We next investigated long-term mechanisms that might promote PD/iso biosynthesis upon treatment with LCMs. Initially, we focused on macrophage polarization, which is known to reprogram lipid mediator metabolism^38^^,^^109^, but analysis of M1 and M2 surface markers by flow cytometry did not reveal any prominent changes (Figure 2, Figure 4C). In this context, we also investigated the protein expression of key enzymes in PD/iso biosynthesis (PLA2G4A, ALOX15, ALOX15B, optionally PTGS2) after treatment with LCMs for 48 h (Fig. 7E, Supporting Information Fig. S9). Interestingly, compound **7** enhanced ALOX15 expression in M2-like macrophages (Fig. 7E), but none of the biosynthetic enzymes was consistently upregulated for both LCMs studied (**2** and **7**).

Our data indicate that LCMs, such as **2**, sustain basal PD/iso biosynthesis by modestly mobilizing DHA. Additionally, in unstimulated cells, they promote the translocation of ALOX15 to particulate cytoplasmic compartments, which have been proposed to harbor active ALOX15 and contribute to SPM biosynthesis^13^^,^^38^. In activated M2-like macrophages, the increase in PD biosynthesis upon treatment with **2** appears to involve additional mechanisms, possibly PTGS2-dependent. However, these mechanisms remain diffuse and do not align with previously established pathways for enhancing SPM/iso production.

### 3.5 LCM **2** stimulates PD/iso formation and relieves murine peritonitis *in vivo*

**Figure 8:**
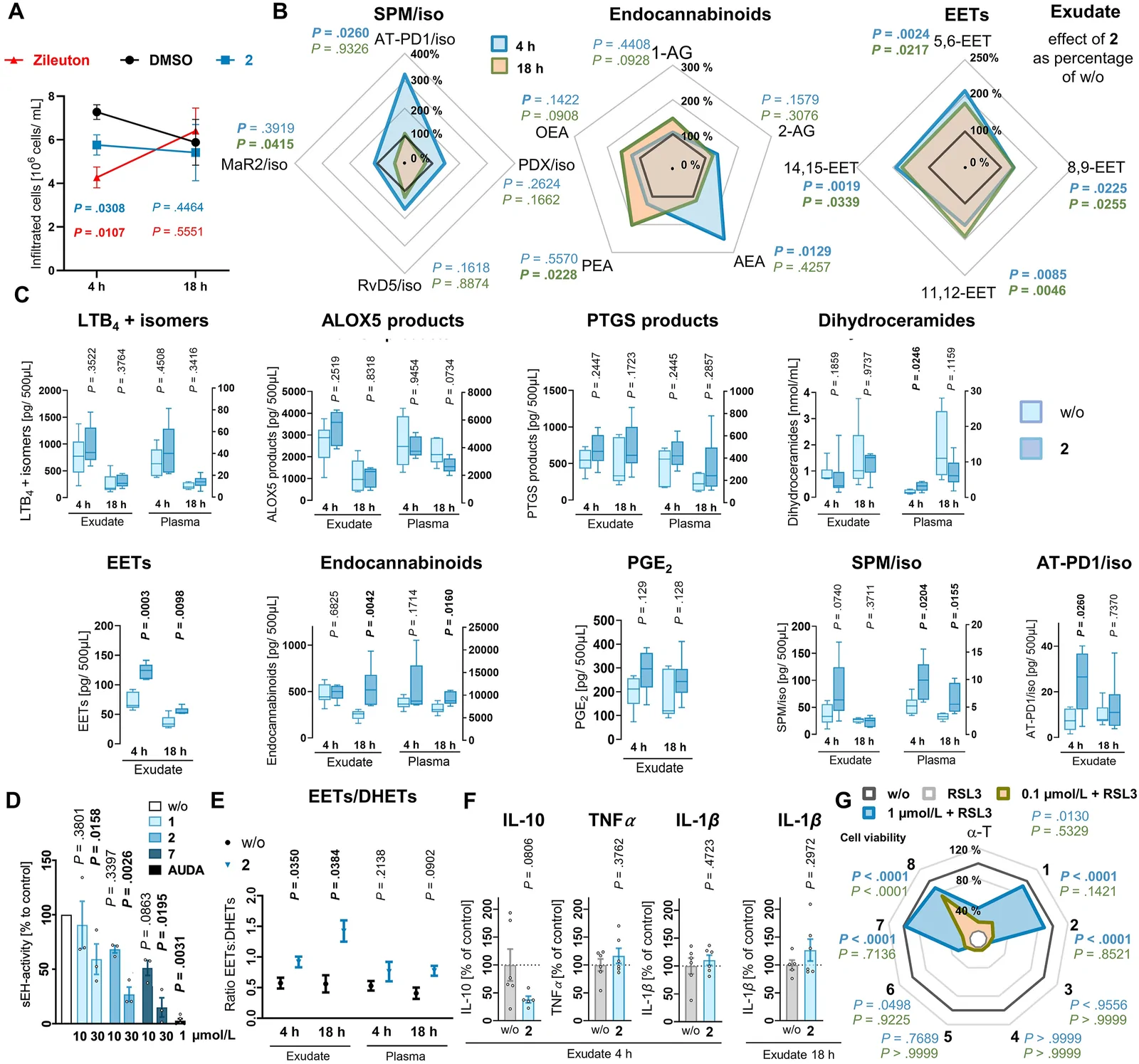
LCMs favorably manipulate PD/iso, epoxyeicosatrienoic acid (EET), endocannabinoid and sphingolipid metabolism in experimental peritonitis and protect hepatocytes from ferroptosis. (A–C, E, F) Mice received vehicle (2% DMSO, ‘w/o’), **2** (10 mg/kg, i.p.), or zileuton (10 mg/kg, i.p.) 30 min prior to zymosan injection and were killed after 4 h or 18 h. (A) Immune cell infiltration into the peritoneal cavity. (B, C) Levels of lipid mediator subgroups and individual species in the exudate and plasma. (D) Human recombinant EPHX2 activity after pretreatment with vehicle (DMSO, 2%), LCMs (10 or 30 μmol/L), or AUDA (1 μmol/L) for 15 min. (E) Ratio of EET to DHET in exudate and plasma as an indicator for epoxidase activity. (F) Levels of cytokines in the exudate. (G) Human HepaRG hepatocytes (1 × 10^4^ cells/well) were seeded in a 96-well plate and, after 24 h, treated with either the vehicle control (0.5% DMSO), the ferroptosis inducer RSL3 (0.3 μmol/L), or a combination of RSL3 with *α*-tocopherol or LCMs. Cell viability was assessed after 48 h by measuring the conversion of 3-(4,5-dimethylthiazol-2-yl)-2,5-diphenyltetrazolium bromide (MTT) by cellular dehydrogenases. Mean (B, G), mean ± SEM (A, E) and single data (D, F), or mean with boxes showing the 25th to 75th percentiles and whiskers indicating smallest and largest values (C) from *n* = 4–6 mice per group (A–C, F, G) or *n* = 3 independent experiments (D, G). *P* values *vs.* vehicle control (A–F) or the RSL3-treated control (G); two-tailed paired (A, D) or unpaired (B, C, E, F) Student *t* test (B, C, E) of log data (A, D, F) or repeated measures one-way ANOVAs with Dunnett’s *post hoc* tests of log data (G). Data for vehicle are identical to Waltl et al^160^.

Whether LCM **2** induces a lipid mediator class switch under (patho)physiological conditions *in vivo* was investigated in zymosan-induced peritonitis in mice. This self-resolving model of inflammation is primarily driven by peritoneal macrophages and infiltrated neutrophils, which produce abundant LT, prostanoids, and other pro-inflammatory lipid mediators^85^^,^^110^^,^^111^. SPM, including PD, are generated at later stages, and were proposed to initiate the termination of the inflammatory reaction and stimulate the egress of immune cells from the peritoneal cavity^2^^,^^15^. Compound **2** (10 mg/kg), administered i.p. to prevent side-chain truncation and conjugation by hepatic first-pass metabolism^112^, attenuated the influx of innate immune cells in the acute phase of inflammation (4 h post zymosan injection), but was less efficient than the reference ALOX5 inhibitor zileuton (Fig. 8A). We ascribed the lower efficacy of **2** compared to zileuton to its failure to reduce levels of LTB_4_, which is derived from ALOX5 and represents a potent chemoattractant^10^. Compound **2** did not reduce peritoneal or systemic LTB_4_ levels, nor did it markedly lower the concentration of other ALOX5 products (Fig. 8C). On the other hand, **2** substantially increased PD/iso levels during the acute phase of inflammation (Fig. 8B and C). When the inflammatory reaction subsides 18 h after zymosan injection^60^, PD/iso concentrations returned to control levels in the peritoneal cavity (Fig. 8B and C), but remained elevated in the circulation (Fig. 8C, Supporting Information Fig. S10). Other SPM/iso (excluding MaR2/iso but including RvD5/iso) and the SPM precursors 17-HDHA, 17-HDPA, and 14-HDHA showed a similar pattern as PD/iso and were markedly upregulated in the peritoneal exudate, specifically during acute inflammation (4 h post zymosan, Fig. 8B and C, Supporting Information Fig. S10 and Table S9). Conclusively, **2** governs a pro-resolving lipid mediator profile that is expected to promote resolution of inflammation, as suggested by the lower number of infiltrated immune cells.

### 3.6 Concomitant manipulation of EET, endocannabinoid, and sphingolipid metabolism positively impacts local and systemic lipid mediator profiles

The LCMs **1** and **4** have previously been reported to induce aberrant ceramide and sphingomyelin metabolism^113^ and to suppress LPS signaling, thereby reducing cytokine release and PTGS2-dependent PG biosynthesis^64^^,^^65^^,^^114^. To gain deeper insights into the mechanisms by which **2** alleviates inflammation in murine peritonitis, we analyzed pro- and anti-inflammatory cytokines and extended our metabololipidomics approach to prostanoids, EET, endocannabinoids, and sphingolipids, which include potent immunomodulatory lipid mediators^2^^,^^115^, ^116^, ^117^, ^118^.

Compound **2** markedly increased the levels of EET and EpDPA preferentially in the exudate during the acute phase, and this effect was maintained up to 18 h (Fig. 8B and C). EET are generated from AA by cytochrome P450 monooxygenases, exert anti-inflammatory activities, and are degraded to inactive DHET by EPHX2 and other hydrolases^119^^,^^120^. Mechanistically, **2** and **7** inhibit EPHX2, as shown for the isolated human enzyme at micromolar concentrations (Fig. 8D). In support of EPHX2 as an *in vivo* relevant target of **2**, the LCM decreased DHET levels within 18 h in both the peritoneal exudate and plasma (Supporting Information Fig. S10), thereby increasing the ratio of EET to DHET (Fig. 8E), indicative of epoxide hydrolase activity^50^. Additional targets of **2** that contribute to the increase in EET levels may exist.

Endocannabinoids include monoglycerides and *N*-acylethanolamines with pleiotropic biological functions, exerting anti-nociceptive, immunoregulatory, anti-inflammatory, metabolic regulatory, and/or neuroprotective activity^121^. Compound **2** notably upregulated peritoneal and systemic endocannabinoid levels, reaching significance at 18 h post zymosan injection (Fig. 8B and C). Changes in sphingolipid metabolism by **2** were restricted to dihydroceramides, whose systemic concentrations were consistently upregulated in the acute phase of inflammation (Fig. 8C and Supporting Information Fig. S11 and Table S10). While dihydroceramides have long been considered as inactive precursors of ceramides, they are now known to have multiple activities, among others, in stress protection by promoting autophagy^53^.

The concentration of prostanoids increased by trend in plasma and peritoneal exudate upon administration of **2**, during both early and late phases of inflammation (Fig. 8C), as observed for activated human innate immune cells *in vitro* (Figure 2, Figure 3, Figure 4A). Free PUFA levels were not elevated (Supporting Information Fig. S12), ruling out a contribution of DHA and AA mobilization to SPM, EET, endocannabinoid, and prostanoid biosynthesis.

We also did not observe substantial changes in the peritoneal concentrations of pro-inflammatory cytokines (TNF*α*, IL-1*β*). The availability of IL-10, a cytokine with anti-inflammatory properties^122^, even tended to decrease (Fig. 8F). Again, these results are consistent with our data from cell-based studies, in which neither **1**, **2**, nor **7** suppressed the release of TNF*α*, IL-1*β*, IL-6, and MCP-1 from LPS-stimulated monocytes and M1-and M2-like macrophages (Supporting Information Fig. S13). Together, compound **2** interferes with lipid mediator biosynthesis and metabolism at multiple stages, resulting in an overall anti-inflammatory, anti-nociceptive, pro-resolving, and potentially stress-adaptive lipid mediator profile that undergoes dynamic changes from the acute to the resolving phase of inflammation.

### 3.7 LCM **2** exhibit anti-ferroptotic activity superior to α-tocopherol

Tocopherols and tocotrienols are lipophilic radical scavengers that inhibit lipid peroxidation and ferroptosis^123^^,^^124^. Because these processes are closely linked to necroinflammatory mechanisms^125^ driving degenerative and metabolic diseases such as neurodegeneration and chronic liver disease^126^, we investigated the effects of LCMs on ferroptosis in human HepaRG hepatocytes, a surrogate for primary human hepatocytes^127^. Cells were treated with the glutathione peroxidase 4 inhibitor RSL3 to induce ferroptosis, and the anti-ferroptotic activity of LCMs **1**–**8** was evaluated in comparison to *α*-tocopherol. The *α*-substituted *ω*-carboxylates **1** and **2**, as well as the *ω*-alcohols **7** and **8**, demonstrated greater potency in inhibiting ferroptosis than *α*-tocopherol (Fig. 8G). In contrast, the *β*- and *δ*-substituted *ω*-carboxylates **3**–**6** displayed weaker inhibitory activity. These findings suggest that both the parent vitamin E form and the *ω*-modifications critically determine the anti-ferroptotic potential of LCMs. Among them, LCM **2** emerged, together with other LCMs, as particularly potent in suppressing ferroptosis.

## 4 Discussion

With the growing recognition of failed inflammation resolution as a key factor in chronic inflammatory diseases, including intestinal inflammation, periodontal diseases, and cardiovascular disorders^24^^,^^28^^,^^128^, interest in resolution therapeutics has increased^129^. However, the field faces numerous challenges, and no rationally designed resolution-inducing agents have yet reached the market. Given the complexity of inflammatory processes and their resolution, the most effective strategies for therapeutic intervention remain unclear. One particularly debated approach in recent years involves SPM^129^. Beyond challenges in their analytical detection, concerns have been raised about the ability of LC–MS/MS techniques to reliably distinguish SPM from structurally similar, often undefined, isomeric di- and trihydroxylated PUFA^130^^,^^131^. Further criticism refers to the physiological relevance of proposed SPM receptors and signaling cascades, as well as the feasibility of achieving physiologically relevant SPM concentrations *in vivo*^18^. This concern is particularly pronounced for trihydroxylated species, which were not considered in this study. Despite these uncertainties, there is little doubt regarding the potent pro-resolving effects of exogenously administered SPM, including the PD highlighted here, in preclinical models^129^^,^^132^, ^133^, ^134^, ^135^, ^136^. For instance, PD (0.02–1 μg), delivered intravenously, intraperitoneally, or endotracheally in mice, have been shown to reduce airway inflammation^133^^,^^137^, alleviate high-fat diet-induced insulin resistance and inflammation^138^, and promote tissue repair^135^. The apparent discrepancy between the inflammation-resolving efficacy of SPM at therapeutic concentrations and the uncertainty of their endogenous availability in disease contexts has motivated our search for small molecules capable of shifting the lipid mediator profile from pro-inflammatory to anti-inflammatory and pro-resolving states.

In this study, we identified specific LCMs (endogenously produced in liver^57^^,^^139^) that enhance the biosynthesis of two groups of dihydroxylated SPM, predominantly PD/iso and, to a lesser extent, MaR/iso in innate immune cells. Notably, this effect was observed across different populations of human monocyte-derived macrophages, both under resting conditions and in response to a sterile pro-inflammatory stimulus. The upregulation of PD/iso levels was further confirmed *in vivo* using a self-resolving mouse model of peritoneal inflammation. In contrast to metabolite **1**, which was found to moderately increase Rv/iso biosynthesis, especially that of RvD5/iso, consistent with previous studies^60^, metabolite **2** instead inhibited Rv/iso production, particularly at higher concentrations. This might be due to the suppression of ALOX5 by **2** as this isoenzyme contributes to RvD formation in SACM-activated macrophages^13^^,^^38^.

Efficient SPM biosynthesis depends on several key factors, including the release of PUFA from membrane phospholipids or alternative stores and the enzymatic activity of ALOX isoforms, which may require translocation to specific subcellular compartments to (di)oxygenate free PUFA^140^. This has been well-documented for ALOX5 and is also known for ALOX15, often considered the rate-limiting enzyme in SPM biosynthesis^14^. These processes are tightly regulated by intracellular Ca^2+^ influx and modulated by mitogen-activated protein kinases^140^, with the rate of Ca^2+^ influx playing a critical role^14^. Specifically, a gradual increase in intracellular Ca^2+^ levels (*e*.*g*., induced by SACM) favors SPM production, whereas a rapid and robust Ca^2+^ surge (*e*.*g*., induced by A23187) promotes the synthesis of diverse pro-inflammatory lipid mediators in phagocytes^14^. Addressing these aspects, we found that the mechanisms by which LCMs enhance PD/iso biosynthesis vary depending on the experimental context. They include an increased availability of fatty acid substrates, the translocation of ALOX15 to particulate compartments in the cytoplasm, and a potential involvement of PTGS2 activity. Note that a limitation of this and previous studies^14^ is that the mechanistic link between ALOX15 translocation and SPM biosynthesis remains incompletely understood. The role of PTGS2 in PD/iso biosynthesis is also unclear. While our inhibitor studies suggest that PTGS2 contributes to PD biosynthesis independently of Ser516 acetylation, definitive evidence to exclude the involvement of the acetylation mechanism described for acetylsalicylic acid would require site-directed mutagenesis to prevent PTGS2 acetylation. However, the requirement for substantial PD/iso biosynthesis in monocyte-derived macrophages poses a challenge for such approaches, as these cells are difficult to transfect, and their inability to proliferate precludes selection-based strategies.

Compared to the more common PTGS1/2 and ALOX5 inhibitors, small molecules that significantly enhance SPM/iso biosynthesis are rare. One of the best-characterized mechanisms has been described for AKBA, which may bind to an allosteric site of ALOX5 to activate the enzyme and induces a shift in ALOX5 regioselectivity toward 12-lipoxygenation^34^, a mechanism we ruled out for compound **2**. Other small molecules, including celastrol^36^, chalcone derivatives (MF14/15)^35^, and cannabidiol^44^, inhibit 5-lipoxygenase-activating protein (ALOX5AP)^33^^,^^141^ and enhance ALOX15 product formation and consequently SPM biosynthesis by stimulating PUFA release through Ca^2+^-dependent or independent mechanisms. As with AKBA, this occurs via allosteric activation of ALOX15 in the case of cannabidiol. While LCMs share some mechanistic similarities with these compounds, they exhibit distinct characteristics. Notably, LCMs selectively induce PD/iso and less MaR2/iso production (among SPM-like structures including LX/iso and Rv/iso) in human innate immune cells. Unlike other small molecules that primarily increase PUFA availability, LCMs do not rely on further enhancing PUFA mobilization, as evidenced by unchanged PUFA levels under specific conditions, such as in activated M2 macrophages. For other classes of small molecules, distinct mechanisms predominate. For instance, ALOX5AP antagonists primarily redirect substrates^33^^,^^38^^,^^141^, whereas glucocorticoids upregulate ALOX15B expression while repressing ALOX15^88^. The mechanisms underlying SPM/iso or SPM precursor biosynthesis in innate immune cells remain unclear for several small molecule classes, including biflavonoids from *D. cambodiana*^37^, rotationally restricted curcuminoids^39^, benzenesulfonamide derivatives^40^, ginkgolic acid^41^, benzoxanthene lignans^42^, oxymetazoline^43^, and dihydrophenanthrenoids^81^. LCMs rank among the small molecules with the lowest effective concentrations known to stimulate PD biosynthesis. In cell-based settings (*e*.*g*. SACM-stimulated M2-like macrophages), they increase PD formation at concentrations as low as 0.3 μmol/L, comparable only to glucocorticoids (0.1 μmol/L)^88^ and specific biflavonoids (0.3 μmol/L)^37^.

Our search for small molecules targeting lipid mediator networks in the control of inflammation and homeostasis extended beyond modulating SPM biosynthesis to include other immunoregulatory mediators involved in humoral communication, such as additional lipid mediator classes and cytokines. This comprehensive approach is particularly important given the sensitivity of lipid mediator networks to external interference and their ability to readjust dynamically^1^. Moreover, individual lipid mediators can exert diverse and sometimes opposing effects depending on the target tissue, concentration, and kinetics, functioning as pro-inflammatory, anti-inflammatory, or homeostatic signaling molecules^4^^,^^129^. Additionally, combinations of lipid mediators do not necessarily produce additive effects but may result in either synergistic or antagonistic interactions^142^. Thus, a detailed understanding of lipid mediator profiles is essential, though defining concrete signatures for pro-resolving and anti-inflammatory efficacy remains a challenge. The availability of well-characterized tool compounds, such as the LCMs presented here, may help address these limitations and provide valuable insights into the complex regulatory networks governing inflammation resolution.

In addition to stimulating PD/iso production, LCM **2** increases the levels of prostanoids, EET, endocannabinoids, and dihydroceramides, as observed in experimental peritonitis. Overall, we consider this shift beneficial: i) EET inhibit nuclear factor-*κ*B signaling in both immune and non-immune cells, thereby reducing pro-inflammatory cytokine and lipid mediator production, as well as leukocyte adhesion, infiltration, and activation, partially through cellular hyperpolarization^119^^,^^143^. ii) Endocannabinoids exert immunomodulatory effects primarily via the CB2 receptor and alternative non-cannabinoid receptor-related pathways^144^^,^^145^. They are generally regarded to inhibit the onset of inflammation and alleviate ongoing inflammation^145^^,^^146^, protect cells from inflammatory damage^147^, and reduce hyperalgesia^146^^,^^148^. However, under certain conditions, endocannabinoids have also been reported to exhibit immunostimulatory effects^149^. iii) The impact of increased prostanoid formation, including PGE_2_, is more complex to assess. While the moderate elevation in prostanoid levels observed here may serve as a prerequisite for initiating the lipid mediator class switch^11^ and could even contribute to anti-inflammatory effects, caution is needed. Prostanoids like PGE_2_ are also key drivers of inflammation, fever, and pain^4^^,^^9^. Consequently, it cannot be ruled out that prostanoid upregulation partially counteracts the anti-inflammatory and pro-resolving effects of LCMs, potentially leaving room for further structural optimization of LCMs. Mechanistically, LCMs inhibit EPHX2, a key epoxide hydrolase responsible for deactivating EET^150^. However, the mechanisms by which LCMs elevate endocannabinoid, dihydroceramide, and prostanoid levels remain unclear.

In agreement with our previous results^60^, the here identified LCMs also demonstrated potent inhibition of pro-inflammatory LT biosynthesis, though the effect was highly context-dependent. In activated human innate immune cells, LCMs effectively suppressed ALOX5 product formation. However, in experimental peritonitis *in vivo*, no reduction in ALOX5 product levels was observed, either at the site of inflammation or systemically. The underlying reasons for this discrepancy remain unclear, and species-specific differences cannot be ruled out.

While these LCMs may serve as lead structures for the development of inflammation-resolving drugs, an important question remains: are the observed mechanisms physiologically relevant, and do they respond to vitamin E status, specifically *α*-tocotrienol? Natural vitamin E forms are known to be metabolized in the liver into *ω*-carboxylates, some of which can reach concentrations of up to 0.7 μmol/L in the liver and 0.1 μmol/L in the plasma of mice on a vitamin E-enriched diet^151^. Other *ω*-carboxylates, including compound **1**, have been detected in human and rodent circulation under a normal diet (5–50 nmol/L) and have been found to accumulate at sites of inflammation, reaching concentrations of up to 0.3 μmol/L^60^. While these concentrations, at least in the liver and potentially at inflammatory sites, fall within the range at which compound **2** induces a lipid mediator class switch, further studies are needed to determine its endogenous relevance. Specifically, it remains unclear whether dietary strategies could lead to a localized increase in metabolite **2** concentrations and whether such an accumulation would have functional consequences. Given the substantial difference between physiological *α*-tocopherol and LCM concentrations^60^^,^^61^, the superior anti-ferroptotic activity of LCM **2** compared to *α*-tocopherol may contribute to the anti-ferroptotic radical scavenging capacity of cells and hold pharmacological relevance. However, it is unlikely to have major nutritional significance under normal vitamin E status. In contrast, LCM **1**, which shares the high anti-ferroptotic activity with **2**, reaches systemic concentrations in humans and mice that fall within the range of effective anti-ferroptotic concentrations, even under basal conditions^60^.

The potential of LCM **2** as a lead compound for drug development remains largely unexplored, with limited information available beyond our current study on the *in vivo* pharmacology of this specific *α*-isoform of garcinoic acid. In contrast, *δ*-garcinoic acid (**4**), a vitamin E metabolite abundantly found in *Garcinia kola* nuts, traditionally used in African medicine as an anti-inflammatory remedy^152^, has been studied more extensively. Compound **4** exhibits a broad range of pharmacological activities, including potent antioxidant properties^153^ and inhibition of ALOX5^60^, microsomal prostaglandin E synthase-1^62^, and toll-like receptor 4-activated pro-inflammatory pathways, such as nuclear factor-*κ*B signaling in innate immune cells^154^^,^^155^. Additionally, compound **4** acts as an agonist of pregnane X receptor^156^ and peroxisome proliferator-activated receptor *γ*^67^^,^^157^, suppresses NLRP3 inflammasome activation and pyroptosis^158^, and has shown efficacy in various *in vivo* models. For example, it impairs inflammatory responses in an atherosclerotic mouse model (1 mg/kg, i.p., weekly)^114^, reduces *β*-amyloid deposition in the mouse brain (200 mg/kg, *p*.*o*., daily)^159^, induces NRF2-dependent efferocytosis and promotes inflammation resolution in a mouse colitis model (1 mg/kg, i.p., daily)^74^, and accelerates wound healing in mice (5 μmol/L, onto the wound)^70^.

Pharmacokinetic studies on garcinoic acids are rare, but structurally related LCMs have been investigated in more detail. For instance, LCM **1** is orally active^76^ and has also been applied topically to treat skin inflammation in mice^70^. Similarly, *α*-amplexichromanol, which features two terminal alcohol groups instead of an *ω*-carboxylate, is orally bioavailable and achieves plasma concentrations of 0.03–0.9 μmol/L within 90 min at a single peroral dose of 10 mg/mL^75^. These concentrations are in the range of the effective levels required for 5-LOX inhibition (IC_50_ = 0.4–0.6 μmol/L) in innate immune cells^60^^,^^75^. However, a potential limitation of LCMs, such as LCM **1**, is hepatic metabolism to less active short-chain *ω*-carboxylates^60^. This drawback can be mitigated through minor structural modifications, as demonstrated with *α*-amplexichromanol^75^.

In this study, we focused on lipid mediator networks associated with inflammation and its resolution, and identified a favorable redirection driven by specific LCMs. Whether these changes translate into resolution-associated processes, such as efferocytosis, neutrophil trafficking, macrophage polarization, or reduced immune cell activation^2^, remains less well understood. Multiple animal studies have shown pro-resolving activities of PD when administered at doses of 0.02 to 1 μg^133^^,^^135^^,^^137^^,^^138^. However, it is unclear whether LCM **2** and other LCMs induce a sufficiently strong and sustained increase in local and systemic PD concentrations to efficiently trigger inflammation resolution. It is also unknown how the distinct lipid mediator landscape created by LCMs—where PDs represent only one of several coordinated rearrangements—acts in this context. First indications of an inflammation-resolving activity of **2** stem from our *in vivo* model of zymosan-induced peritonitis. Here, LCM **2** reduced immune cell counts (mainly neutrophils^85^) in the peritoneal cavity without substantially decreasing the levels of chemotactic lipid mediators or cytokines tested. This raises the possibility that the associated increase in AT-PD1/iso and other SPM/iso, together with broader shifts in the lipid mediator profile, either limits immune cell infiltration or promotes their egress. Further supporting its potential pro-resolving activity, LCM **2** significantly enhanced the phagocytic activity of both M1- and M2-like macrophages, albeit to a minor extent, consistent with the poor responsiveness of these cells to PD. We also examined the effects of LCM **2** on macrophage polarization toward M1 and M2 phenotypes and assessed cytokine release from activated monocytes as well as M1- and M2-like macrophages. These experiments, however, were designed to study the effects of LCM **2** on LPS-, interferon-*γ*-, and/or IL-4-induced pathways, rather than to determine whether initial PD/lipid mediator biosynthesis can modulate immune cell responses at a later stage. Taken together, LCM **2** redirects the lipid mediator profile from an inflammation-associated to a resolution-associated state, and preliminary observations are consistent with (but do not yet establish) a link between this shift and inflammation resolution in a (patho)physiological context.

## 5 Conclusions

The LCM **2** has been identified as potent bioactive metabolite of tocotrienols and promising lead structure that favorably reshapes the lipid mediator profile of innate immune cells and limits inflammation. Specifically, it enhances the formation of PD or isomers, a subclass of SPM that promote the resolution of inflammation. This occurs through the stimulation of PUFA release, induction of ALOX15 translocation, and likely the involvement of PTGS2. Additionally, compound **2** prevents the degradation of anti-inflammatory EET presumably by inhibiting EPHX2, an enzyme considered a promising drug target^150^. Compound **2** also increases the availability of immunoregulatory and analgesic endocannabinoids while suppressing pro-inflammatory LT biosynthesis by targeting ALOX5. Furthermore, it moderately elevates prostanoid and dehydroceramide levels and exhibits potent anti-ferroptotic activity. Its precise effects vary depending on the innate immune cell population, stimulatory conditions, and experimental settings. Based on this thorough characterization, LCM **2** could be a useful chemical tool for dissecting the intricate profile–function relationships within lipid mediator networks. Moreover, it represents a promising lead structure for anti-inflammatory and pro-resolving small molecule drugs. As an endogenous hepatic vitamin E metabolite, it may also contribute to immunoregulation, particularly in response to specific dietary conditions.

## Author contributions

Stephan Permann - Methodology, Investigation, Visualization, Writing - Original Draft, Writing - Review & Editing. Elena Brunner - Methodology, Investigation, Visualization, Writing - Review & Editing. Khaled Alsabil - Investigation. Markus Werner - Methodology, Visualization, Writing - Review & Editing, Supervision. Lorenz Waltl - Methodology, Investigation, Writing - Review & Editing. Guillaume Viault - Investigation. Danilo D’Avino - Investigation. Bill Perkowski - Investigation. Anita Siller - Resources, Writing - Review & Editing. Harald Schennach - Resources, Writing - Review & Editing, Supervision. Denis Séraphin - Supervision. Solveigh C. Koeberle - Investigation, Visualization, Writing - Review & Editing, Funding acquisition. Paul M Jordan - Methodology, Investigation Writing - Review & Editing. Fiorentina Roviezzo - Investigation. Pascal Richomme - Resources, Supervision. Antonietta Rossi - Investigation, Writing - Review & Editing, Supervision. Jean-Jacques Helesbeux - Resources, Investigation, Writing - Review & Editing, Supervision, Funding acquisition. Oliver Werz: Project administration, Conceptualization, Data Curation, Writing - Review & Editing, Supervision, Funding acquisition. Andreas Koeberle: Project administration, Conceptualization, Data Curation, Writing - Original Draft, Writing - Review & Editing, Supervision, Funding acquisition.

## Declaration of AI-assisted technologies

During the preparation of this work the authors used DeepL and ChatGPT for grammatical checks and to improve the sentence structure. After using these tools, the authors reviewed and edited the content as needed and take full responsibility for the content of the publication.

## Conflicts of interest

Pascal Richomme, Jean-Jacques Helesbeux, Oliver Werz and Andreas Koeberle are inventors of a patent related to the pharmacological use of tocotrienol derivatives in 5-lipoxygenase-related diseases.

## Appendix A Supporting information

The following are the Supporting Information to this article:
