# Supplementary material for: Tocotrienol metabolites redirect lipid mediator production in innate immune cells towards inflammation resolution
Source: Acta Pharm Sin B. 2026 Jul 1;16(9):6099–128. doi: 10.1016/j.apsb.2026.06.056 (PMC13589943; doi:10.1016/j.apsb.2026.06.056)
Supplement: Supplementary file 1 [file mmc1.pdf]

Supporting Information for

Original article

Tocotrienol metabolites redirect lipid mediator production in innate immune cells towards inflammation resolution

Stephan Permann[a,†], Elena Brunner[b,†], Khaled Alsabil[c], Markus Werner[b], Lorenz Waltl[a,d], Guillaume Viault[c], Danilo D'Avino[e], Bill Perkowski[b], Anita Siller[f], Laura Fritsch[b], Sara Perna[e], Harald Schennach[f], Denis Séraphin[c], Solveigh C. Koeberle[g], Paul M. Jordan[b], Fiorentina Roviezzo[e], Pascal Richomme[c], Antionetta Rossi[e], Jean-Jacques Helesbeux[c], Oliver Werz[b,*], Andreas Koeberle[a,g,*]

[a]*Michael Popp Institute and Center for Molecular Biosciences Innsbruck (CMBI), University of Innsbruck, Innsbruck 6020, Austria*

[b]*Department of Pharmaceutical/Medicinal Chemistry, Institute of Pharmacy, Friedrich Schiller University Jena, Jena 07743, Germany*

[c]*University Angers, SONAS, SFR QUASAV, Angers 49000, France*

[d]*Institute of Human Genetics, Medical University of Innsbruck, Innsbruck 6020, Austria*

[e]*Department of Pharmacy, School of Medicine and Surgery, University of Naples Federico II, Naples 80131, Italy*

[f]*Central Institute for Blood Transfusion and Immunology, Tirol Kliniken GmbH, Innsbruck 6020, Austria*

[g]*Institute of Pharmaceutical Sciences and Excellence Field BioHealth, NAWI Graz, University of Graz, Graz 8010, Austria*

Received 10 June 2025; received in revised form 22 September 2025; accepted 27 October 2025

*Corresponding authors.

E-mail addresses: andreas.koeberle@uni-graz.at (Andreas Koeberle), oliver.werz@uni-jena.de (Oliver Werz).

†These authors made equal contributions to this work.

Semi(synthesis) of LCMs

α-T-13′-COOH (**1**), *δ*-T-13′-COOH, *α*-T-13′-OH and *δ*-T-13′-OH have been obtained through the hydrogenation of the *ω*-oxidized tocotrienol analogues with slight modifications of a previously reported method[1]. A two-step sequence (Mannich reaction followed by the reduction of Mannich bases) led to *α*-TE-13′-COOH (**2**) and *β*-TE-13′-COOH (**3**) starting from *δ*-TE-13′-COOH. The same strategy applied to *δ*-TE-12a′,13′-diCH_2OH allowed the access to *α*-TE-12a′,13′-diCH_2OH and *β*-TE-12a′,13′-diCH_2OH[2]. Different semisynthetic approaches have also been developed to produce *δ*-TE-12a′-COOH (**5**), *β*-DE-13′-COOH (**6**), *α*-T-11′-COOH and *δ*-TE-11′-COOH[3].

General procedure for *α*- and *β*-TE-13′-CH_2OH synthesis from TE-13′-COOH isoforms: A suspension of lithium aluminum hydride (2 equiv.) in tetrahydrofuran (0.1 M) at 0 °C was combined with a solution of either *α*- or *β*-TE-13′-COOH (1 equiv.) in tetrahydrofuran (0.04 M). After 1.5 h at room temperature, the reaction was quenched with two drops of an aqueous solution of sodium hydroxide (1 M) and then with water. The resulting mixture was stirred for 15 min, then extracted three times with diethyl ether. The combined organic layers were washed with a saturated aqueous solution of Rochelle's salt, dried over anhydrous sodium sulfate, filtered and concentrated under reduced pressure. The residue was purified by column chromatography on silica gel eluted with a petroleum ether/acetone mixture 9:1 to afford the desired products as oils.

α-TE-13′-CH_2OH ((*R*)-2-((3*E*,7*E*,11*E*)-13-hydroxy-4,8,12-trimethyltrideca-3,7,11-trien-1-yl)-2,5,7,8-tetramethylchroman-6-ol): Pale-yellow oil; 49% yield; $R_f = 0.39$ (petroleum ether/acetone 8:2). ^{1}H NMR (400 MHz, $CDCl_3$) δ 5.38 (ddq, J = 1.3, 5.6, 8.3 Hz, 1H), 5.12 (dd, J = 7.1, 15.8 Hz, 2H), 3.99 (s, 2H), 2.61 (t, J = 6.9 Hz, 2H), 2.18–2.04 (m, 6H), 2.12 (s, 3H), 2.11 (s, 3H), 2.08 (s, 3H), 2.01–1.95 (m, 4H), 1.85–1.75 (m, 2H), 1.66 (s, 3H), 1.65–1.51 (m, 2H), 1.59 (s, 6H), 1.25 (s, 3H). ^{13}C NMR (100 MHz, $CDCl_3$) δ 145.6, 144.7, 135.1, 134.8, 134.7, 126.3, 124.6 (2C), 122.8, 121.2, 118.6, 117.4, 74.4, 69.2, 39.8, 39.6, 39.4, 31.7, 26.7, 26.4, 23.9, 22.4, 20.9, 16.1, 16.0, 13.8, 12.4, 11.9, 11.1. IR (neat) ν_{max} 3376, 2921, 2854, 1450, 1377, 1253, 1164, 1085, 1003, 924, 856 cm^{-1}. HRMS (FAB) *m/z* calcd for $C_{29}H_{44}O_3$ [$M^{+\cdot}$] 440.3290 found 440.3295.

β-TE-13′-CH_2OH ((*R*)-2-((3*E*,7*E*,11*E*)-13-hydroxy-4,8,12-trimethyltrideca-3,7,11-trien-1-yl)-2,5,8-trimethylchroman-6-ol): Pale-yellow oil; 34% yield; $R_f = 0.32$ (petroleum ether/acetone 8:2). ^{1}H NMR (400 MHz, $CDCl_3$) δ 6.48 (s, 1H), 5.38 (ddq, J = 1.3, 5.6, 8.3 Hz, 1H), 5.14-5.08 (m, 2H), 3.99 (s, 2H), 2.61 (t, J = 6.9 Hz, 2H), 2.14-2.03 (m, 6H), 2.11 (s, 3H), 2.08 (s, 3H), 2.01–1.95 (m, 4H), 1.87–1.74 (m, 2H), 1.66 (s, 3H), 1.64–1.46 (m, 2H), 1.59 (s, 6H), 1.25 (s, 3H). ^{13}C NMR (100 MHz, $CDCl_3$) δ 146.0, 145.9, 135.1, 134.8, 134.7, 126.4, 126.4 (2C), 124.2, 120.4, 119.3, 115.4, 74.4, 69.2, 39.8, 39.4 (2C), 31.6, 26.7, 26.4, 24.0, 22.3, 20.9, 16.7, 16.1, 16.0 (2C), 13.8, 11.1. IR (neat) ν_{max} 3357, 2970, 2922, 2854, 1458, 1415, 1230, 1164, 1098, 1061, 1004 cm^{-1}. HRMS (FAB) *m/z* calcd for $C_{28}H_{42}O_3$ [$M^{+\cdot}$] 426.3434 found 426.3431.

Table S1 Effects of endogenous metabolites and derivatives on 15-HETE formation in activated PMNL.

Abbreviation	Structure	15-HETE [% of vehicle control][a]
α-T	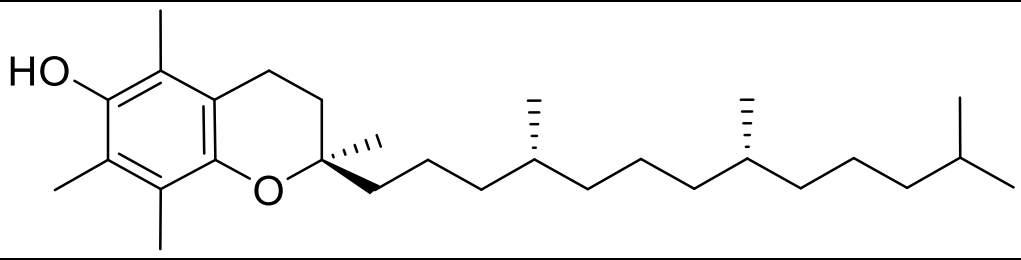	103.64 ± 12.96[4]
α-TE	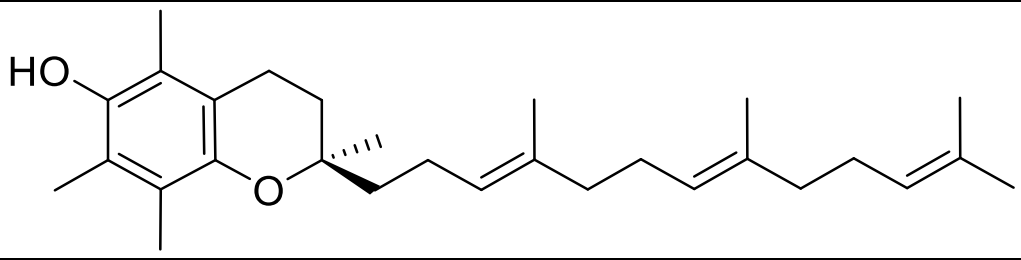	98.58 ± 3.85[4]
β-T	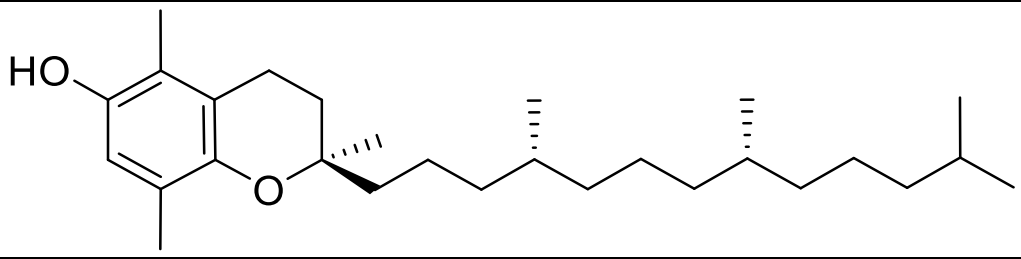	98.63 ± 2.83[4]
β-TE	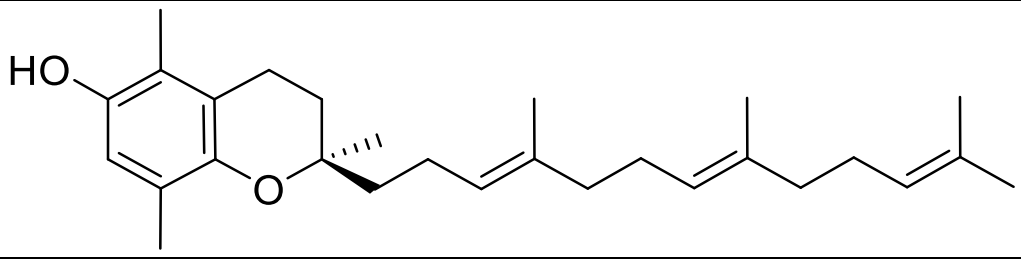	96.89 ± 7.46[4]
γ-T	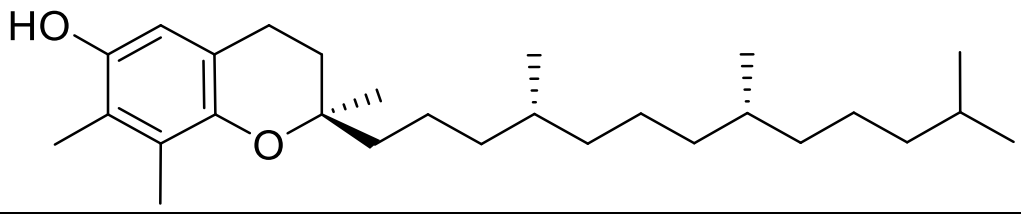	94.79 ± 10.99[4]
γ-TE	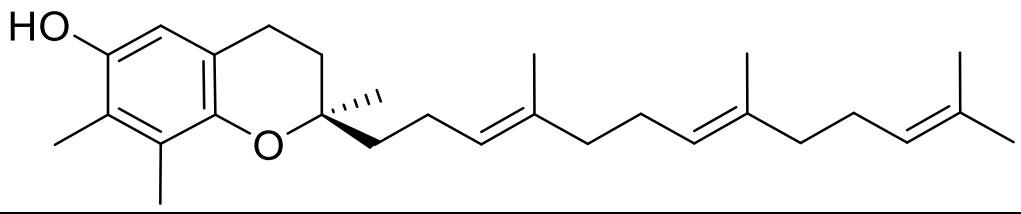	99.29 ± 7.81[4]
δ-T	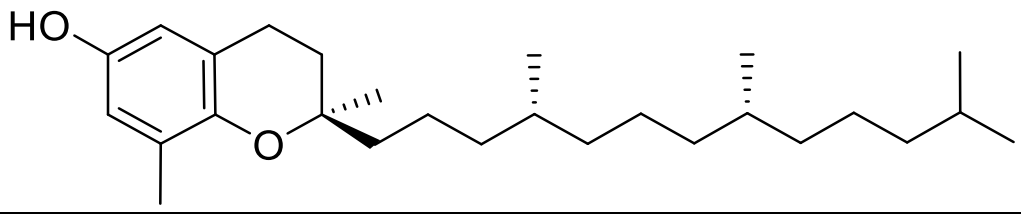	86.63 ± 15.79[4]
δ-TE	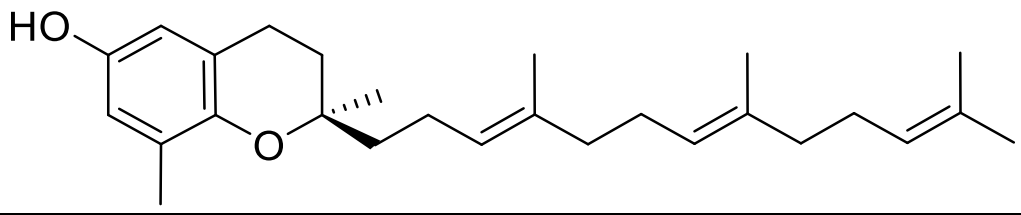	98.83 ± 20.29[4]
α-T-13′-COOH (**1**)	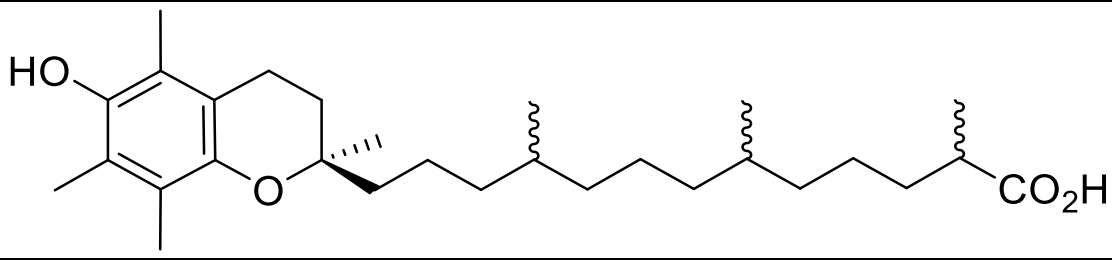	103.4 ± 20.98
α-TE-13′-COOH (**2**)	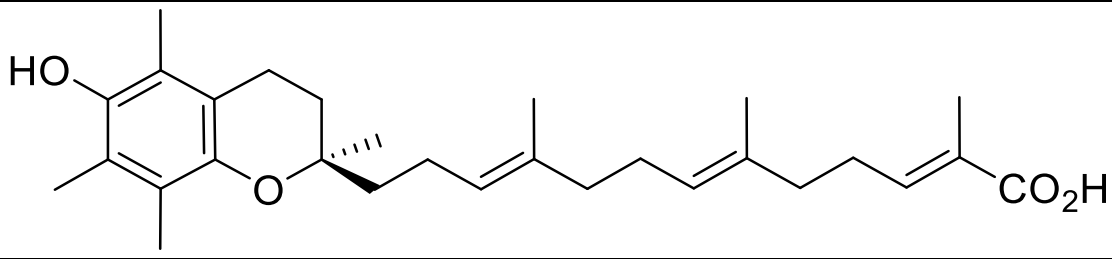	209.32 ± 35.21
β-TE-13′-COOH (**3**)	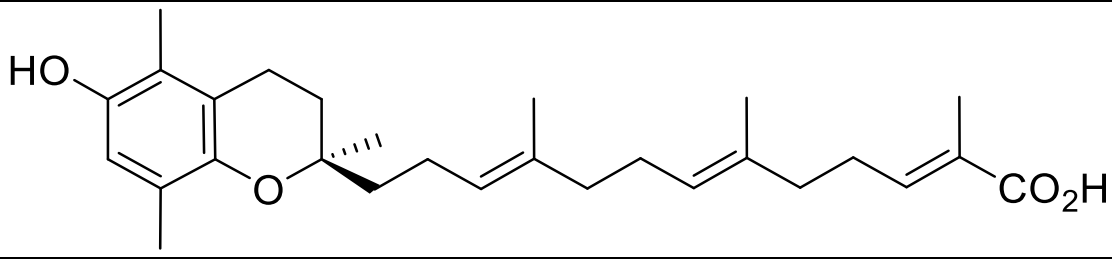	213.06 ± 39.35
γ-TE-13′-COOH	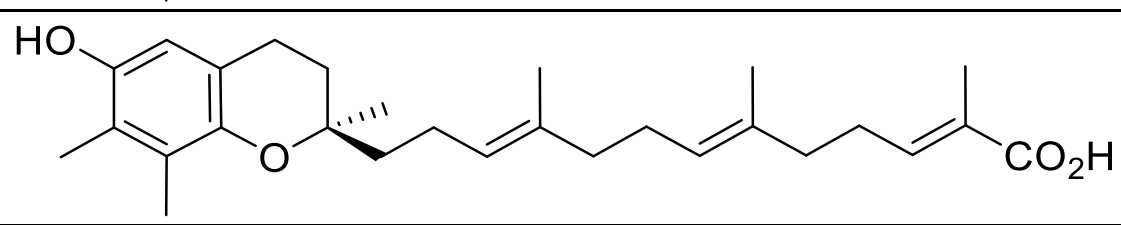	169.23 ± 4.48[4]
δ-T-13′-COOH	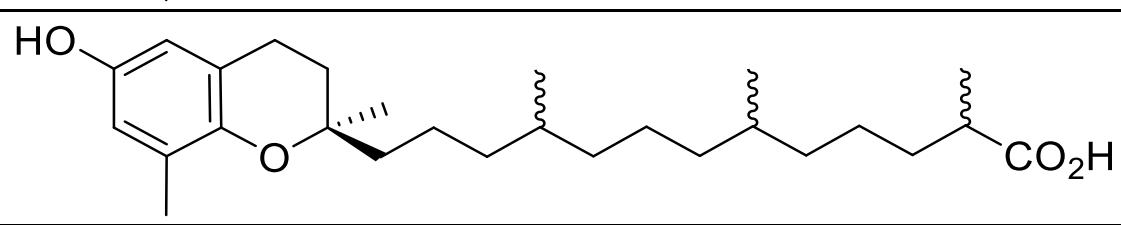	121.62 ± 10.85[4]
δ-TE-13′-COOH (**4**)	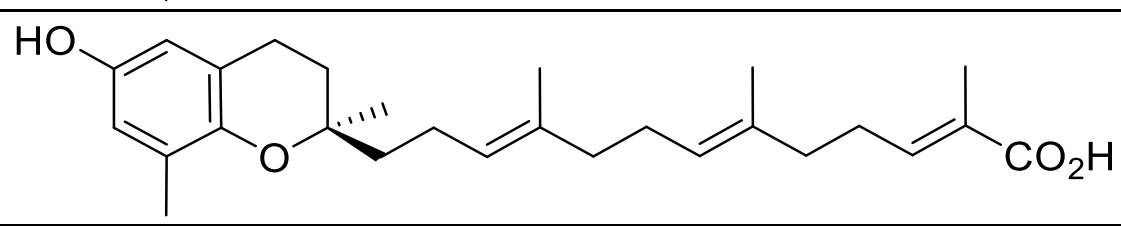	95.61 ± 44.51

α-T-13′-CH$_2$OH	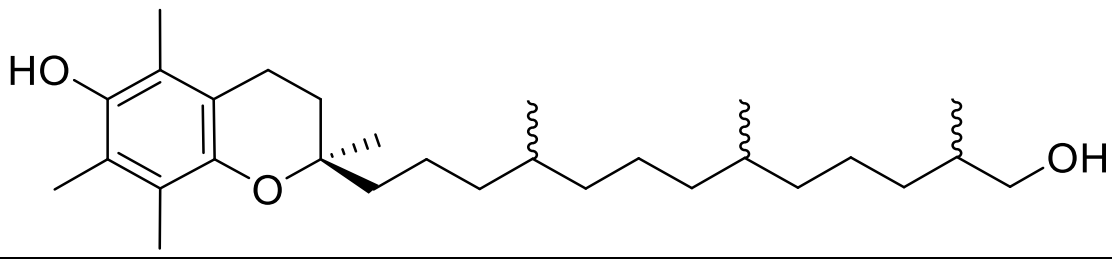	156.69 ± 26.18[4]
α-TE-13′-CH$_2$OH	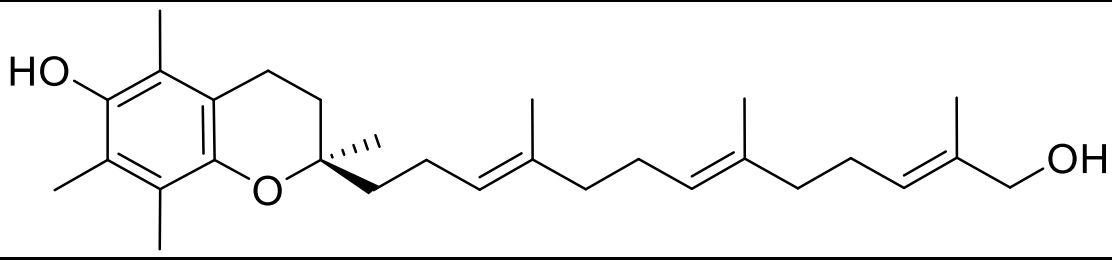	107.11 ± 25.76
β-TE-13′-CH$_2$OH	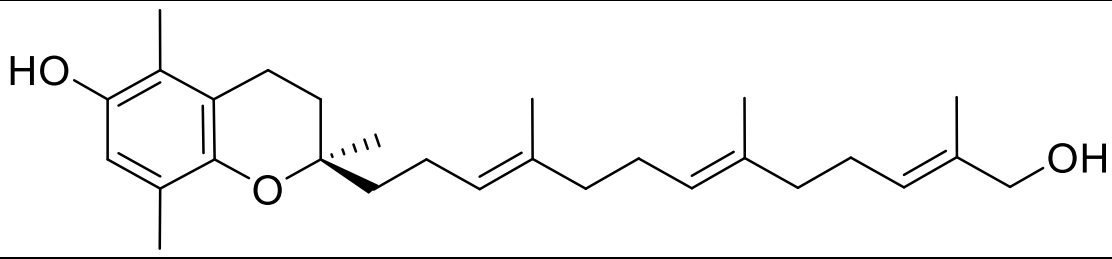	96.45 ± 23.26
γ-TE-12a′-CH$_2$OH (**8**)	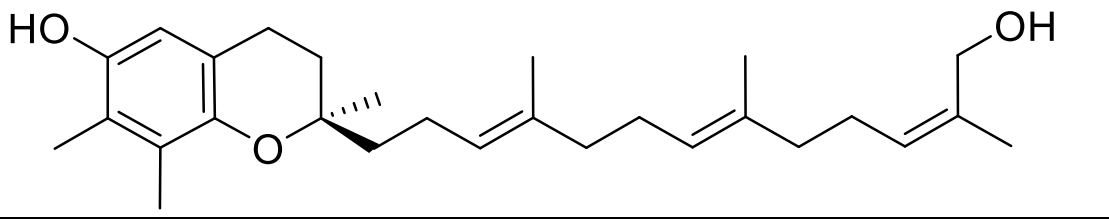	168.76 ± 1.21[4]
δ-T-13′-CH$_2$OH	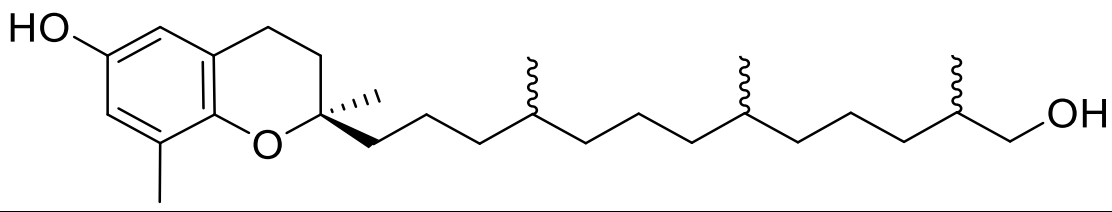	142.78 ± 13.90[4]
δ-TE-13′-CH$_2$OH	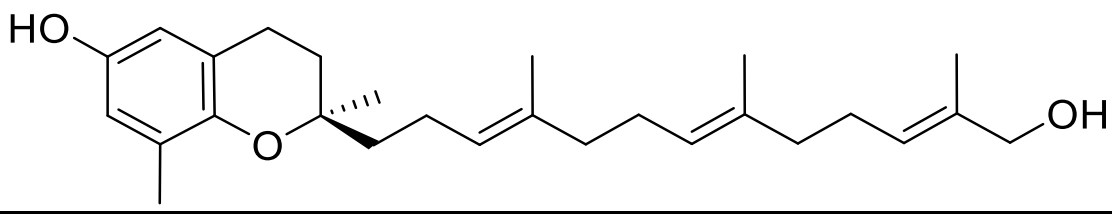	128.45 ± 59.46[4]
δ-TE-12a′-CH$_2$OH (**7**)	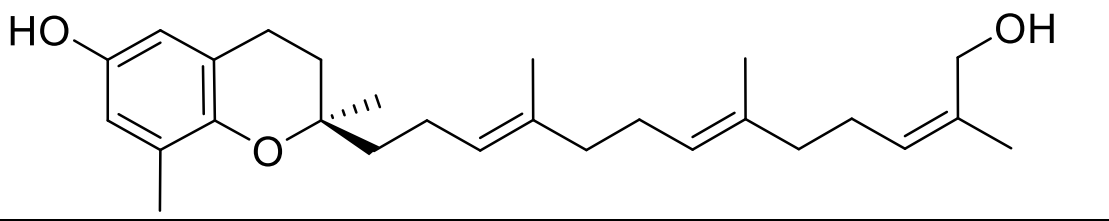	174.64 ± 6.91[4]
δ-TE-12a′-COOH (**5**)	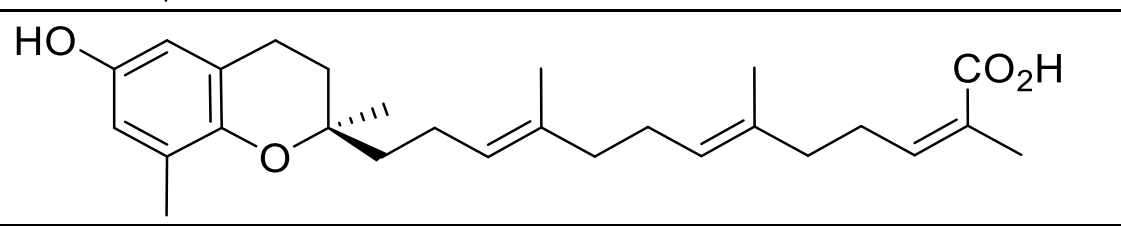	266.24 ± 49.46
β-DE-13′-COOH (**6**)	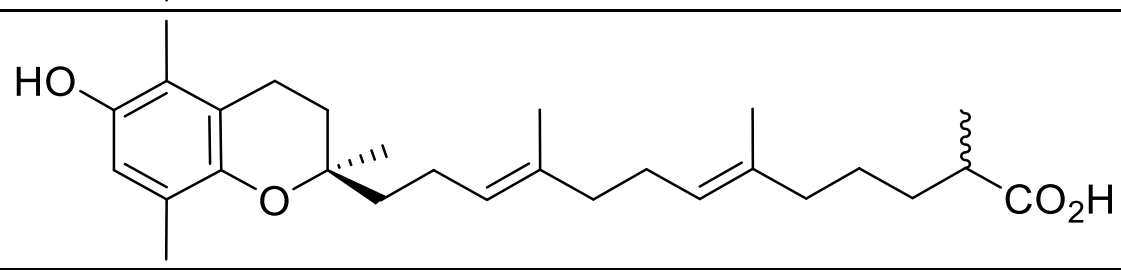	249.11 ± 41.56
α-TE-12a′,13′-diCH$_2$OH	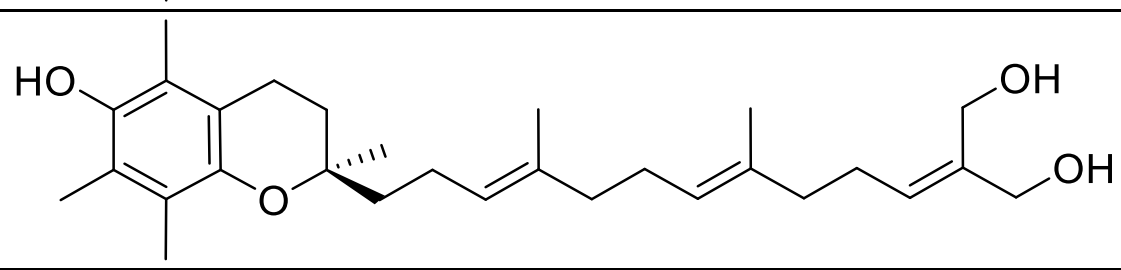	116.59 ± 24.25[3]
β-TE-12a′,13′-diCH$_2$OH	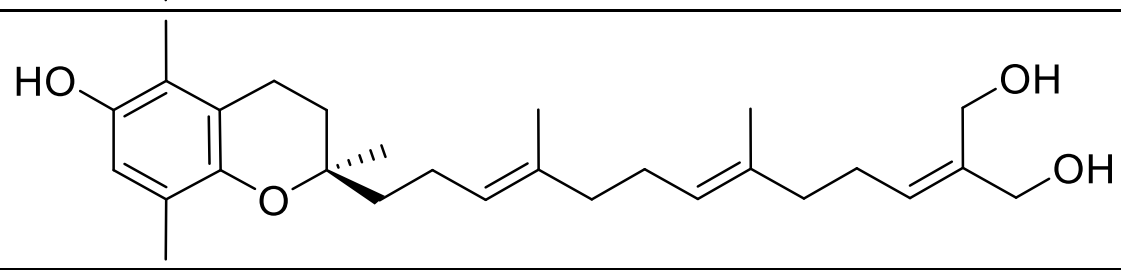	107.94 ± 21.52
γ-TE-12a′,13′-diCH$_2$OH	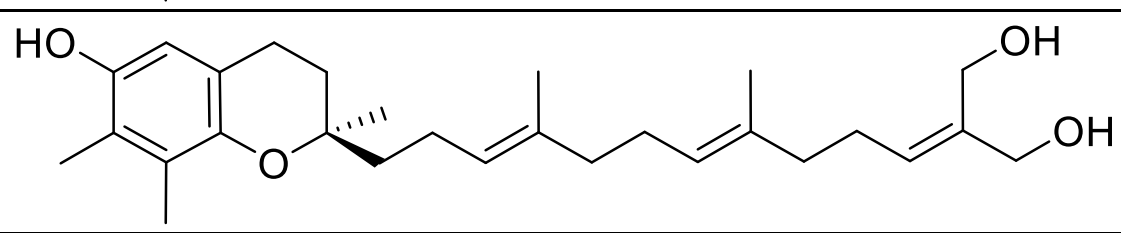	95.28 ± 14.92
δ-TE-12a′-CH$_2$OH-13′-CHO	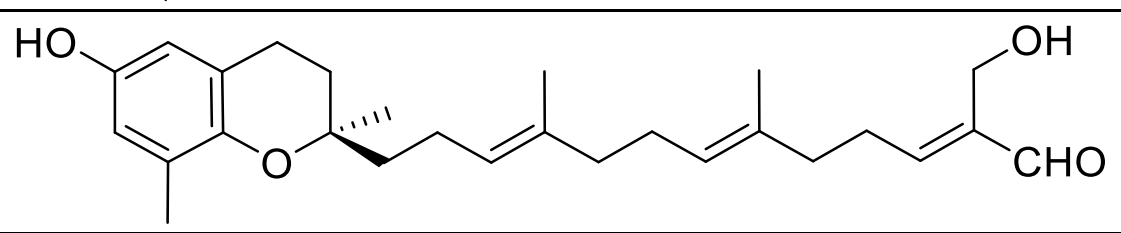	159.50 ± 8.20
α-T-11′-COOH	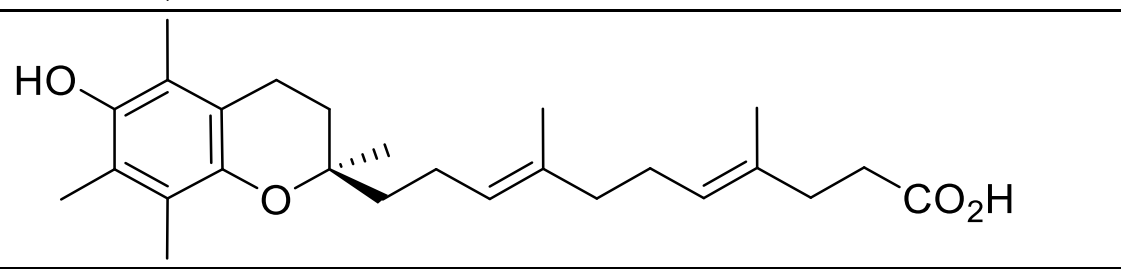	202.79 ± 69.48
δ-TE-11′-COOH	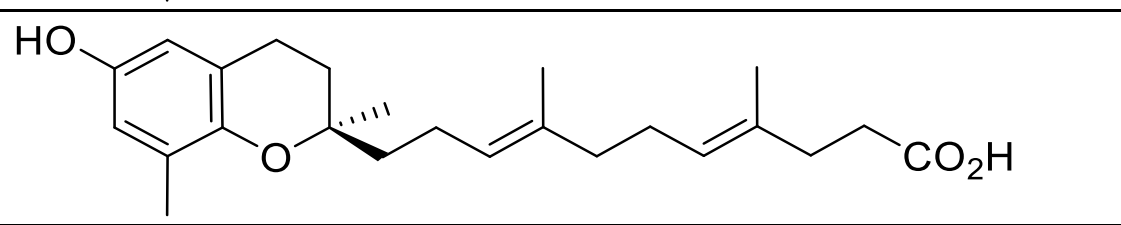	151.65 ± 55.20

[a]at 3 µmol/L compound concentration (*n* = 3 independent experiments, mean ± SEM).
[b]not determined.

Table S2 Stimulation of lipid mediator formation in M2 macrophages with SACM.

Amount (pg / 2 × 10^6 cells)						
	w/o			+ SACM		
	Mean	±	SEM	Mean	±	SEM
PD1/iso	0.39	±	0.09	61.61	±	13.87
AT-PD1/iso	69.63	±	65.89	314.75	±	83.47
PDX/iso	5.20	±	3.36	1930.54	±	437.89
RvD5/iso	12.46	±	1.84	5384.33	±	1120.70
MaR2/iso	0.00	±	0.00	359.30	±	134.24
RvD1/iso	0.79	±	0.46	110.27	±	18.95
RvD2/iso	6.89	±	1.90	898.95	±	241.92
RvD5 $_{n3\text{-}DPA}$/iso	1.13	±	0.41	767.88	±	411.19
LXA4/iso	3.74	±	1.89	396.38	±	211.82
17-HDHA	179.49	±	35.50	34534.75	±	4121.50
17-HDPA	212.05	±	31.35	38625.88	±	4892.11
15-HETE	461.98	±	132.58	67846.97	±	22290.12
14-HDHA	37.12	±	6.29	7493.23	±	1510.57
18-HEPE	5.95	±	1.56	469.09	±	129.71
15-HEPE	35.85	±	10.14	8978.34	±	3317.65
14-HDPA	67.40	±	11.17	12905.99	±	2535.70
12-HEPE	6.45	±	1.75	1593.26	±	563.05
12-HETE	73.70	±	23.29	13768.24	±	5544.51
LTB_4 isomers	24.48	±	13.77	14585.02	±	8551.47
LTB_4	6.18	±	3.86	5074.40	±	3449.78
5-HEPE	1.27	±	0.38	256.86	±	143.43
5-HETE	12.36	±	3.24	2051.12	±	1198.38
AA	24921.00	±	4500.93	899497.09	±	129717.75
EPA	3500.98	±	867.37	130926.71	±	19570.87
DHA	19336.11	±	3897.86	716725.81	±	108212.01
DPA	370.42	±	65.89	15854.76	±	3047.75
PGE_2	24.78	±	2.63	958.94	±	362.76
15-ketoPGE_2	17.81	±	3.40	689.14	±	196.39
PGD_2	18.95	±	3.52	367.29	±	109.80
$PGF_{2\alpha}$	43.88	±	11.45	675.25	±	150.43
TXB_2	626.18	±	138.75	19809.09	±	4054.80
PGE_1/PGD_1	2.47	±	0.37	44.17	±	9.47
5.6-EET	3.76	±	0.87	135.60	±	64.88
11.12-EET	5.23	±	2.01	208.01	±	70.52
14.15-EET	11.16	±	4.86	497.46	±	209.43
13-HDHA	8.22	±	3.04	1719.98	±	198.55
7-HDHA	14.17	±	2.83	2395.64	±	418.07
4-HDHA	6.70	±	2.14	357.34	±	65.50
7-HDPA	9.19	±	2.58	1351.32	±	668.73
11-HEPE	0.62	±	0.26	165.91	±	48.27
11-HETE	10.27	±	2.02	1639.47	±	405.72
8-HETE	47.90	±	22.96	15323.82	±	6022.19
9-HODE	380.67	±	85.69	2801.29	±	360.75
13-HODE	973.54	±	191.56	69279.02	±	14765.94
12-HHT	33.78	±	12.84	9695.14	±	1830.56
5,15-diHETE	25.18	±	8.38	12421.23	±	5892.34
5,15-diHEPE	7.86	±	3.43	805.07	±	400.51

Table S8 DHA autooxidation compared to its conversion to lipid mediators by M2-like macrophages.

[pg][a]	DHA[b]			DHA + M2[c]			DHA + M2 + SACM[d]		
4-HDHA	5612.99	±	89.73	1409.86	±	880.70	9844.12	±	4879.70
7-HDHA	2902.20	±	75.44	2992.45	±	1749.04	6684.64	±	2194.13
14-HDHA	6764.39	±	457.34	2590.55	±	1662.41	32937.69	±	6277.24
17-HDHA	14809.64	±	1074.05	6924.04	±	4102.64	108347.97	±	13629.19
PDX/iso	533.95	±	36.34	1185.40	±	455.03	3067.86	±	388.19
MaR2/iso	494.78	±	51.30	72.01	±	20.58	191.75	±	21.62
PD1/iso	19.24	±	2.97	17.60	±	5.35	73.49	±	22.85
AT-PD1/iso	80.68	±	4.48	383.89	±	58.42	700.67	±	96.43
RvD5/iso	117.87	±	4.75	823.55	±	398.63	8419.15	±	224.49
DHA	56476.43	±	3638.48	4162.60	±	2267.36	21751.22	±	7268.08

[a]Mean ± SEM of the absolute amounts of selected lipid mediators in pg, $n = 4$.
[b]20 µmol/L DHA (195 min, room temperature).
[c]20 µmol/L of DHA added to 2×10^6 M2 macrophages (195 min, 37 °C).
[d]20 µmol/L of DHA added to 2×10^6 M2 macrophages (15 min, 37 °C) and treated with 0.1% SACM (180 min, 37 °C).

Tables S3–S7, **S9, S10** See excel files.

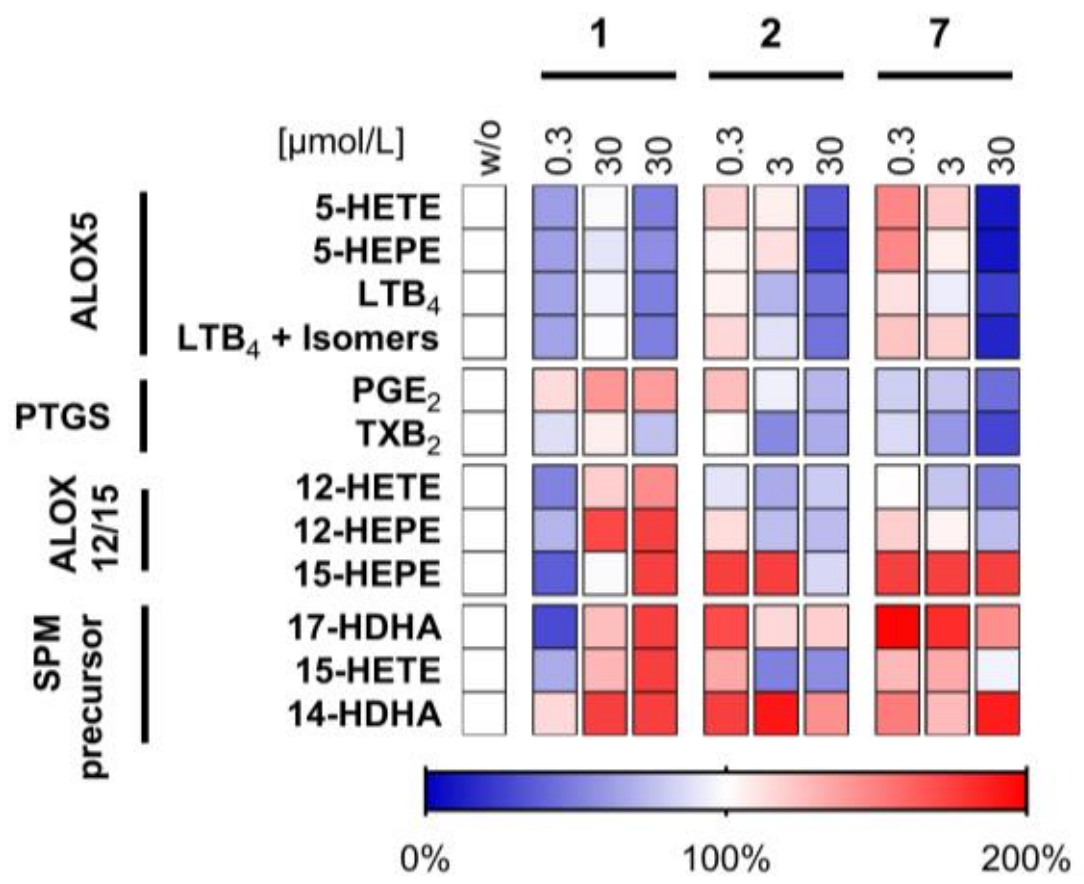

Figure S1 Effect of LCMs on PTGS- and ALOX-derived lipid mediators in LPS-primed human whole blood upon activation. Human whole blood (1 mL) was primed with LPS (100 ng/mL) for 20 h, preincubated with LCMs or vehicle (DMSO, 0.1%, 'w/o') for 15 min, and treated with A23187 (30 µmol/L) for 10 min. The heatmap shows changes in lipid mediator levels relative to the vehicle control. The labels ALOX5, PTGS and ALOX12/15 indicate products of these enzymes. Mean from n = 3 independent experiments.

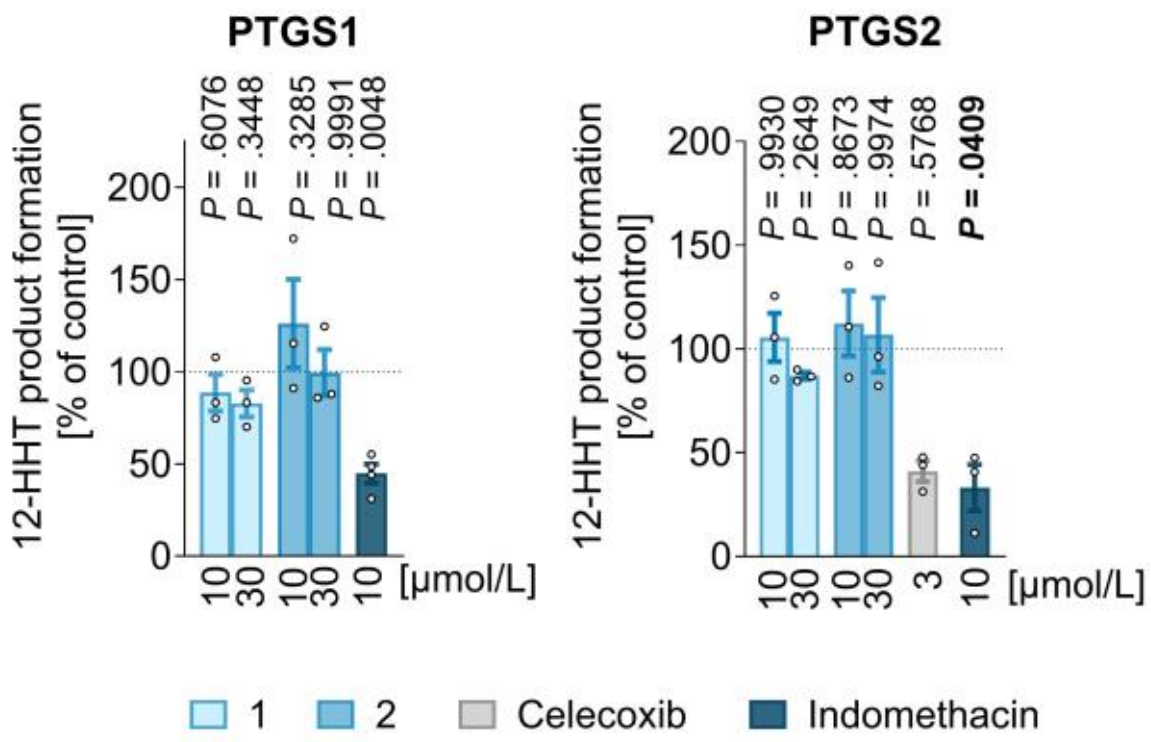

Figure S2 LCMs **1** and **2** do not inhibit cell-free PTGS activity. PTGS isoenzymes were preincubated with vehicle (DMSO, 0.1%), LCMs, celecoxib (3 µmol/L), or indomethacin (10 µmol/L) for 5 min at room temperature before PTGS-dependent 12-HHT formation was initiated by addition of AA. Mean ± SEM and single data from n = 3–4 independent experiments. *P* values *vs.* vehicle control; repeated measures one-way ANOVAs with Dunnett's *post hoc* tests.

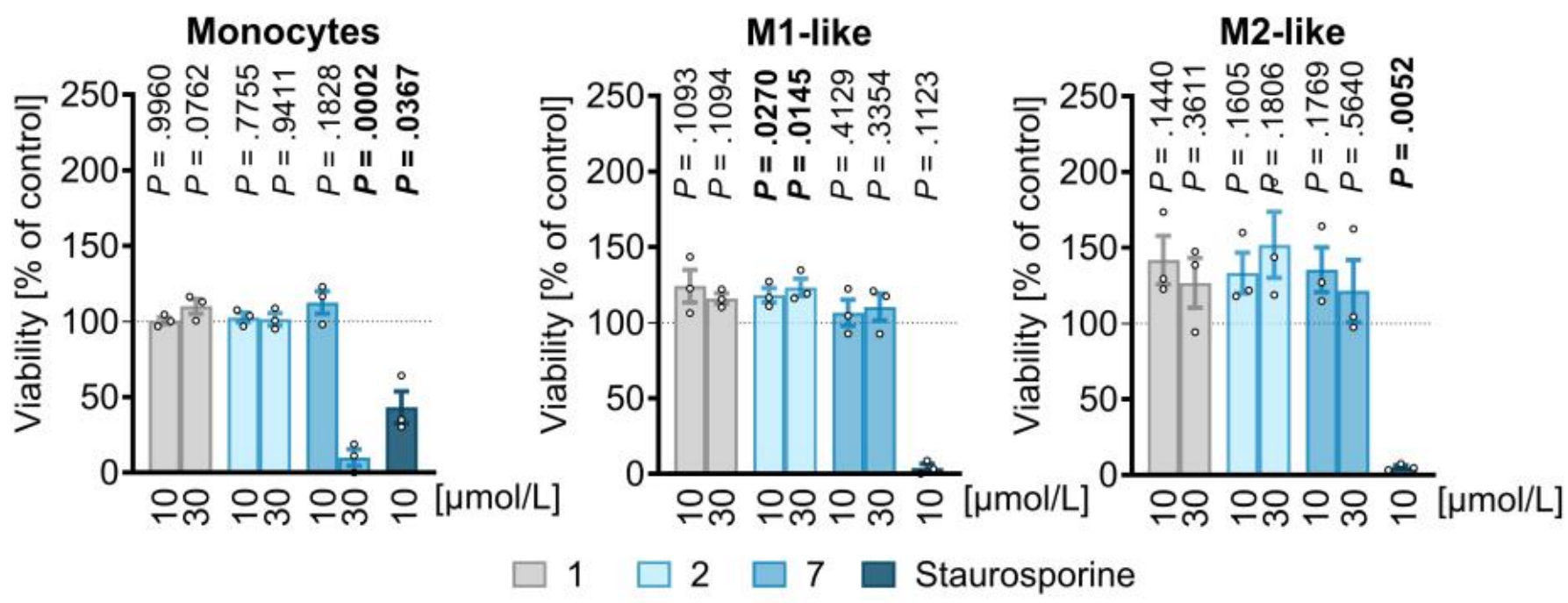

Figure S3 LCMs **1**, **2** and **7** do not decrease the viability of innate immune cells. Monocytes (1×10[5]), M1-like macrophages (1.5×10[5]) and M2-like macrophages (1.5×10[5]) were preincubated with vehicle (DMSO, 0.1%), LCMs, or staurosporine (10 µmol/L) for 48 h. Cell viability was assessed by measuring the conversion of MTT by cellular dehydrogenases. Mean ± SEM and single data from $n = 3$ independent experiments. *P* values *vs.* vehicle control; repeated measures one-way ANOVAs with Dunnett's *post hoc* tests (**1**, **2**, **7**) or paired two-sided Student *t*-tests (staurosporine).

A

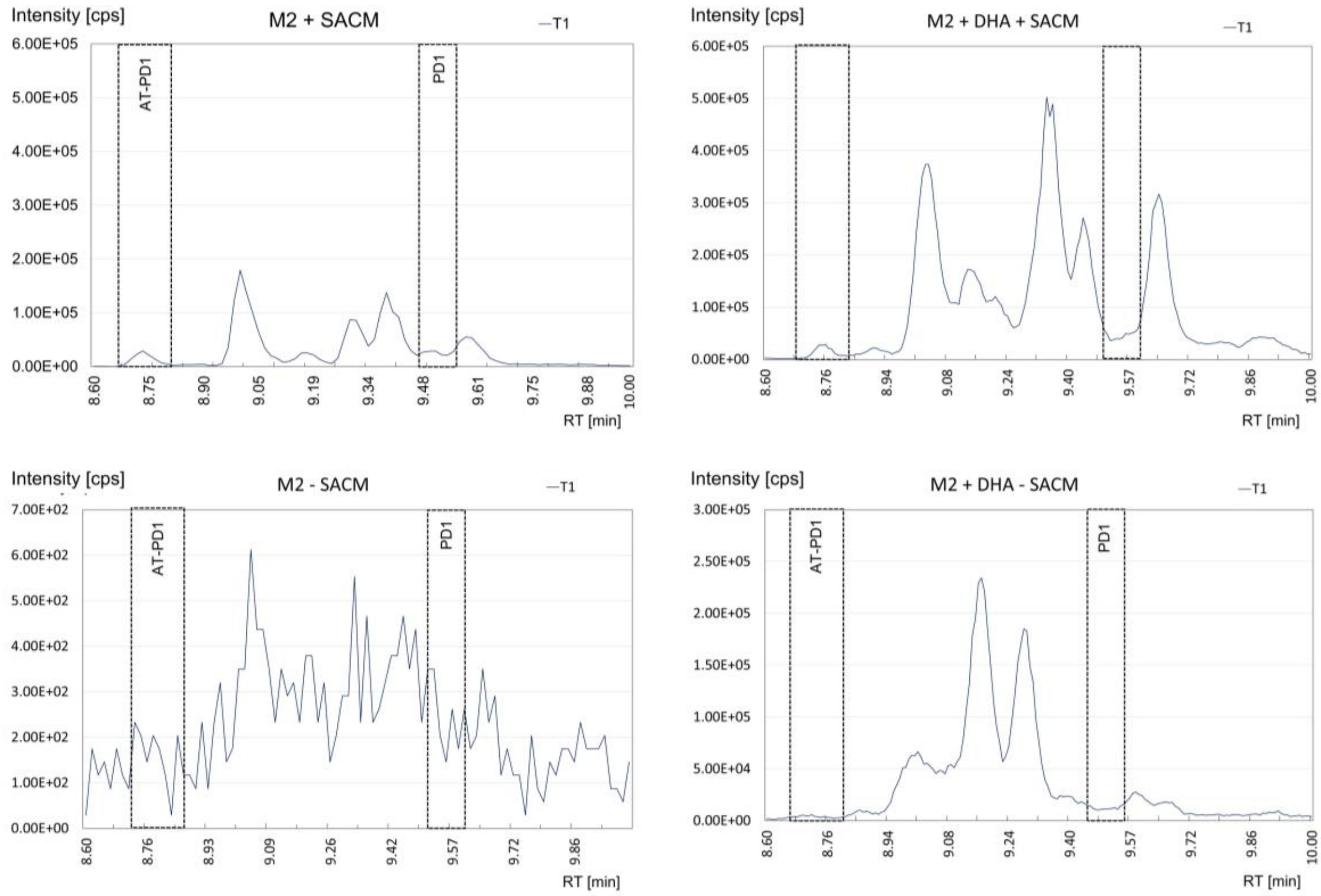

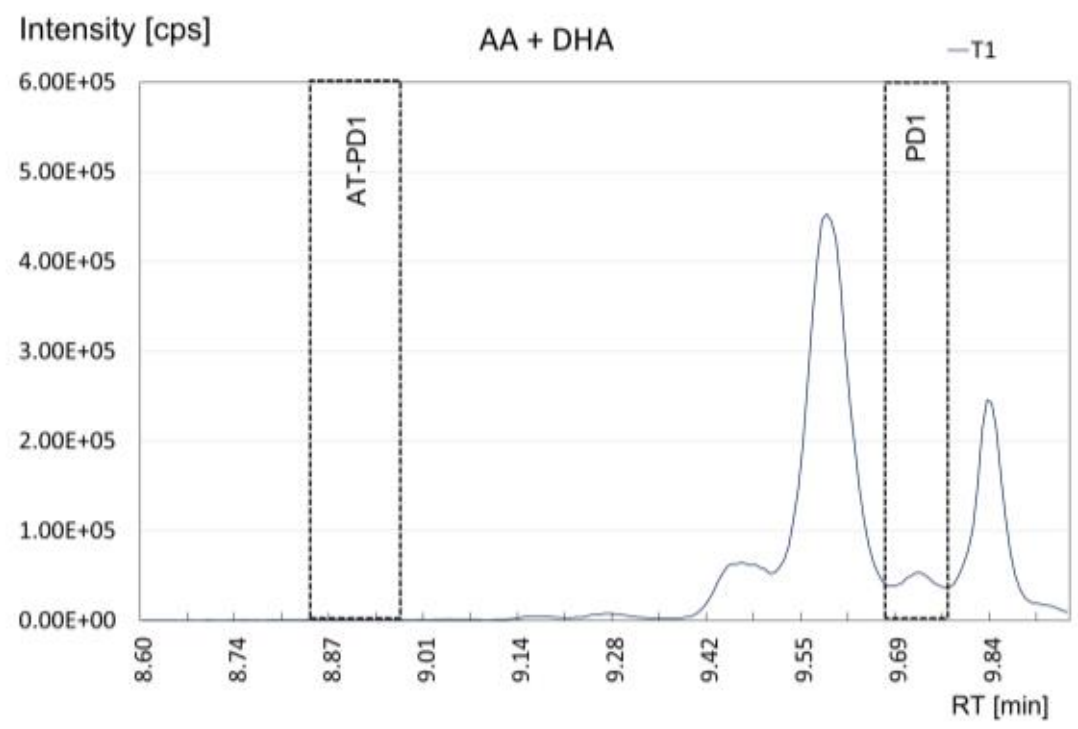

B

RT [min]	d4-LTB$_4$	AT-PD1	PDX	PD1
M2 + SACM	9.68	8.72	9.30	9.51
M2 - SACM	9.74	8.78	9.35	9.56
M2 + DHA + SACM	9.73	8.77	9.34	9.55
M2 + DHA - SACM	9.70	8.74	9.31	9.52
AA+ DHA	9.86	8.90	9.47	9.68

Figure S4 Autooxidation of exogenous PUFAs yields PD1 or isomers that interfere with PD1 quantitation. M2 macrophages (2×10^6) were preincubated with vehicle (DMSO, 0.1%) or DHA (20 µmol/L) for 15 min and then stimulated with SACM (0.1%) for 180 min or left unchallenged for this period. Alternatively, DHA (20 µmol/L) and AA (20 µmol/L) were incubated in the absence of M2 macrophages in PBS pH 7.4 at room temperature for 195 min. (A) Extracted chromatograms for PD1 and AT-PD1 (T1: 359.2 → 153.1; T2: 359.2 → 181.1). Retention time windows for PD1 and AT-PD1 were defined based on external standards and are indicated by boxes. (B) Retention times (RT) of AT-PD1, PDX, PD1 (according to batch controls with external standards) and the internal standard d_4-LTB$_4$.

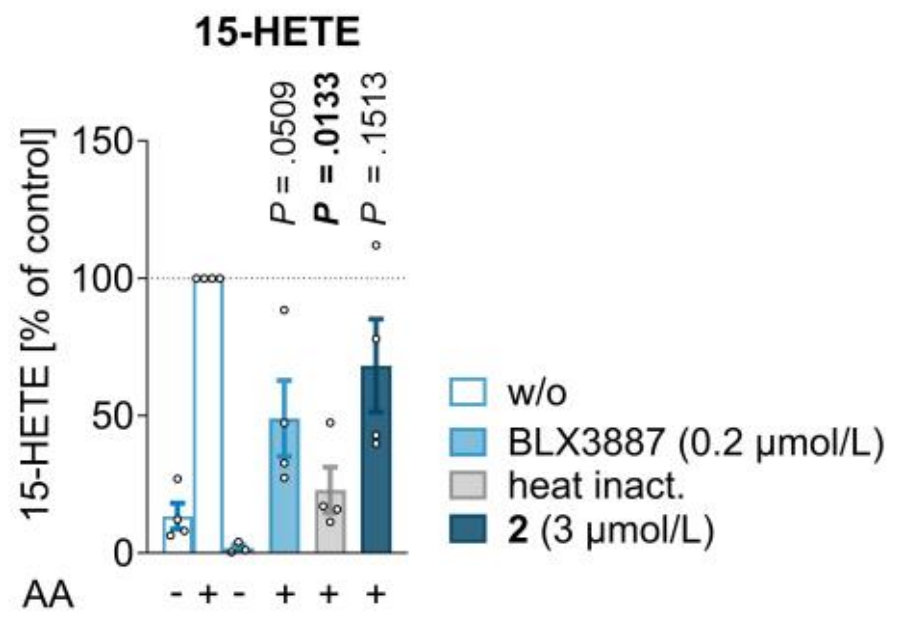

Figure S5 LCM **2** does not directly activate ALOX15. Homogenates of 2×10^{6} M2-like macrophages were incubated with vehicle (DMSO, 0.1%), **2** (3 µmol/L), or BLX3887 (0.2 µmol/L) for 15 min and then treated with AA (20 µmol/L) for 180 min or left unchallenged. Heat-inactivated homogenates ('heat-inact.') (95 °C, 5 min) were used as control. Data for controls are identical to Waltl et al.[5] Mean ± SEM and single data from n = 4 independent experiments. *P* values *vs.* the AA-treated vehicle control; paired two-sided Student *t*-tests.

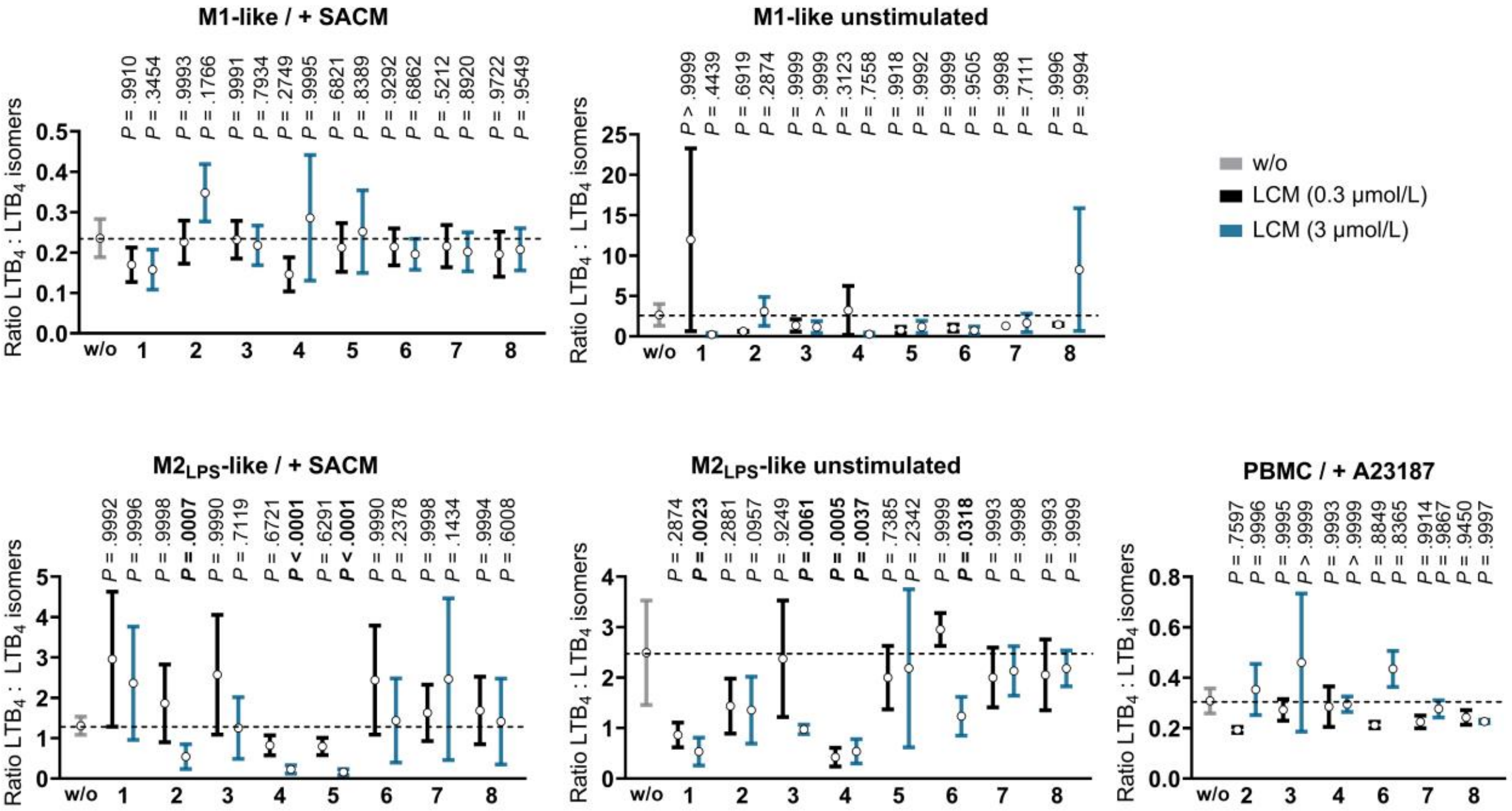

Figure S6 LCM-induced changes in the ratio of LTB_4 to LTB_4 isomers in macrophage subtypes. M1-like macrophages (2×10^6), $M2_{LPS}$-like macrophages (2×10^6), or PBMC (5×10^6) were preincubated with vehicle (DMSO, 0.1%, 'w/o') or LCM (0.3 or 3 µmol/L) for 15 min and then treated with SACM (1%) for 180 min or left unchallenged (M1, $M2_{LPS}$) or were treated with A23187 (2.5 µmol/L) for 10 min (PBMC). Mean ± SEM and single data from n = 3–4 independent experiments. P values *vs.* vehicle control; repeated measures one-way ANOVAs with Dunnett's *post hoc* tests of log data.

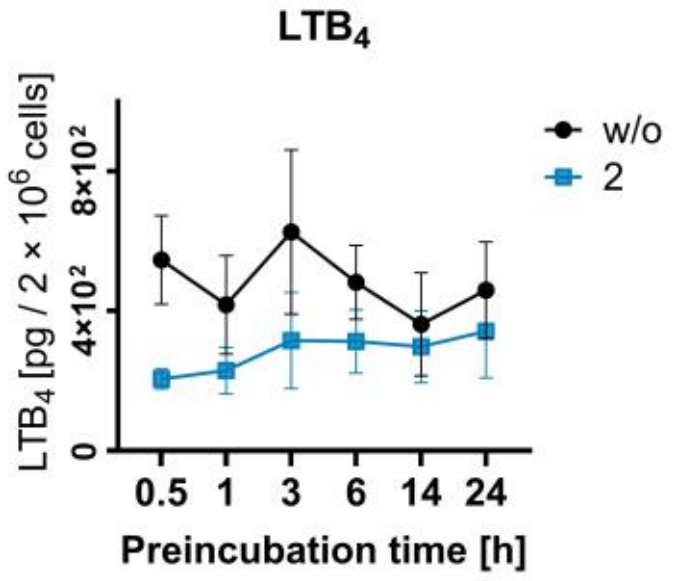

Figure S7. Influence of pre-incubation time with compound **2** on LTB_4 levels in activated M2-like macrophages. Cells (2×10^6) were preincubated with LCM **2** (3 µmol/L) or vehicle (DMSO, 0.1%, 'w/o') for 0.5 to 24 h before being stimulated with SACM (1 %) for 180 min. Time-dependent changes of LTB_4 for **2**- *vs.* vehicle-treated cells. Mean ± SEM and single data from $n = 3$ independent experiments. *P* values *vs.* the vehicle control at the respective time point; paired two-sided Student *t*-tests of log data.

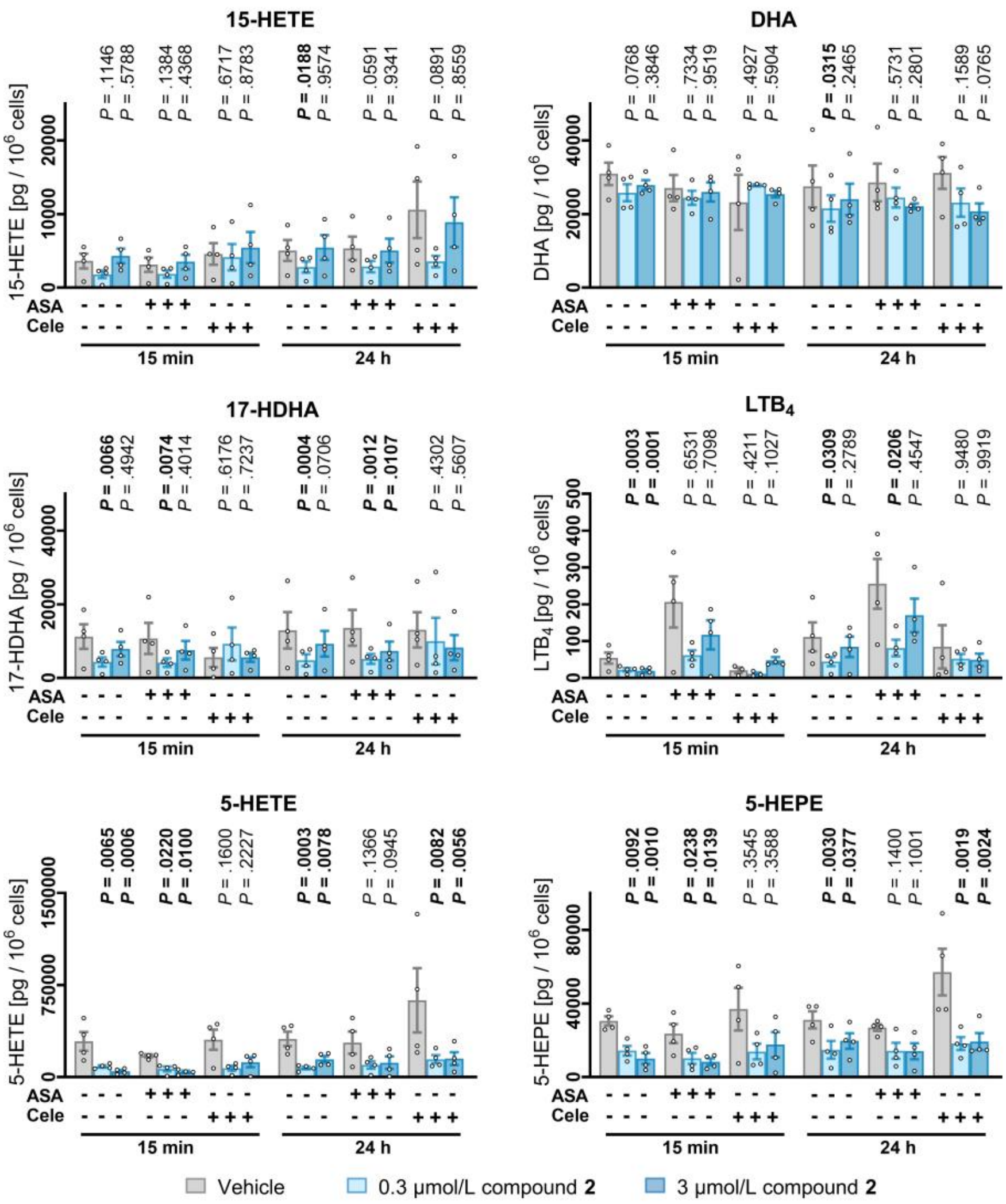

Figure S8 Effect of PTGS acetylation and isoenzyme-selective PTGS2 inhibition on 2-induced changes in DHA and lipid mediator levels in activated M2 macrophages. M2-like macrophages (2×10[6]) were preincubated with vehicle (DMSO, 0.1%), acetylsalicylic acid (ASA, 500 µmol/L), or celecoxib ('Cele', 3 µmol/L) for 15 min or 24 h, then exposed to vehicle (DMSO, 0.1%, 'w/o') or LCMs (3 µmol/L) for 15 min, and stimulated with SACM (1%) for 180 min. Levels of 15-HETE, DHA, 17-HDHA, LTB_4, 5-HETE, and 5-HEPE. Mean ± SEM and single data from *n* = 3–4 independent experiments. *P* values *vs.* vehicle-treated cells in the presence or the absence of a PTGS inhibitor; repeated measures one-way ANOVAs with Dunnett's *post hoc* tests.

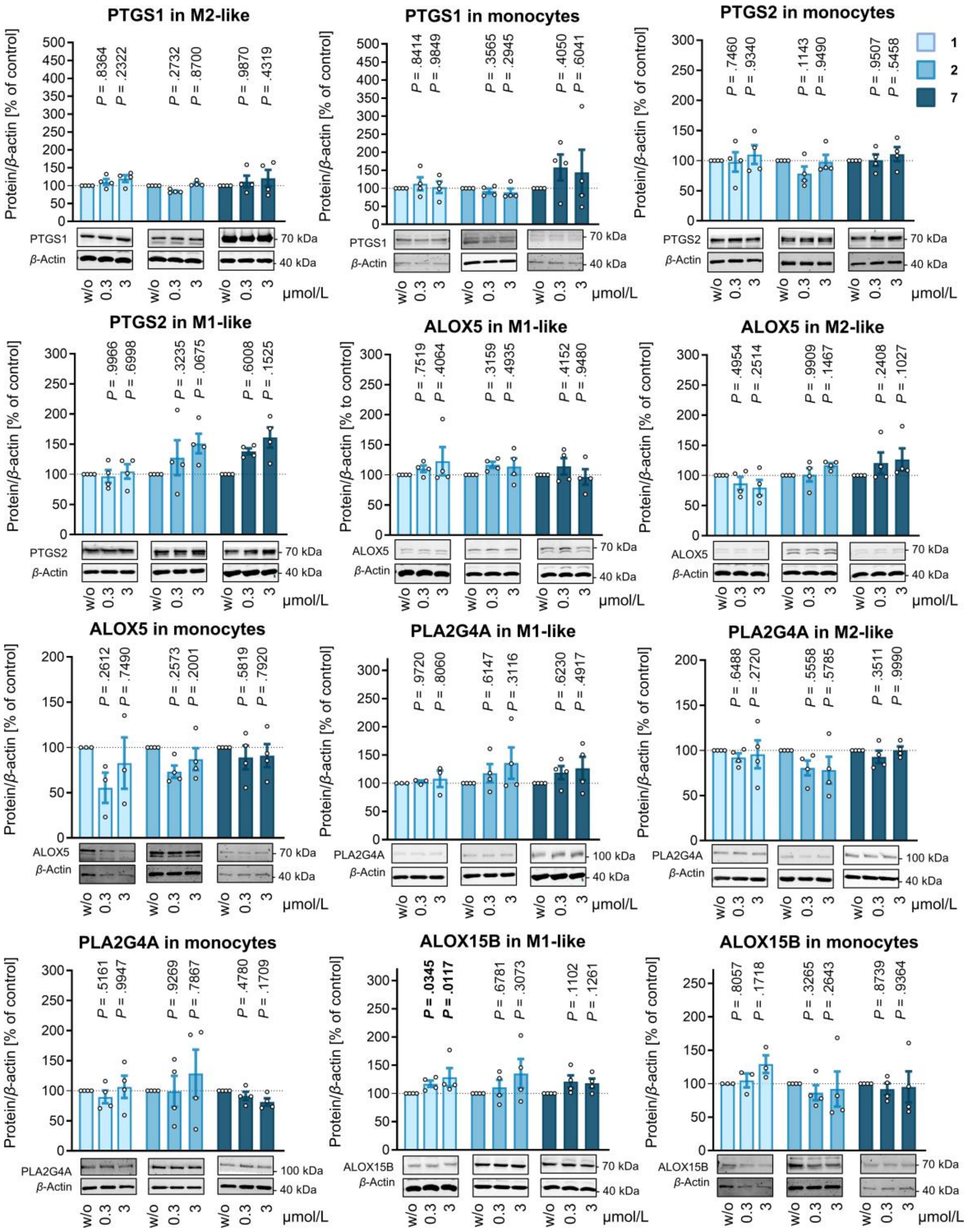

Figure S9 Effect of LCMs **1**, **2** and **7** on the protein expression of enzymes in lipid mediator biosynthesis. Protein expression of PTGS1, PTGS2, ALOX5, PLA2G4A, and ALOX15B in monocytes (3×10^6), M1-like macrophages (2×10^6), or M2-like macrophages (2×10^6) preincubated with vehicle (DMSO, 0.1%, 'w/o') or LCMs for 15 min and polarized to M1-like macrophages with interferon-γ and LPS or to M2-like macrophages with IL-4. β-Actin blots are shown repeatedly as loading controls for proteins detected on the same membrane, as documented in Supporting Information Figs. S15–S20. Mean ± SEM and single data from n = 3–4 independent experiments. P values *vs.* vehicle-treated cells; repeated measures one-way ANOVAs with Dunnett's *post hoc* tests.

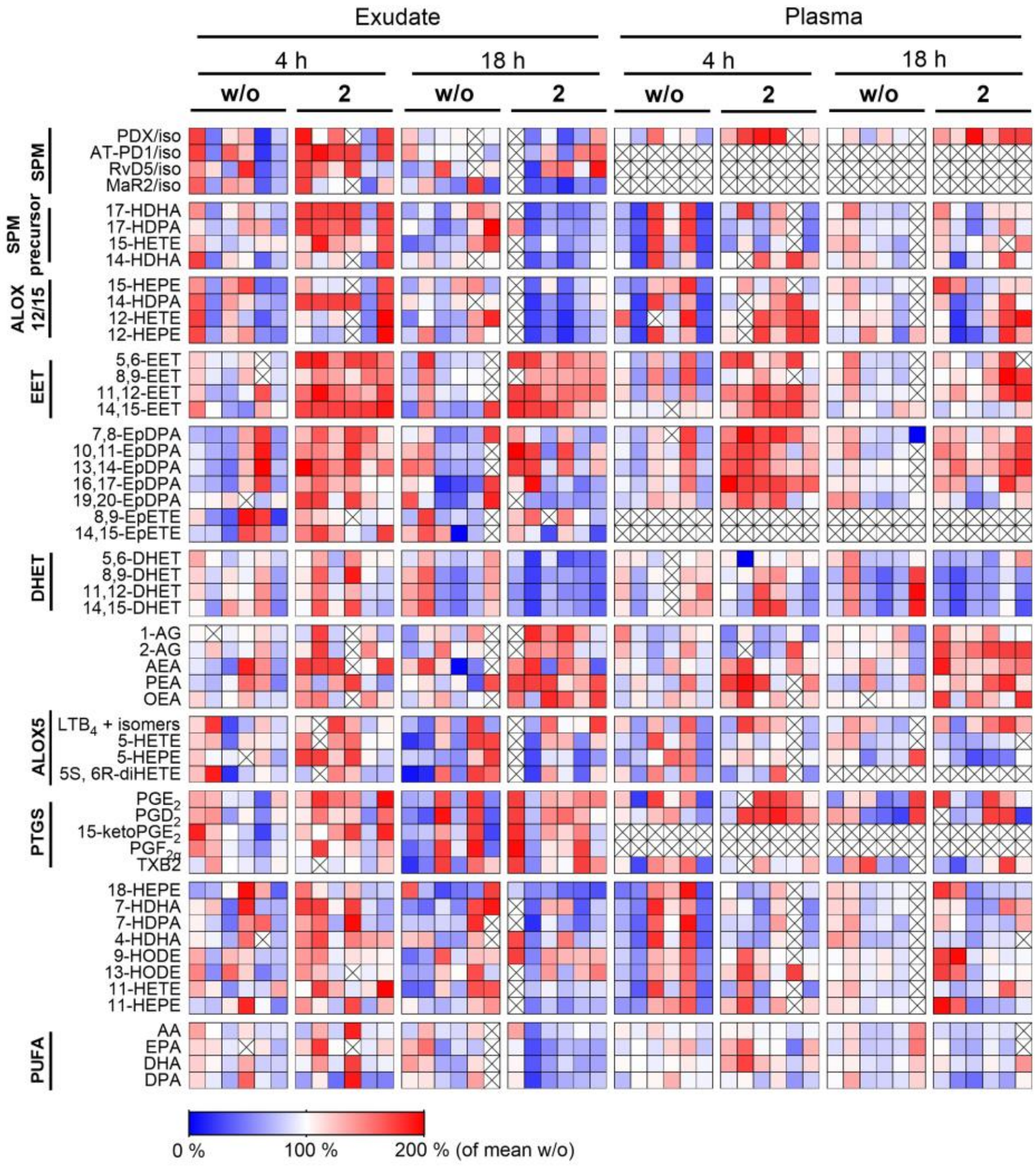

Figure S10 Effect of LCM **2** on the lipid mediator profile in experimental peritonitis. Mice received vehicle (2% DMSO, 'w/o') or **2** (10 mg/kg, i.p.) 30 min prior to zymosan injection and were killed after 4 or 18 h. The heatmap shows changes in lipid mediator levels relative to the vehicle control. The labels ALOX12/15, ALOX5, and PTGS indicate products of these enzymes. Individual data from *n* = 5–6 mice relative to the mean of the vehicle control. Data for the vehicle control are identical to Waltl et al.[5].

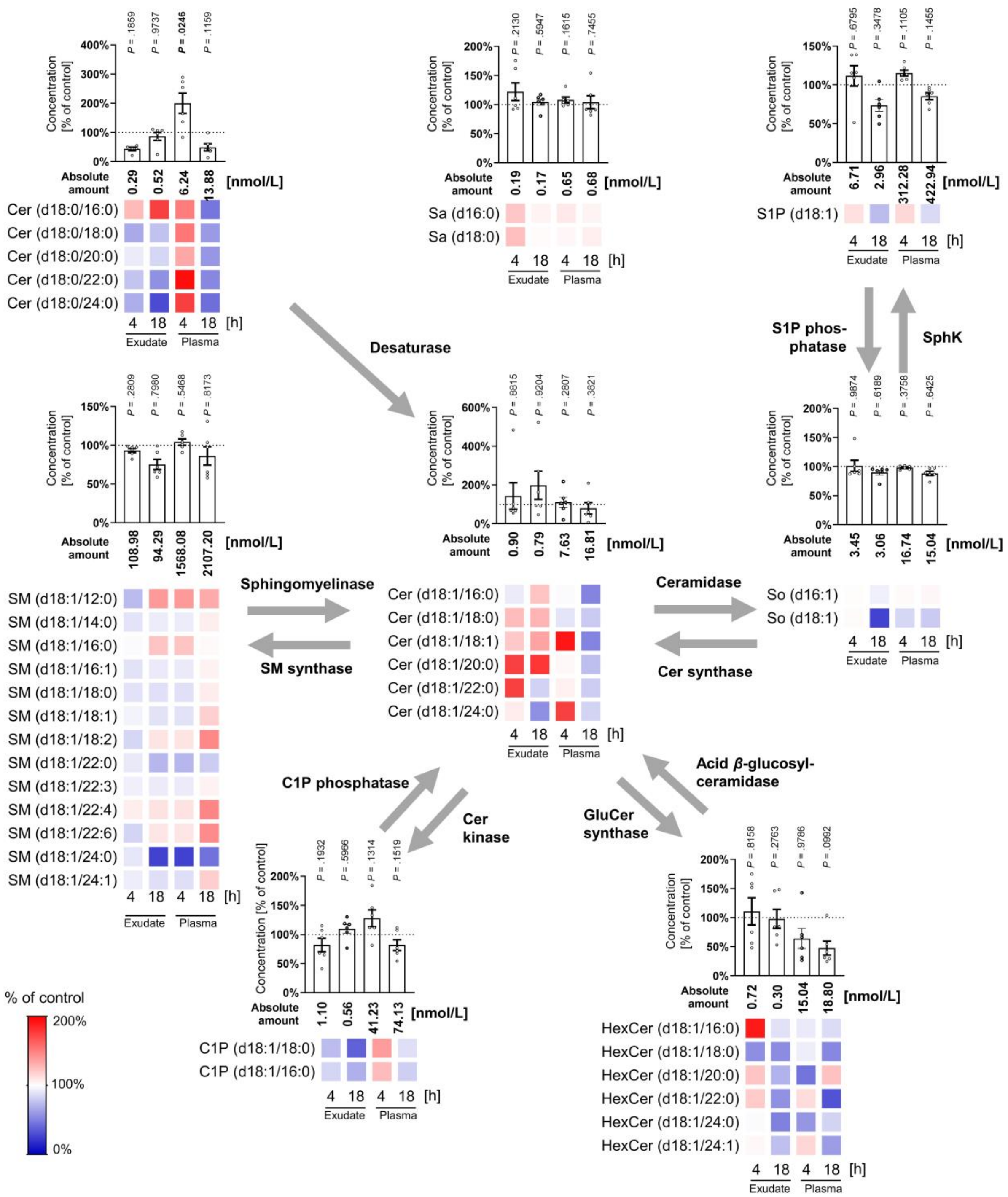

Figure S11 Changes of systemic and exudate sphingolipid metabolism in experimental peritonitis. Mice received vehicle (2% DMSO, ‘w/o’) or **2** (10 mg/kg, i.p.) 30 min prior to zymosan injection and were killed after 4 or 18 h. The bar charts and heatmaps show the percentage changes in the exudate and plasma levels of sphingolipid species (heatmap) or total sphingolipid classes (bar chart) upon administration of **2** relative to vehicle controls. The absolute amounts of the sphingolipid subclasses are given below the respective bars. Reactions and major enzymes in sphingolipid metabolism are highlighted. Mean (heatmap) ± SEM and single data (bar charts) from *n* = 5–6 mice. *P* values *vs.* vehicle-treated animals; unpaired two-sided Student *t*-tests of log data. GluCer synthase, Glycosylceramide synthase, Sa, sphinganine, So, Sphingosine, [dh]CER, (dihydro)ceramide, [dh]SM, (dihydro)sphingomyelin, HexCer, hexosylceramide (HexCer), ceramide-1-phosphate (C1P).

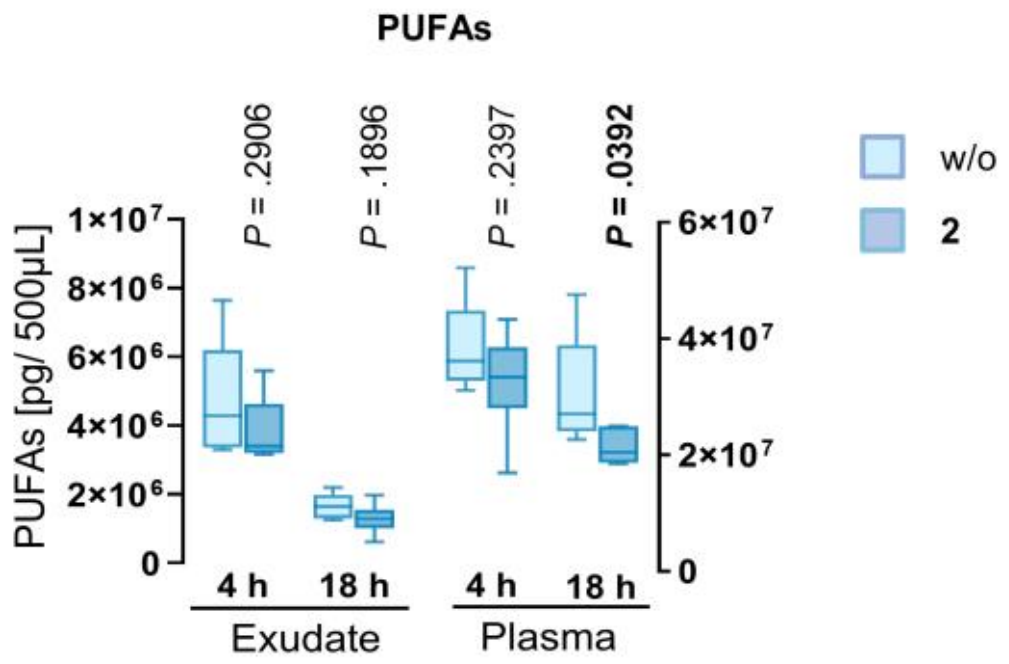

Figure S12 LCM **2** does not elevate free PUFA levels in mouse peritonitis. Mice received vehicle (2% DMSO, 'w/o') or **2** (10 mg/kg, i.p.) 30 min prior to zymosan injection and were killed after 4 or 18 h. Levels of free PUFAs in the exudate and plasma are presented. Mean with boxes showing the 25th to 75th percentiles and whiskers indicating smallest and largest values from *n* = 4–6 mice per group. *P* values *vs.* vehicle control; unpaired Student *t* test. Data for vehicle are identical to Waltl et al.[5].

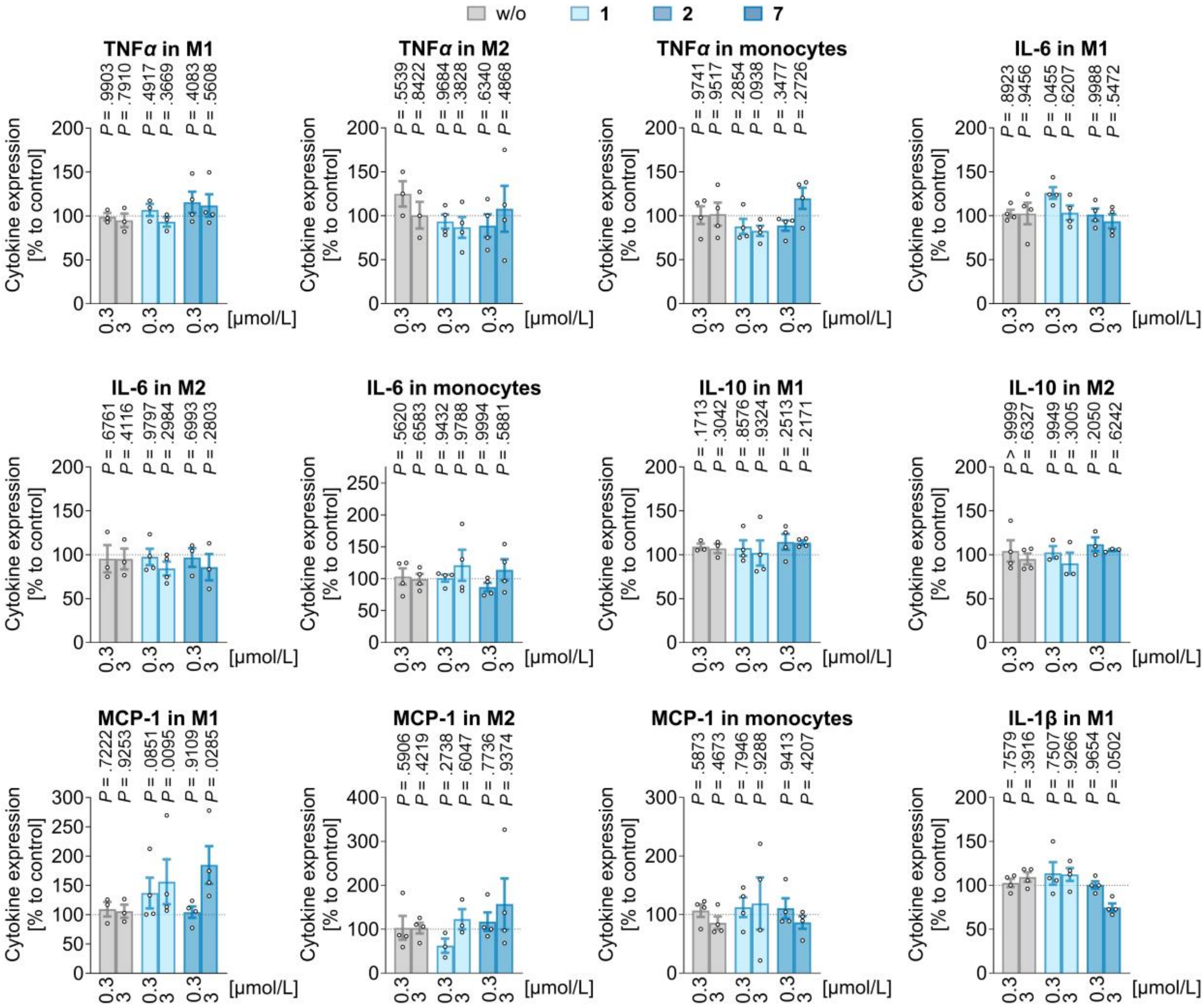

Figure S13 Effect of LCMs on cytokine production in human immune cells. Monocytes (3×10^6) or monocyte-derived macrophages (2×10^6) were preincubated with vehicle (DMSO, 0.1%, 'w/o') or LCMs (0.3 and 3 µmol/L) for 15 min. Monocytes were then stimulated with LPS (100 ng/mL) for 24 h, whereas macrophages were polarized to M1-like macrophages (20 ng/mL interferon-γ and 100 ng/mL LPS) or M2-like macrophages (IL-4) for 48 h. Cytokine levels are shown for TNFα, IL-6, IL10, MCP-1, and IL-1β. Absolute cytokine concentrations of vehicle control (100%) are 72.4 ± 7.8 ng/mL (M1), 0.8 ± 0.3 ng/mL (M2) and 7.3 ± 0.7 ng/mL (monocytes) for TNFα, 79.8 ± 8.9 ng/mL (M1), 1.6 ± 0.5 ng/mL (M2) and 55.9 ± 2.9 ng/mL (monocytes) for IL-6, 0.76 ± 0.16 ng/mL (M1) and 0.35 ± 0.04 ng/mL (M2) for IL-10, 49.3 ± 6.0 ng/mL (M1), 72.6 ± 20.6 ng/mL (M2) and 8.0 ± 0.8 ng/mL (monocytes) for MCP-1, and 0.22 ± 0.06 ng/mL (M1) for IL-1β. Mean ± SEM and single data from n = 3–4 independent experiments. *P* values *vs.* vehicle-treated cells; repeated measures one-way ANOVAs with Dunnett's *post hoc* tests.

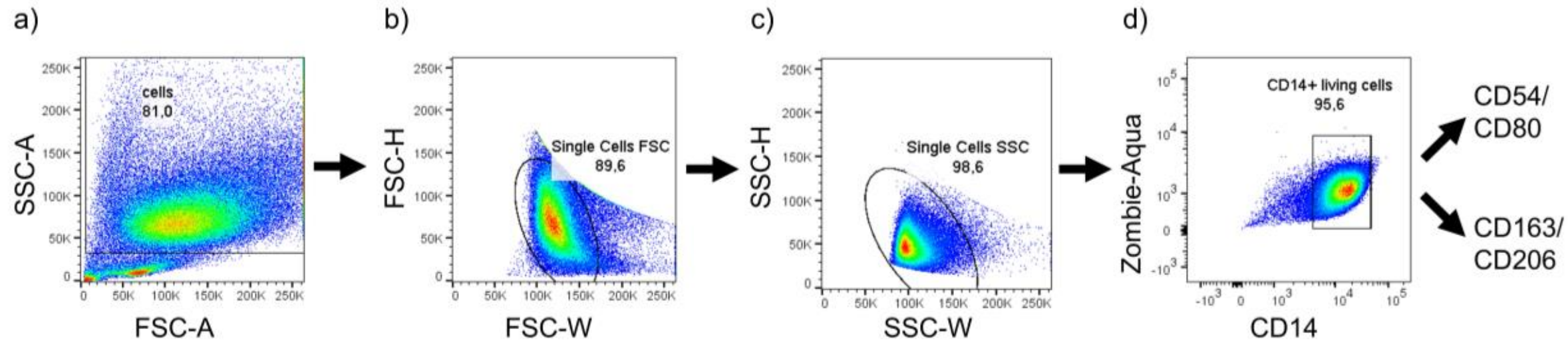

Figure S14 Gating strategy for the flow cytometric analysis of monocyte-derived macrophages.

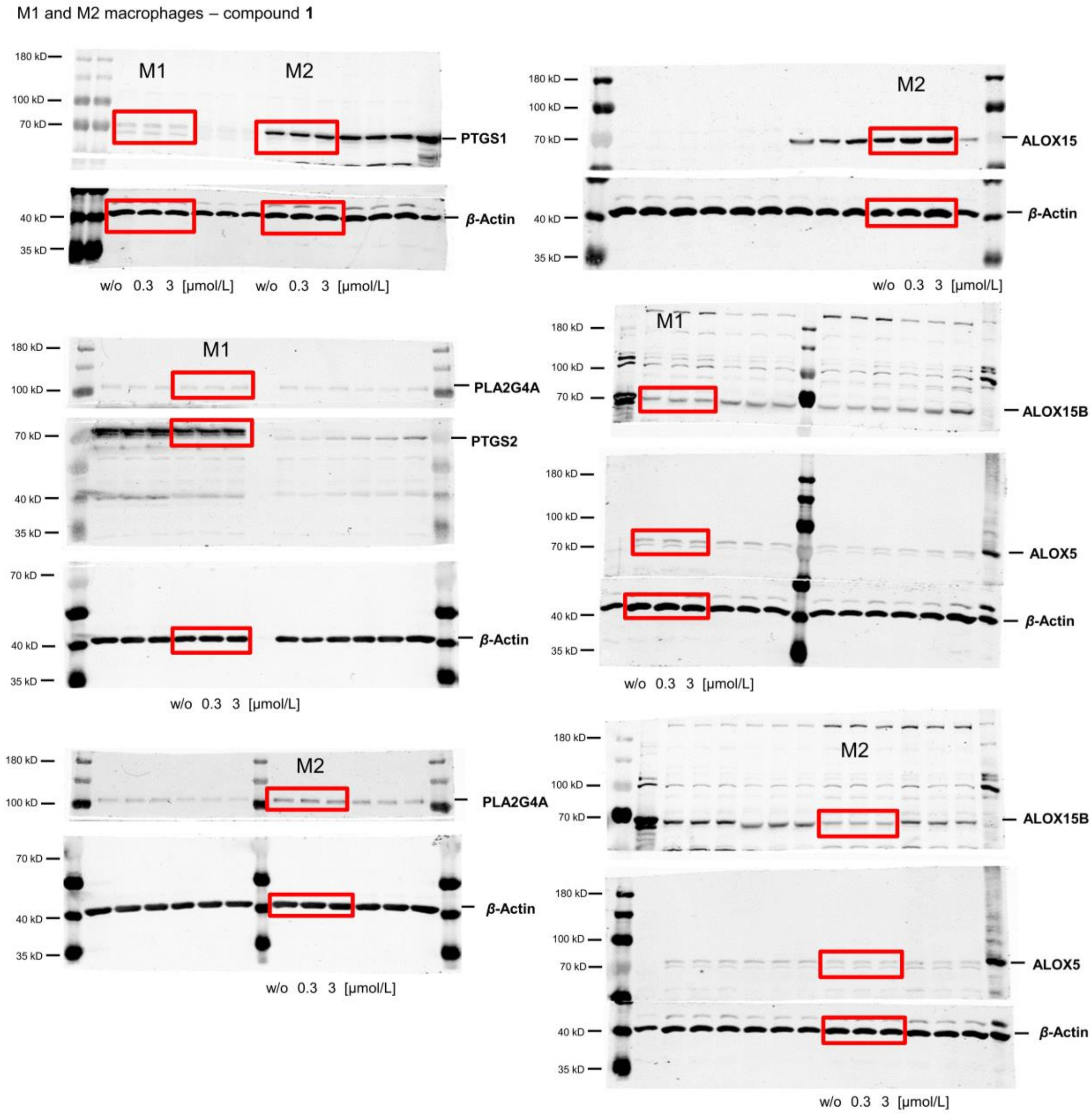

Figure S15 Uncropped versions of the Western blots for **1**-treated M1 and M2 macrophages shown in Fig. 7E and Supporting Information Fig. S9.

M1 and M2 macrophages – compound **2**

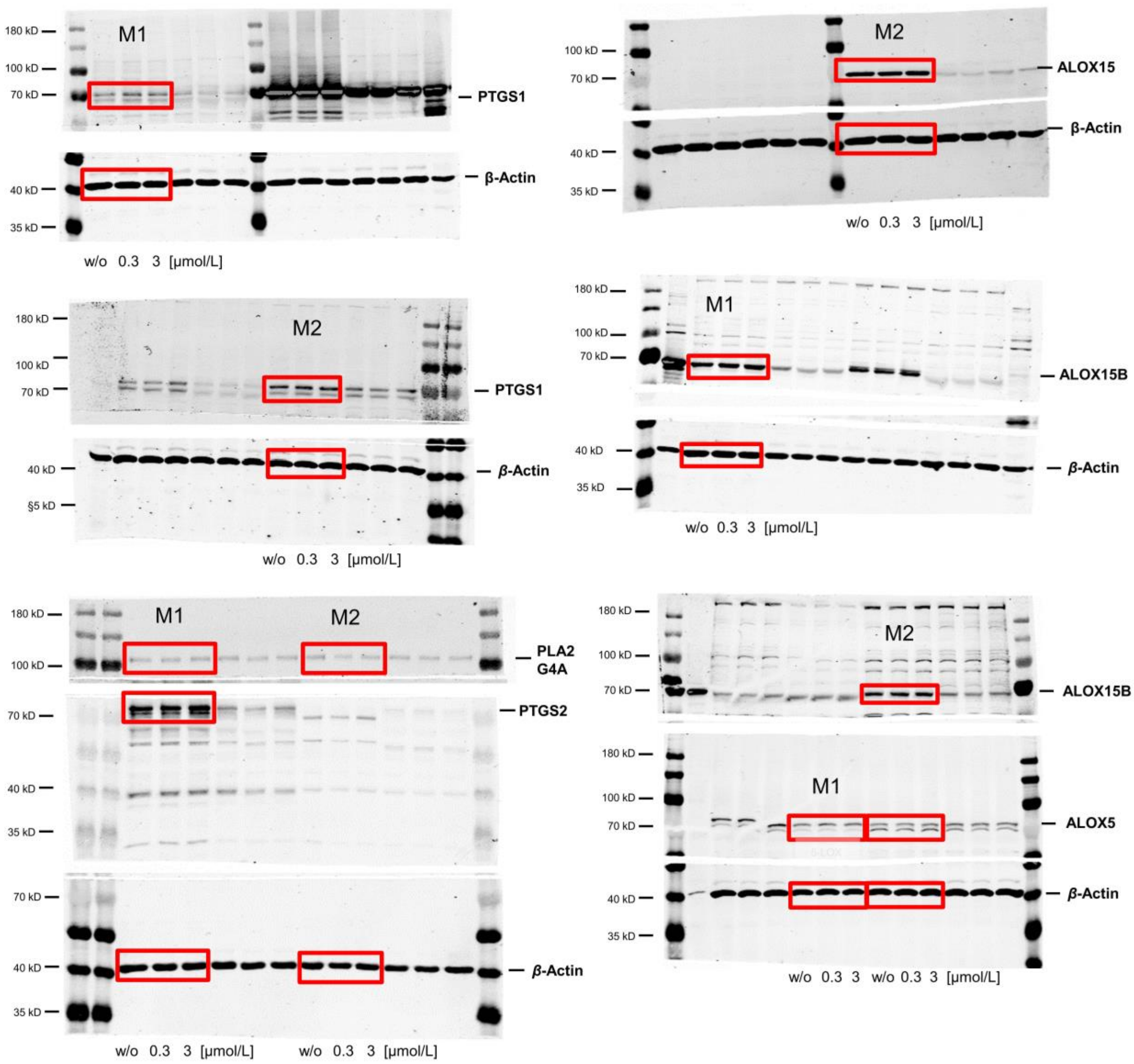

Figure S16 Uncropped versions of the Western blots for **2**-treated M1 and M2 macrophages shown in Fig. 7E and Supporting Information Fig. S9.

M1 and M2 macrophages – compound **7**

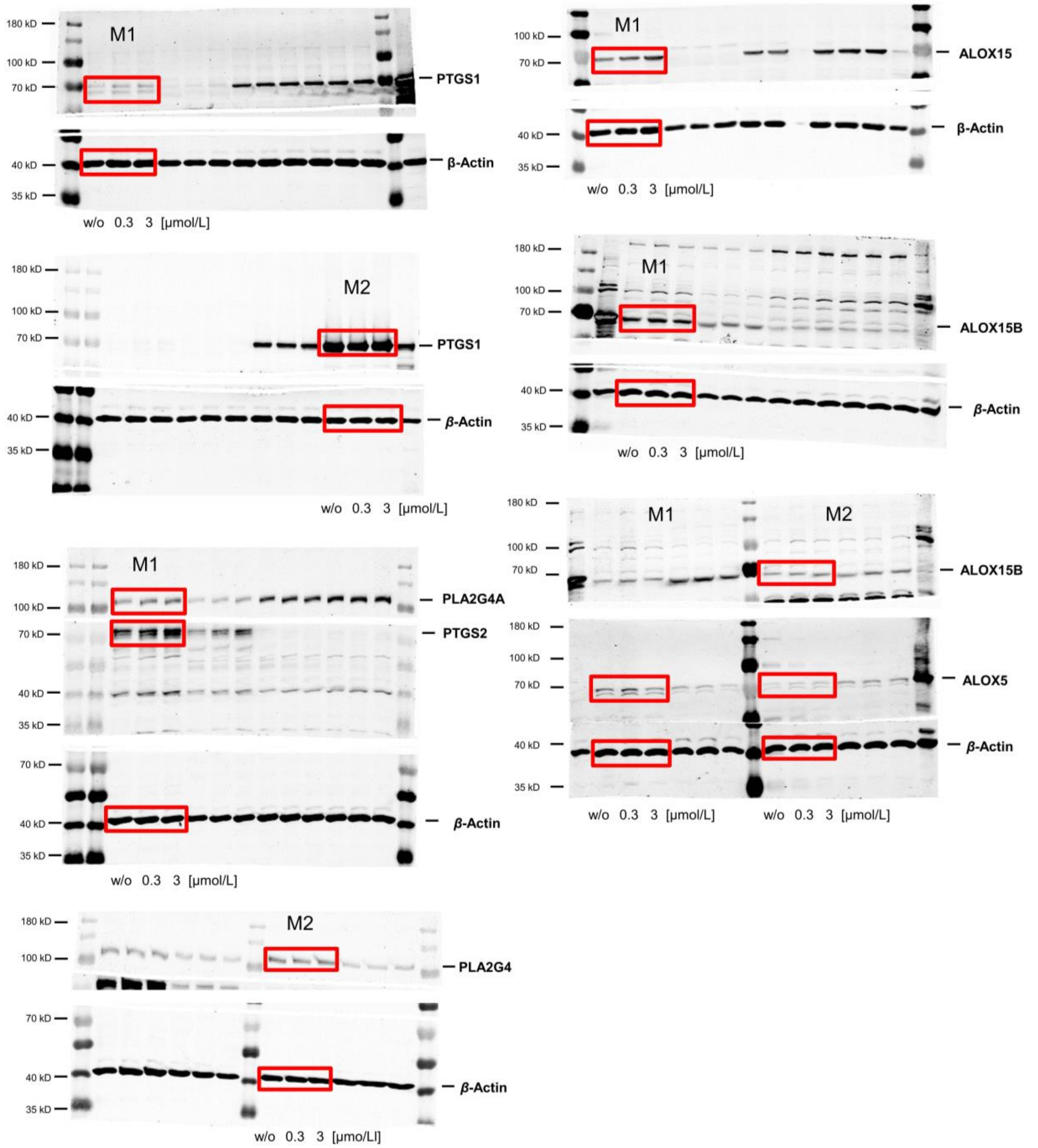

Figure S17 Uncropped versions of the Western blots for **7**-treated M1 and M2 macrophages shown in Fig. 7E and Supporting Information Fig. S9.

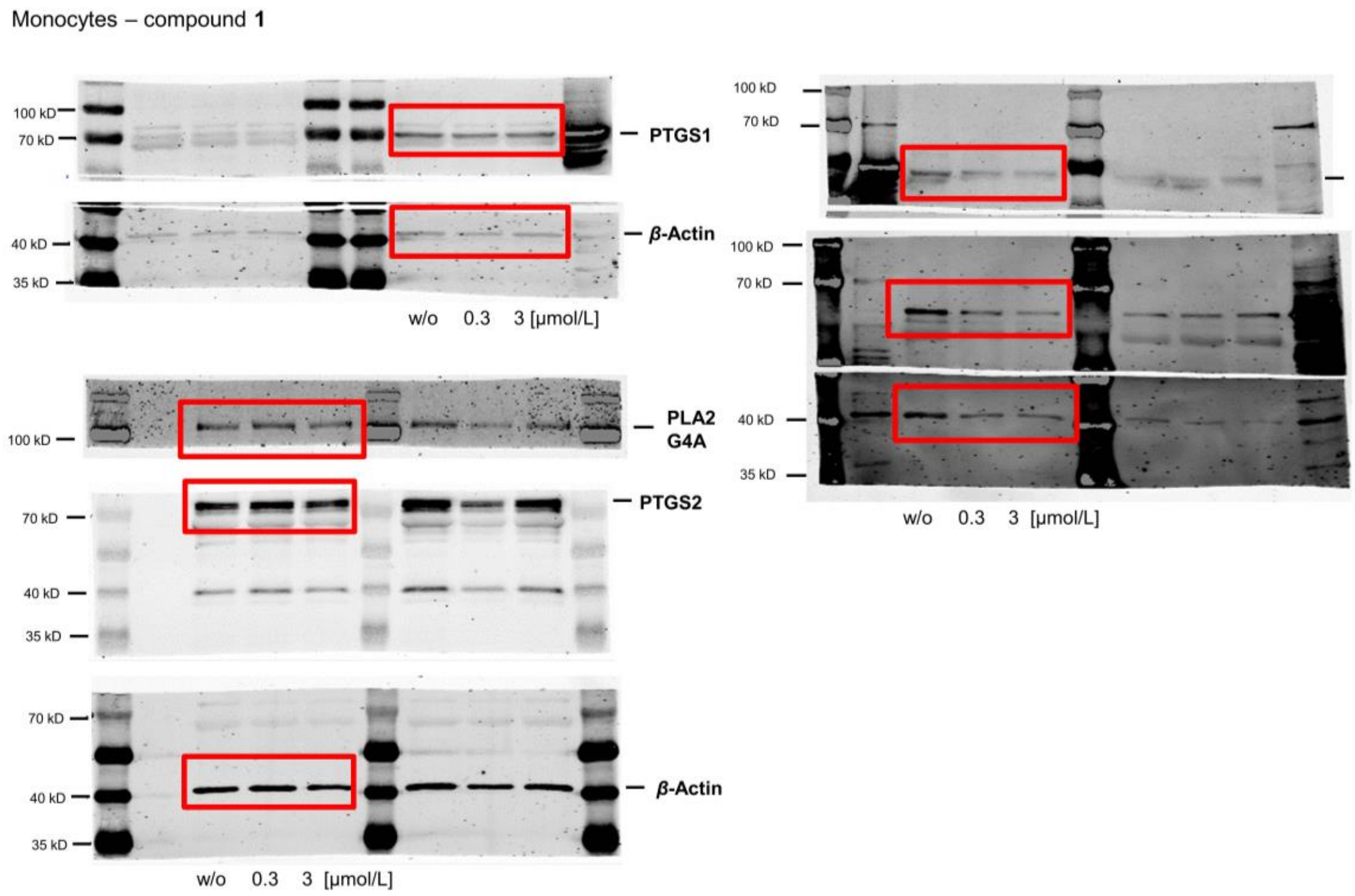

Figure S18 Uncropped versions of the Western blots for **1**-treated monocytes shown in Supporting Information Fig. S9.

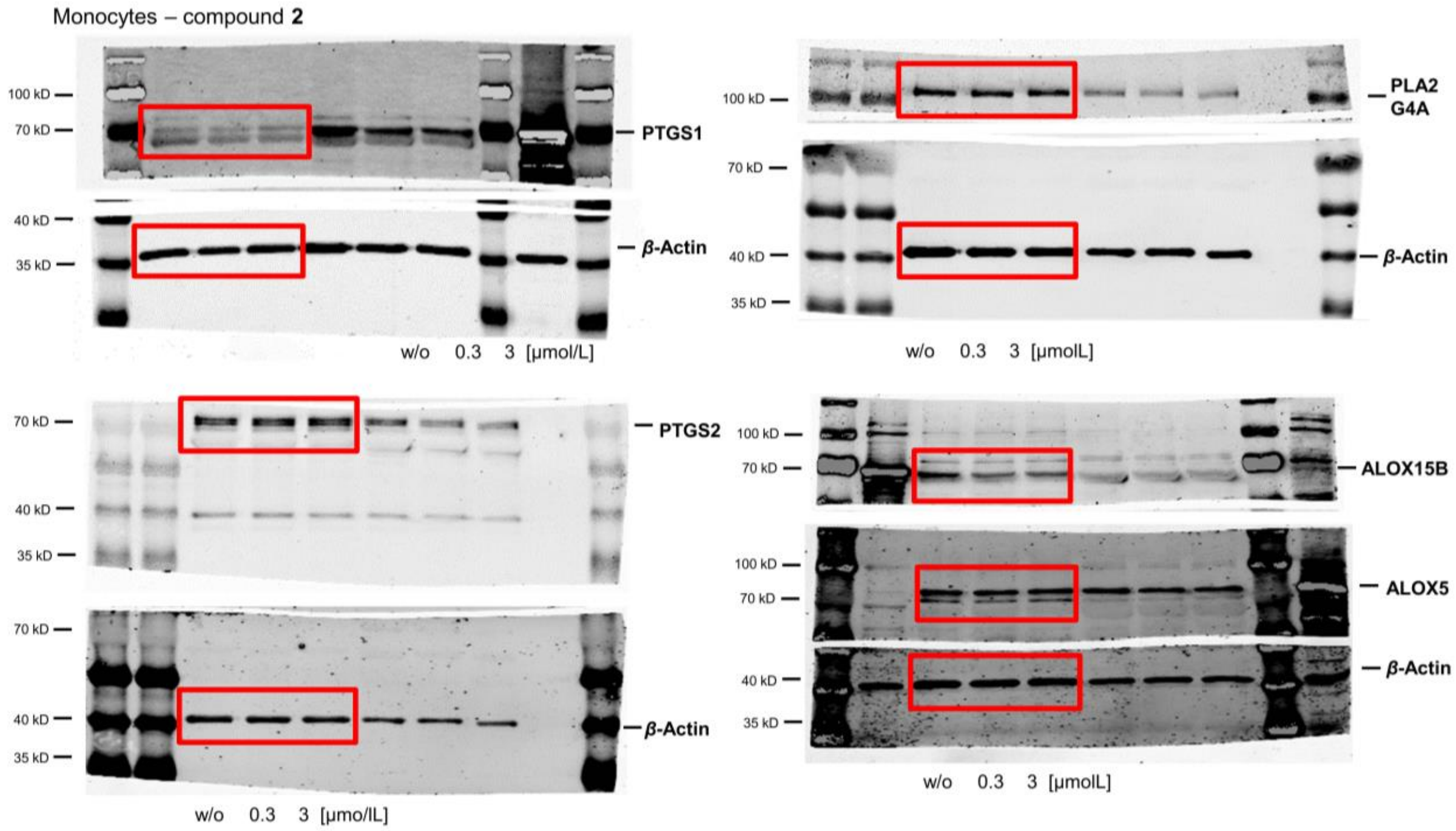

Figure S19 Uncropped versions of the Western blots for **2**-treated monocytes shown in Supporting Information Fig. S9.

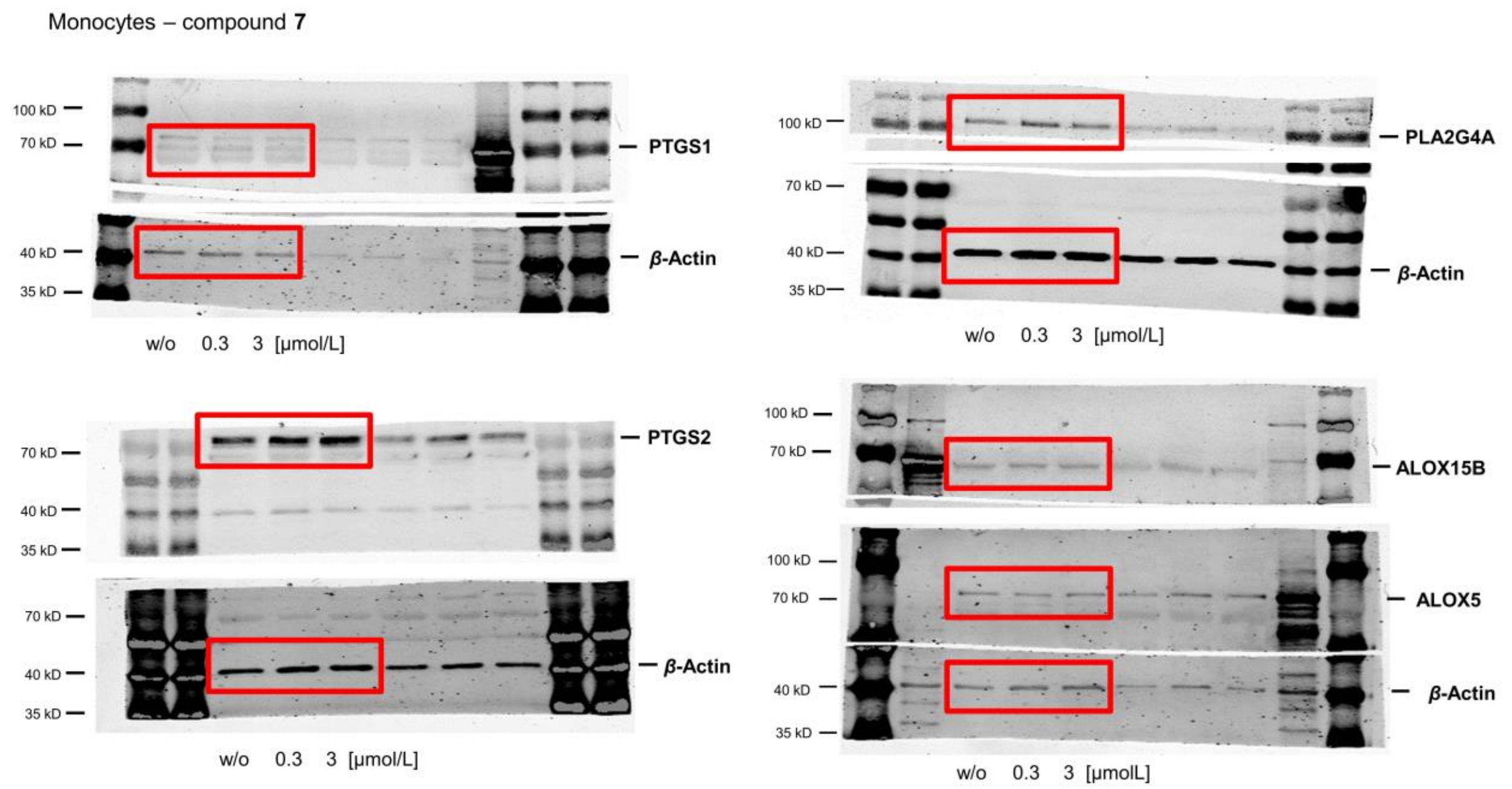

Figure S20 Uncropped versions of the Western blots for **7**-treated monocytes shown in Supporting Information Fig. S9.

Supplementary references

1 Birringer M, Lington D, Vertuani S, Manfredini S, Scharlau D, Glei M, et al. Proapoptotic effects of long-chain vitamin E metabolites in HepG2 cells are mediated by oxidative stress. *Free Radic Biol Med* 2010;**49**:1315-22.

2 Richomme P, Helesbeux JJ, Guilet D, Seraphin D, Stuppner H, Waltenberger B, et al., Inventors. Tocotrienol derivatives, pharmaceutical composition and method of use in 5-lipoxygenase related diseases. WO2017032881A1, 2017.

3 Neukirch K, Alsabil K, Dinh CP, Bilancia R, Raasch M, Ville A, et al. Exploration of long-chain vitamin E metabolites for the discovery of a highly potent, orally effective, and metabolically stable 5-LOX inhibitor that limits inflammation. *J Med Chem* 2021;**64**:11496-526.

4 Pein H, Ville A, Pace S, Temml V, Garscha U, Raasch M, et al. Endogenous metabolites of vitamin E limit inflammation by targeting 5-lipoxygenase. *Nat Commun* 2018;**9**:3834.

5 Waltl L, Speck K, Wildermuth R, Haut FL, Permann S, D'Avino D, et al. Reorganization of innate immune cell lipid profiles by bioinspired meroterpenoids to limit inflammation. *bioRxiv* 2024. Available from: https://doi.org/10.1101/2024.05.24.595516.
